# Loss of global DNA hypermethylation is prognostic in IDH-mutant and 1p/19q-codeleted oligodendrogliomas

**DOI:** 10.1007/s00401-025-02963-7

**Published:** 2025-11-26

**Authors:** Felix E. Hinz, Dennis Friedel, Franziska M. Ippen, Martin Sill, Andrey Korshunov, Leonille Schweizer, Daniel Schrimpf, Kirsten Göbel, Lukas S. Friedrich, Fuat K. Aras, Henri Bogumil, Rouzbeh Banan, Hildegard Dohmen, Till Acker, Sebastian Brandner, Simone Schmid, David Capper, Niklas Grassl, Henning B. Boldt, Pieter Wesseling, Sybren L. N. Maas, Juan P. Garces Martinez, Christine Stadelmann, Guido Reifenberger, Thomas Stehle, Alonso Barrantes-Freer, Tareq A. Juratli, Stefan Pusch, Daniel Haag, David E. Reuss, Christel Herold-Mende, Sandro Krieg, Wolfgang Wick, Nima Etminan, Michael Platten, Stefan M. Pfister, David T. W. Jones, Felix Sahm, Andreas von Deimling, Abigail K. Suwala

**Affiliations:** 1 Department of Neuropathology, Pathological Institute, Heidelberg University Hospital, Heidelberg, Germany; 2 Clinical Cooperation Unit Neuropathology, German Consortium for Translational Cancer Research (DKTK), German Cancer Research Center (DKFZ), Heidelberg, Germany; 3 Faculty of Bioscience, Heidelberg University, Heidelberg, Germany; 4 A Partnership Between DKFZ and University Hospital Heidelberg, National Center for Tumor Diseases (NCT), NCT Heidelberg, Heidelberg, Germany; 5 Department of Neurology and European Center for Neurooncology (EZN), University Hospital Heidelberg, Heidelberg, Germany; 6 Hopp Children’s Cancer Center Heidelberg (KiTZ), Heidelberg, Germany; 7 Division of Pediatric Neurooncology, German Cancer Consortium (DKTK), German Cancer Research Center (DKFZ), Heidelberg, Germany; 8 Neurological Institute (Edinger Institute), Goethe University, Frankfurt, Germany; 9 German Cancer Consortium (DKTK), Partner Site Frankfurt, German Cancer Research Center (DKFZ), Frankfurt, Germany; 10 University Cancer Center, Goethe University Frankfurt, Frankfurt, Germany; 11 Frankfurt Cancer Institute (FCI), Frankfurt, Germany; 12 Institute of Pathology Nordhessen, Kassel, Germany; 13 Institute of Neuropathology, Justus-Liebig University Giessen, Giessen, Germany; 14 Institute of Neurology, University College London, London, UK; 15 Department of Neuropathology, Charité-Universitätsmedizin Berlin, Corporate Member of Freie Universität Berlin and Humboldt-Universität Zu Berlin, Berlin, Germany; 16 German Cancer Consortium (DKTK), Partner Site Berlin, German Cancer Research Center (DKFZ), Heidelberg, Germany; 17 DKTK CCU Neuroimmunology and Brain Tumor Immunology, German Cancer Research Center (DKFZ), Heidelberg, Germany; 18 Department of Neurology, Medical Faculty Mannheim, MCTN, Heidelberg University, Mannheim, Germany; 19 DKFZ-Hector Cancer Institute at University Medical Center Mannheim, Mannheim, Germany; 20 Department of Pathology, Odense University Hospital, Odense, Denmark; 21 Department of Pathology, Amsterdam University Medical Centers, Amsterdam and Princess Máxima Center for Pediatric Oncology, Utrecht, The Netherlands; 22 Department of Pathology, Leiden University Medical Center, Leiden, The Netherlands; 23 Department of Pathology, Erasmus MC Cancer Institute, Rotterdam, The Netherlands; 24 Department of Neuropathology, University Medical Center Göttingen, Göttingen, Germany; 25 Institute of Neuropathology, Partner Site Essen/Düsseldorf, Medical Faculty, and University Hospital Düsseldorf, German Cancer Consortium (DKTK), Heinrich Heine University, Düsseldorf, Germany; 26 Institute for Neuropathology, Faculty of Medicine, University Hospital Cologne, Cologne, Germany; 27 University of Leipzig Medical Center, Paul-Flechsig-Institute of Neuropathology, Leipzig, Germany; 28 Department for Neurosurgery, TU Dresden University of Technology, Dresden, Germany; 29 Department of Neurosurgery, Heidelberg University Hospital, Heidelberg, Germany; 30 Clinical Cooperation Unit Neurooncology, German Consortium for Translational Cancer Research (DKTK), German Cancer Research Center (DKFZ), Heidelberg, Germany; 31 Department of Neurosurgery, Heidelberg University, Mannheim, Germany; 32 Division of Pediatric Glioma Research, German Cancer Research Center (DKFZ), Heidelberg, Germany

Diffuse isocitrate dehydrogenase (IDH)-mutant gliomas represent the most common malignant primary brain tumours in adults under the age of 50 years [7]. According to the 2021 World Health Organization (WHO) classification of central nervous system (CNS) tumours, these gliomas are further stratified into two major types: astrocytoma, IDH-mutant, graded as CNS WHO grades 2, 3 or 4 and oligodendroglioma, IDH-mutant and 1p/19q-codeleted, graded as CNS WHO grades 2 or 3 [5, 9].

With the initial publication of the Heidelberg CNS tumour methylation classifier in 2018 [1], DNA methylation became an important tool in neuropathological workup due to its ability to provide highly accurate tumour classification independent of histopathological evaluation. In addition to classifying tumours, methylation arrays can simultaneously detect copy number variations (CNVs) relevant for diagnosis and grading, including 1p/19q codeletion and *CDKN2A/B* homozygous deletion.

In version 12.8 of the Heidelberg CNS tumour methylation classifier, oligodendrogliomas are assigned to a single methylation class (MC), while astrocytomas are subdivided into two distinct MCs: astrocytoma, IDH-mutant, lower grade and astrocytoma, IDH-mutant, high grade*.* These MCs overlap with the previously characterized G-CIMP-high (MC astrocytoma, IDH-mutant, lower grade) and G-CIMP-low (MC astrocytoma, IDH-mutant, high grade) profiles [1, 4, 6, 8, 12, 14]. Importantly, these MCs correlate with divergent biological behaviours and clinical outcomes [6, 8]. IDH-mutant astrocytomas corresponding to the MC astrocytoma, IDH-mutant, high grade, which exhibit lower levels of global DNA methylation, are more frequently assigned to CNS WHO grade 4 and are associated with significantly reduced overall survival compared to IDH-mutant astrocytomas corresponding to the MC astrocytoma, IDH-mutant, lower grade [3, 13]. Oligodendrogliomas, IDH-mutant and 1p/19q-codeleted, exhibit the highest levels of global DNA methylation among IDH-mutant gliomas [11].

In recent routine diagnostic workflows, we observed a subset of IDH-mutant gliomas with confirmed 1p/19q codeletion (Supplementary Data 1) that were unexpectedly classified into one of the astrocytoma MCs by the Heidelberg CNS tumour classifier v12.8 [Supplementary Table 1]. These tumours harboured genetic alterations typically found in oligodendrogliomas, IDH-mutant and 1p/19q-codeleted (including mutations in *IDH1*, *CIC*, *FUBP1* and *TERTp*, Supplementary Table 2). In accordance with WHO criteria, these tumours were diagnosed as oligodendroglioma, IDH-mutant and 1p/19q-codeleted, despite their astrocytoma-like DNA methylation profiles. Given the association between lower global DNA methylation and worse prognosis in IDH-mutant astrocytomas, we hypothesized that these classifier results might reflect oligodendrogliomas with decreased global methylation, potentially indicative of adverse clinical outcomes.

To explore this, we identified tumours with the highest score for the methylation family class diffuse glioma, IDH-mutant, and the highest class prediction matching either of the two astrocytoma MCs, using classifier version 12.8 or earlier versions. Given the central role of IDH mutations in shaping DNA methylation profiles, we were confident that this approach would reliably enrich for IDH-mutant tumours. After excluding samples without 1p/19q codeletion, we assembled a cohort comprising 69 IDH-mutant and 1p/19q-codeleted tumours, among these 27 tumours with assignment to MC astrocytoma, IDH-mutant, lower grade (O_MC_Astro_LG, *n* = 19 with available clinical data; 17/19 [89%] classified as CNS WHO grade 2) and 42 tumours with assignment to MC astrocytoma, IDH-mutant, high grade (O_MC_Astro_HG, *n* = 21 with available clinical data; 19/21 [90%] classified as CNS WHO grade 3) [Supplementary Table 1].

We compared the mean global DNA methylation levels of these tumours to establish reference groups, revealing a significant reduction in global methylation levels relative to oligodendrogliomas, IDH-mutant and 1p/19q-codeleted assigned to the oligodendroglioma MC (Fig. 1b). To investigate whether the apparent hypomethylation was caused by lower tumour cell content, we performed deconvolution analysis using a modified version of MethylCIBERSORT [2] (https://bioconductor.org/packages/release/bioc/html/EpiDISH.html). This analysis demonstrated high tumour cell content for oligodendrogliomas in the MC astrocytoma, IDH-mutant, high-grade, whereas the oligodendrogliomas in the MC astrocytoma, IDH-mutant, lower grade displayed a lower fraction of neoplastic cells compared to the other MCs analysed (Supplementary Fig. 1). Consequently, the 1p/19q codeletion was less pronounced (Supplementary Data 1) and the scores were generally lower, whereas high grades had matching scores for diffuse glioma, IDH-mutant of at least (0.8), ranging from 0.53 to 0.99 for MC astrocytoma, IDH-mutant, high grade. Thus, the reduced global DNA hypermethylation levels detected in oligodendrogliomas assigned to the astrocytoma, IDH-mutant, lower grade MC were likely due to an increased fraction of non-neoplastic cells and therefore not considered informative for classification or grading.

**Fig. 1:**
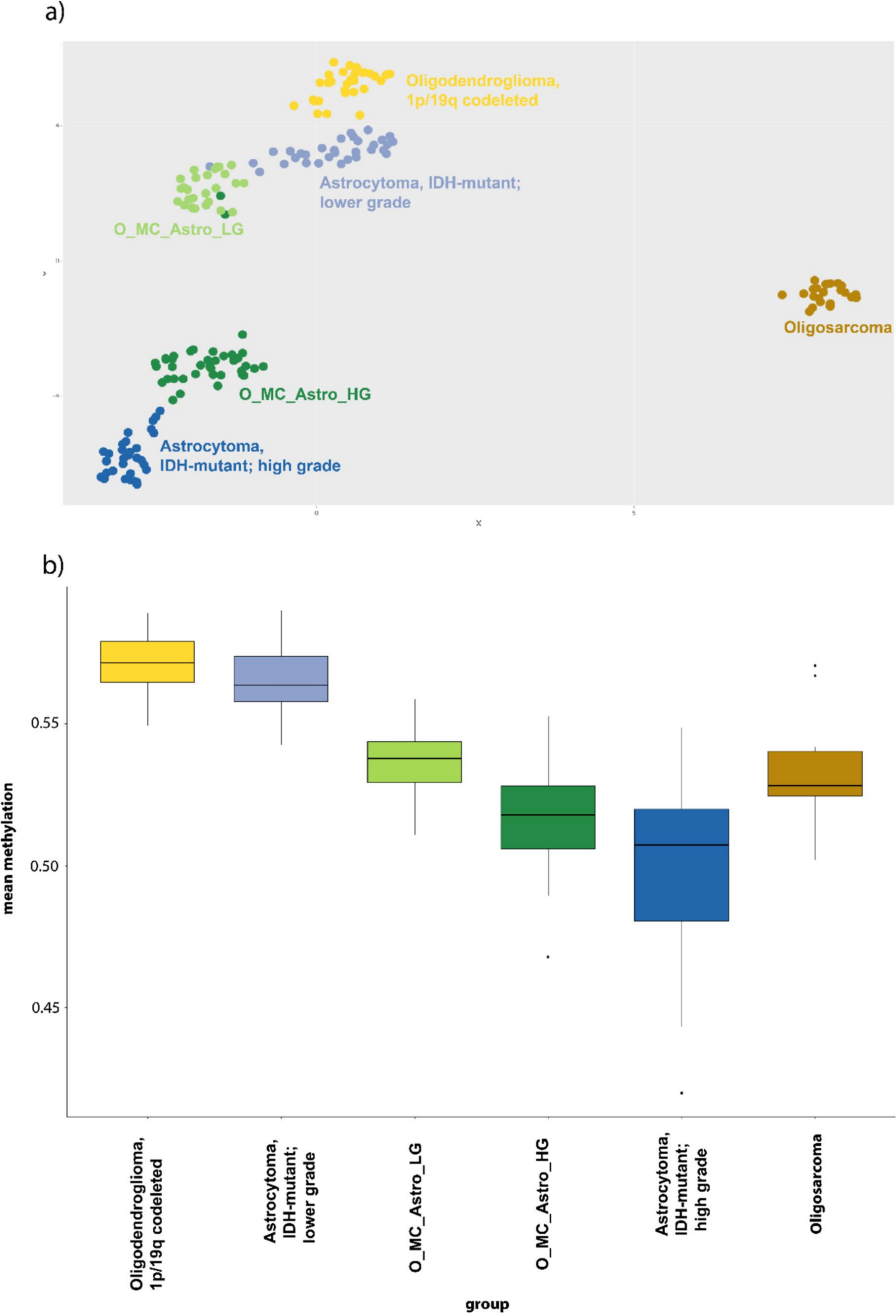
Global DNA methylation differences in IDH-mutant gliomas. **a** UMAP analysis of *n* = 183 cases based on the 25,000 most variable CpG sites. IDH-mutant and 1p/19q-codeleted oligodendrogliomas assigned to either of the two astrocytomas, IDH-mutant MCs form distinct clusters, separate from IDH-mutant and 1p/19q-codeleted oligodendrogliomas, and IDH-mutant astrocytomas correctly diagnosed by DNA methylation prediction. **b** IDH-mutant and 1p/19q-codeleted oligodendrogliomas assigned to one of the two IDH-mutant astrocytoma MCs exhibit reduced global DNA methylation levels compared to IDH-mutant and 1p/19q-codeleted oligodendrogliomas assigned to the oligodendroglioma MC. Abbr.: O_MC_Astro_HG, oligodendrogliomas with the MC astrocytoma, IDH-mutant, high grade; O_MC_Astro_LG, oligodendrogliomas with the MC astrocytoma, IDH-mutant, lower grade

We then compared progression-free survival (PFS) and overall survival (OS) in patients with IDH-mutant and 1p/19q-codeleted oligodendrogliomas assigned to an IDH-mutant astrocytoma MC with OS of patients with IDH-mutant and 1p/19q-codeleted oligodendrogliomas CNS WHO grade 2 or 3 assigned to the oligodendroglioma MC. Based on the MC assignment of newly diagnosed tumours, patients with IDH-mutant and 1p/19q-codeleted oligodendrogliomas assigned to the MC astrocytoma, IDH-mutant, high grade, demonstrated significantly worse OS compared to patients diagnosed with IDH-mutant and 1p/19q-codeleted oligodendrogliomas assigned to the MC astrocytoma, IDH-mutant, lower grade, and patients with CNS WHO grade 2 or 3 oligodendrogliomas assigned to the MC oligodendroglioma (Supplementary Fig. 2).

Since our cohort of O_MC_Astro_LG and O_MC_Astro_HG patients included both patients with newly diagnosed and recurrent tumours, we additionally compared OS between recurrent and primary cases, maintaining a similar ratio of recurrent to primary tumours across comparison groups (oligodendrogliomas in the MC astrocytoma, IDH-mutant, high grade: 11/10; reference grade 3 oligodendrogliomas: 18/17) (Fig. 2). No significant differences in PFS were observed (Supplementary Fig. 3).

**Fig. 2:**
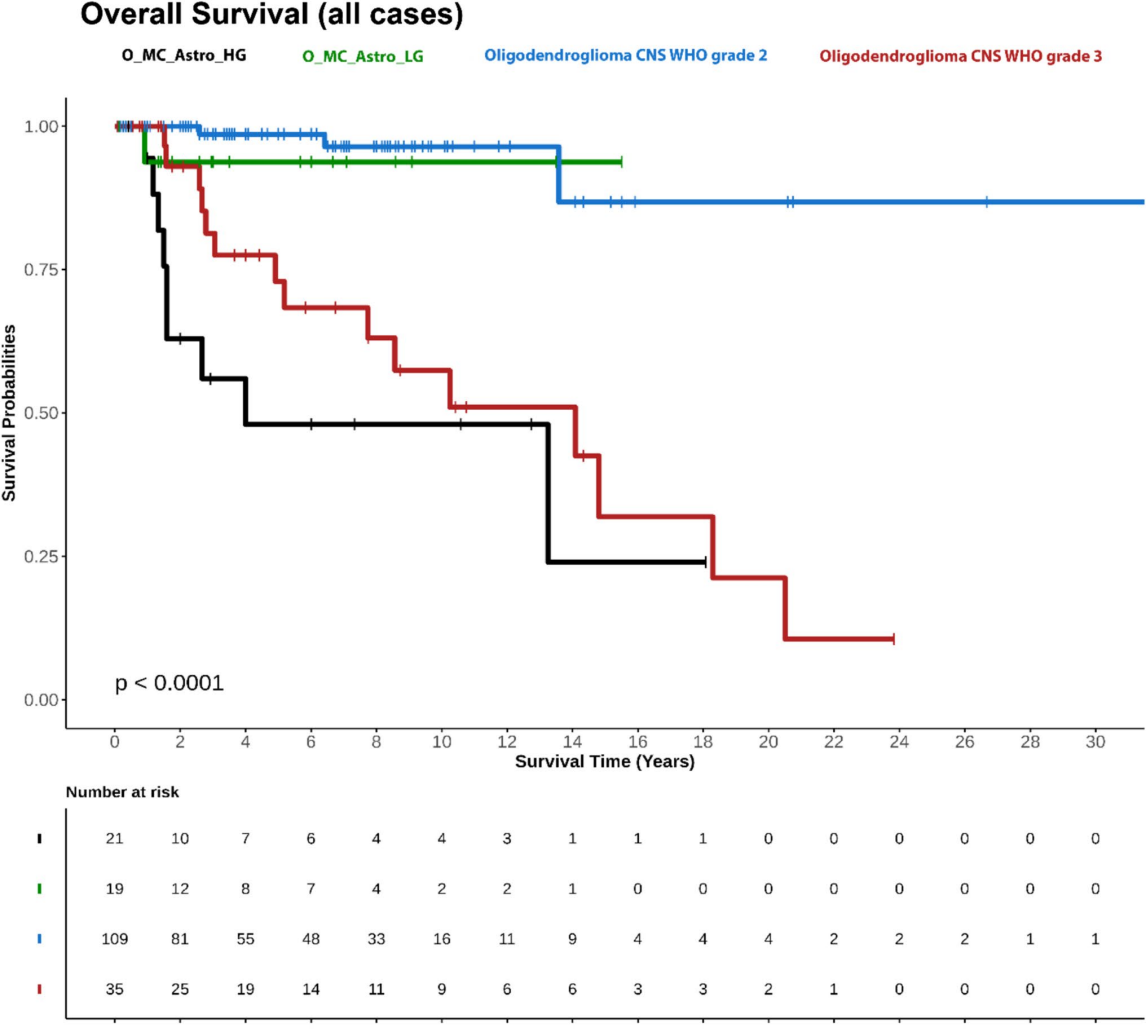
Overall survival of patients with oligodendrogliomas, IDH-mutant and 1p/19q-codeleted stratified according to MC assignment. Comparison of all patients with IDH-mutant and 1p/19q-codeleted oligodendrogliomas exhibiting an IDH-mutant astrocytoma subtype methylation profile with patients diagnosed with IDH-mutant and 1p/19q-codeleted oligodendrogliomas, CNS WHO grade 2 or 3, exhibiting an oligodendroglioma methylation profile. In contrast to Supplementary Fig. 2, the cohorts of patients with oligodendrogliomas, IDH-mutant and 1p/19q-codeleted, CNS WHO grade 3, O_ Astro _HG and O_ Astro _LG, included both patients with primary or recurrent tumours. The ratio was adapted to be similar for patients with oligodendrogliomas, IDH-mutant and 1p/19q-codeleted, CNS WHO grade 3 and O_ Astro _HG. P values (logrank test): O_MC_Astro_LG vs. Oligodendroglioma CNS WHO grade 2: *p* = 0.4011, O_MC_Astro_LG vs. Oligodendroglioma, IDH-mutant and 1p/19q-codeleted, CNS WHO grade 3: *p* = 0.1497, O_MC_Astro_HG vs. O_MC_Astro_LG: *p* = 0.0167, O_MC_Astro_HG vs. Oligodendroglioma, IDH-mutant and 1p/19q-codeleted, CNS WHO grade 2: *p* < 0.0001, O_MC_Astro_HG vs. Oligodendroglioma, IDH-mutant and 1p/19q-codeleted, CNS WHO grade 3: *p* = 0.0950). O_MC_Astro_HG, oligodendroglioma, IDH-mutant and 1p/19q-codeleted, with assignment to the MC astrocytoma, IDH-mutant, high grade; O_MC_Astro_LG, oligodendroglioma, IDH-mutant and 1p/19q-codeleted, with assignment to the MC astrocytoma, IDH-mutant, lower grade

The observed loss of DNA hypermethylation in both primary and recurrent tumour samples suggests that this phenomenon is unlikely to be therapy induced and may instead represent an intrinsic biological process linked to more aggressive tumours. In addition, we observed frequent *CDKN2A/B* deletions and *CDK4* amplifications in O_MC_Astro_HG tumours. Notably, the loss of global DNA hypermethylation has also been documented in malignant progression to oligosarcoma, IDH-mutant and 1p/19q-codeleted, a rare newly described subgroup of aggressive IDH-mutant gliomas associated with poorer clinical outcomes [10].

Our findings thus indicate that the loss of global DNA hypermethylation in IDH-mutant and 1p/19q-codeleted oligodendrogliomas is associated with worse survival. It can be assessed by the Heidelberg classifier, with assignment to the methylation class family diffuse IDH-mutant glioma with the highest score for MC astrocytoma, IDH-mutant, high grade, and may serve as a prognostic biomarker to support a CNS WHO grade 3 classification.

## Supplementary Information

Below is the link to the electronic supplementary material.

Supplementary file1 (PDF 11317 KB)

Supplementary file2 (XLSX 20 KB)

Supplementary file3 (XLSX 11 KB)

## Data Availability

The data that support the findings of this study are available from the corresponding author upon reasonable request.
