# Supplementary material for: Loss of global DNA hypermethylation is prognostic in IDH-mutant and 1p/19q-codeleted oligodendrogliomas
Source: Acta Neuropathol. 2025 Nov 26;150(1):56. doi: 10.1007/s00401-025-02963-7 (PMC12657538; doi:10.1007/s00401-025-02963-7)
Supplement: Supplementary file 1 — Supplementary file1 (PDF 11317 KB) [file 401_2025_2963_MOESM1_ESM.pdf]

Supplementary Figure 1

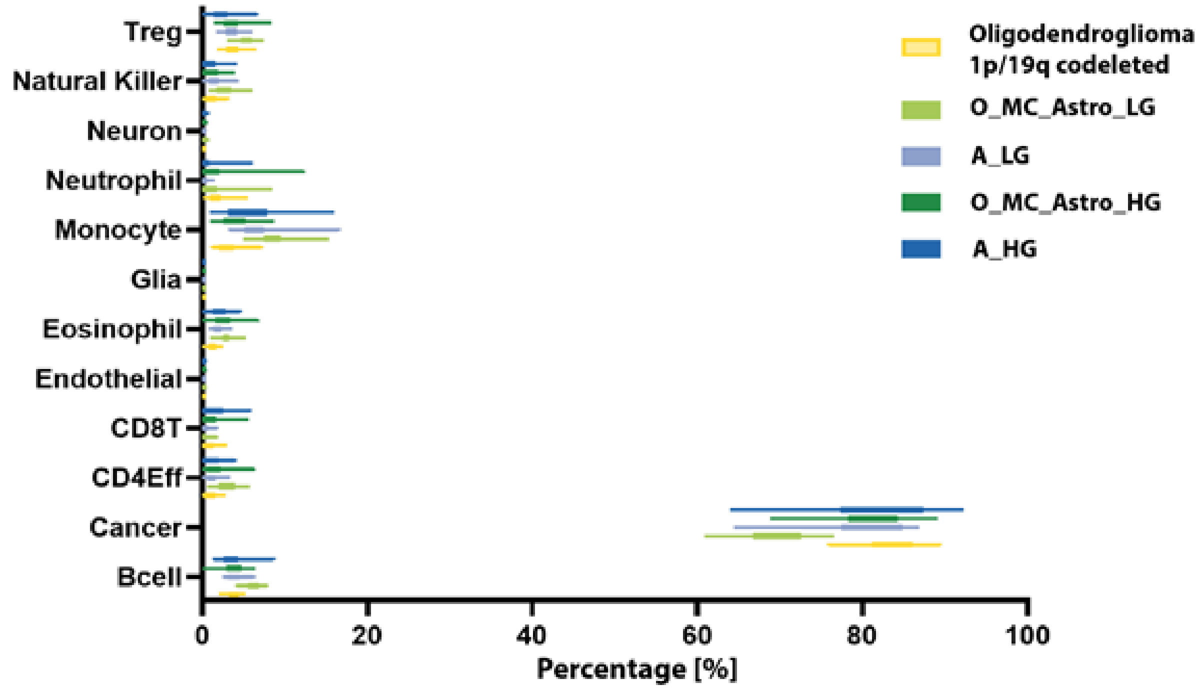

Supplementary Figure 1: Deconvolution based on MethylCIBERSORT. Deconvolution reveals a high percentage of cancer cells in each group and a marginally reduced fraction of cancer cells in MC O_MC_Astro_LG tumours. **Abbr.:** O_MC_Astro_HG, oligodendroglioma, IDH-mutant and 1p/19q-codeleted, with assignment to the MC astrocytoma, IDH-mutant, high grade; O_MC_Astro_LG, oligodendroglioma, IDH-mutant and 1p/19q-codeleted, with assignment to the MC astrocytoma, IDH-mutant, lower grade, A_LG: astrocytoma with assignment to the MC astrocytoma, IDH-mutant, lower grade, A_HG: astrocytoma with assignment to the MC astrocytoma, IDH-mutant, high grade

Supplementary Fig. 2

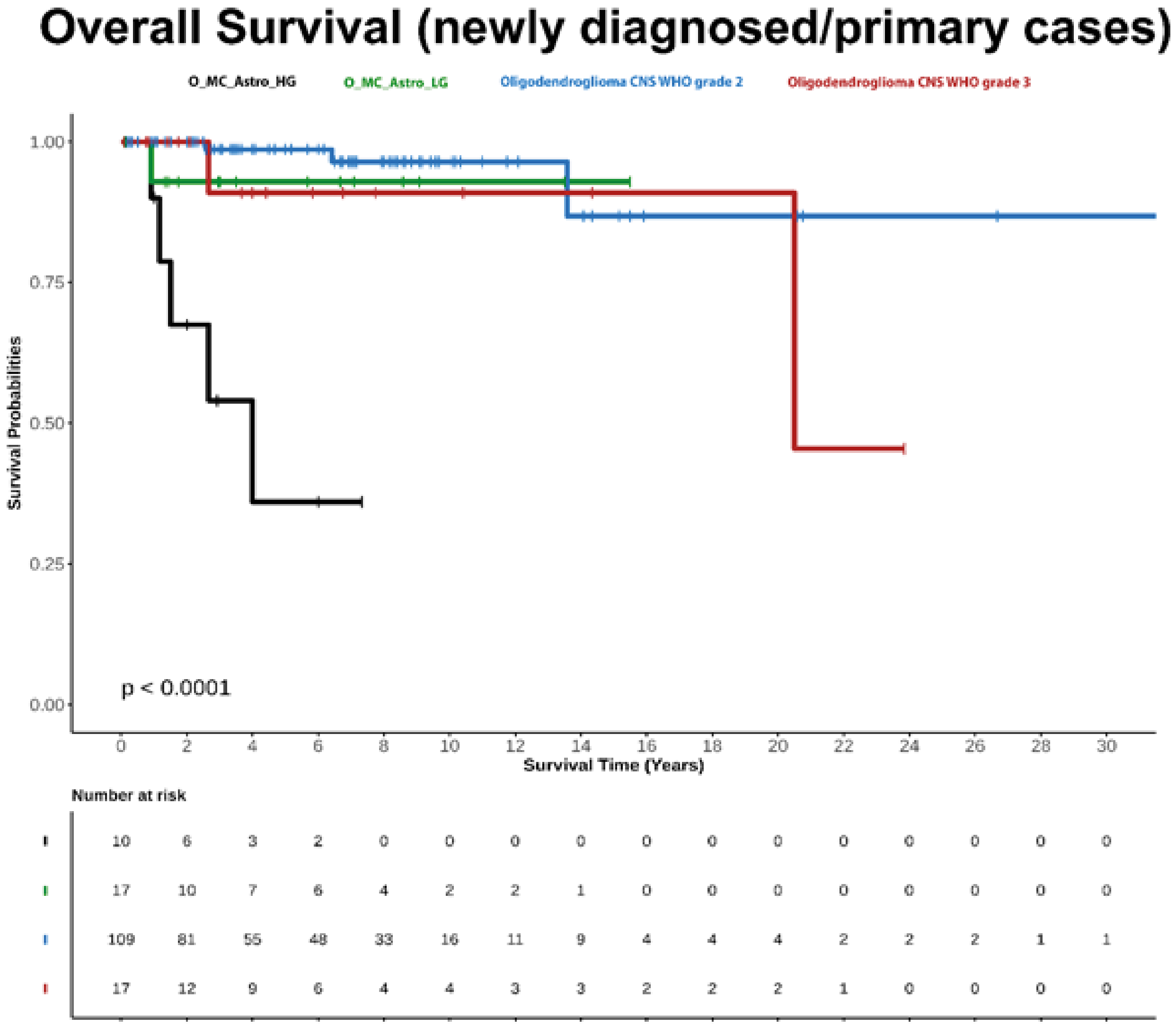

Supplementary Fig. 2: Overall survival of patients with newly diagnosed oligodendrogliomas, IDH-mutant and 1p/19q-codeleted stratified according to MC assignment. Kaplan–Meier analysis shows significantly lower OS in the O_MC_Astro_HG patients in comparison to patients with oligodendrogliomas with other methylation profiles (log-rank p-values: O_MC_Astro_LG vs. MC Oligodendroglioma CNS WHO grade 2: p=0.3516, O_MC_Astro_LG vs Oligodendroglioma, CNS WHO grade 3: p=0.9226, O_MC_Astro_LG vs. O_MC_Astro_HG: p=0.0265 O_MC_Astro_HG vs. Oligodendroglioma, CNS WHO grade 2: p<0.0001, O_MC_Astro_HG vs. Oligodendroglioma CNS, WHO grade 3: p=0.0084). Abbr.: O_MC_Astro_HG, IDH-mutant and 1p/19q-codeleted oligodendrogliomas assigned to the MC astrocytoma, IDH-mutant, high grade; O_MC_Astro_LG, IDH-mutant and 1p/19q-codeleted oligodendrogliomas assigned the MC astrocytoma, IDH-mutant, lower grade.

Supplementary Figure 3

Progression Free Survival

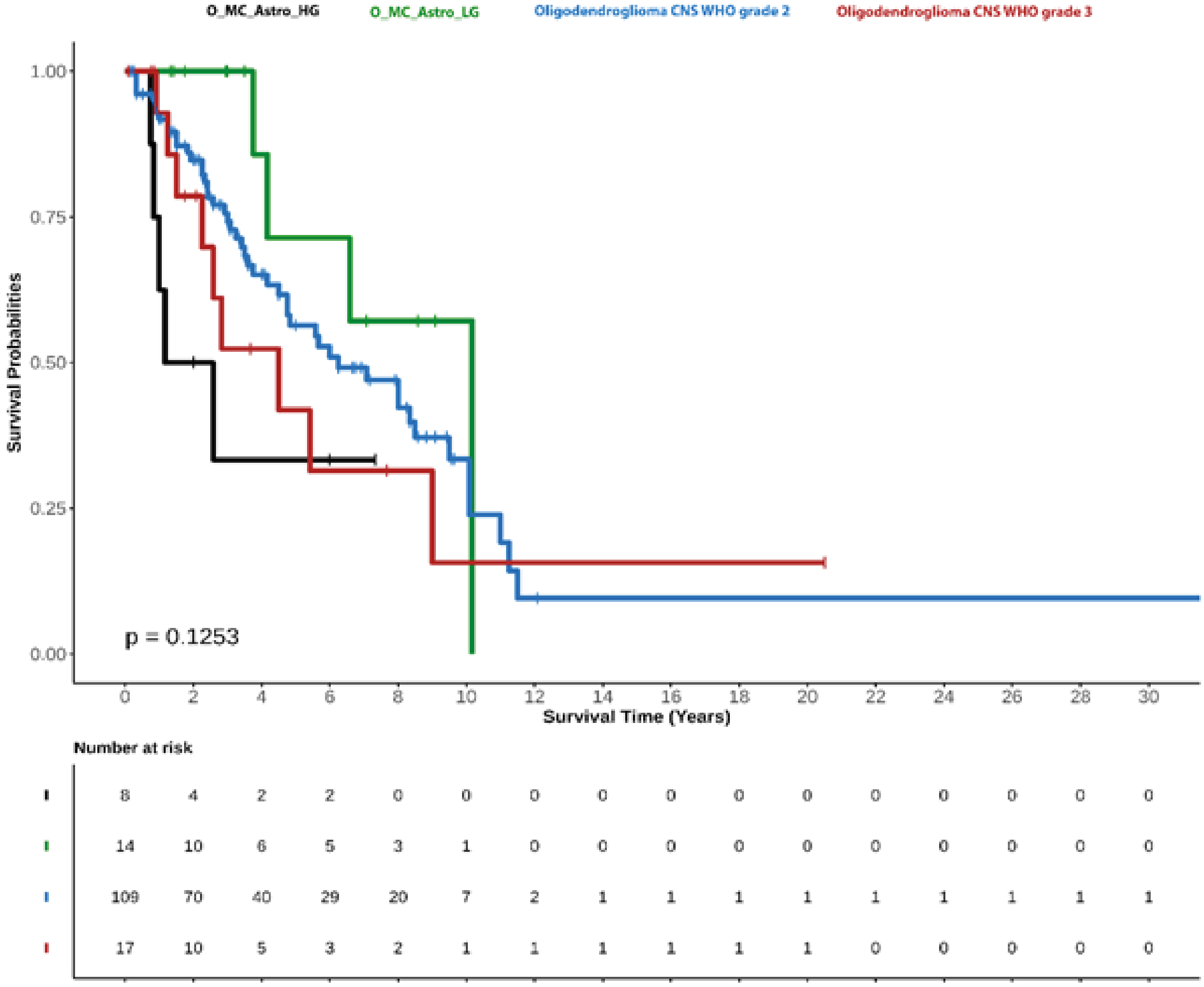

Supplementary Figure 3: Progression Free Survival of patients with oligodendrogliomas, IDH-mutant and 1p/19q-codeleted stratified according to MC assignment. No significant differences in PFS were found between patients with O_MC_Astro_HG tumours versus patients with oligodendrogliomas, IDH-mutant and 1p/19q-codeleted, CNS WHO grade 2 and 3 exhibiting an oligodendroglioma methylation profile (log-rank p-values: O_MC_Astro_LG vs Oligodendroglioma CNS WHO grade 2: p=0.1027, O_MC_Astro_LG vs. Oligodendroglioma, IDH-mutant and 1p/19q-codeleted, CNS WHO grade 3: p=0.0532, O_MC_Astro_LG vs. O_MC_Astro_HG: p=0.0207, O_MC_Astro_HG vs. Oligodendroglioma, IDH-mutant and 1p/19q-codeleted, CNS WHO grade 2: p=0.0815, O_MC_Astro_HG vs Oligodendroglioma, IDH-mutant and 1p/19q-codeleted, CNS WHO grade 3: p=0.4550). **Abbr.:** O_MC_Astro_HG, oligodendroglioma, IDH-mutant and 1p/19q-codeleted, assigned to the MC astrocytoma, IDH-mutant, high grade; O_MC_Astro_LG, oligodendroglioma, IDH-mutant and 1p/19q-codeleted, assigned to the MC astrocytoma, IDH-mutant, lower grade

Supplementary Table 1

Case ID	group	IDH mutation	sex	age at initial diagnosis	location	max follow up [months]	pfs [months]	progressive disease [no=0; yes=1]	alive [yes=0; no=1]	secondary malignisation [0=no; 1=yes]	manifestation [primary tumour=0; reccurence=1]	CNS WHO grade	alkylating therapy before EPIC [1=yes; 0=no]	CDKN2A/B [1 = homozygous deletion]	other relevant CNV besides 1p/19q	pMGMT	max_super_family_class v12.8	max_super_family_score v12.8	max_family_class v12.8	max family score v12.8	max class v12.8	max class score v12.8
LG-1	O_A_LG	IDH1 R132H	f	19	frontoparietal left	16	16	0	0	0	0	2	0	0	-	methylated	Adult-type diffuse gliomas	0,97408266	diffuse glioma, IDH mutant	0,97282569	diffuse glioma, IDH-mutant and 1p19q retained [astroglial type]	0,52289142
LG-2	O_A_LG	IDH1 R132H	m	52	temporal left	72	14	1	0	0	1	2	1	0	-	unmethylated	Adult-type diffuse gliomas	0,71382933	diffuse glioma, IDH mutant	0,69526933	diffuse glioma, IDH-mutant and 1p19q retained [astroglial type]	0,36622111
LG-3	O_A_LG	IDH1 R132H	m	16	frontal left	21	21	0	0	0	0	2	0	0	-	methylated	Adult-type diffuse gliomas	0,88026368	diffuse glioma, IDH mutant	0,87446115	diffuse glioma, IDH-mutant and 1p19q retained [astroglial type]	0,8031133
LG-4	O_A_LG	IDH1 R132H	f	53	bifrontal left, gliomatosis pattern	80	79	1	0	NA	0	3	0	0	-	methylated	Adult-type diffuse gliomas	0,93372505	diffuse glioma, IDH mutant	0,92800077	diffuse glioma, IDH-mutant and 1p19q retained [astroglial type]	0,68191923
LG-5	O_A_LG	IDH1 R132H	m	31	bilocular-basal ganglia left; paraoccipital left	31	60	1	0	0	1	2	0	0	-	methylated	Adult-type diffuse gliomas	0,91501558	diffuse glioma, IDH mutant	0,91001711	diffuse glioma, IDH-mutant and 1p19q retained [astroglial type]	0,85636706
LG-6	O_A_LG	IDH1 R132H	m	27	parietal right	42	42	0	0	0	0	2	0	0	-	methylated	Adult-type diffuse gliomas	0,80976493	diffuse glioma, IDH mutant	0,79799439	diffuse glioma, IDH-mutant and 1p19q retained [astroglial type]	0,43467037
LG-7	O_A_LG	IDH1 R132H	f	37	frontal left	11	11	NA	1	NA	0	2	0	0	-	methylated	Adult-type diffuse gliomas	0,47382267	diffuse glioma, IDH mutant	0,43823005	diffuse glioma, IDH-mutant and 1p19q retained [astroglial type]	0,25767485
LG-8	O_A_LG	IDH1 R132H	m	28	parietal right	36	36	0	0	0	0	2	0	0	-	methylated	Adult-type diffuse gliomas	0,99249914	diffuse glioma, IDH mutant	0,9920917	diffuse glioma, IDH-mutant and 1p19q retained [astroglial type]	0,90488181
LG-9	O_A_LG	IDH1 R132H	f	26	frontal left	17	17	0	0	0	0	2	0	0	-	methylated	Adult-type diffuse gliomas	0,50464103	diffuse glioma, IDH mutant	0,47352703	diffuse glioma, IDH-mutant and 1p19q retained [astroglial type]	0,33424751
LG-10	O_A_LG	IDH1 R132H	m	37	frontal right	3	3	0	0	0	0	2	0	0	-	methylated	Adult-type diffuse gliomas	0,84158591	diffuse glioma, IDH mutant	0,83252504	diffuse glioma, IDH-mutant and 1p19q retained [astroglial type]	0,51714143
LG-11	O_A_LG	IDH1 R132H	m	18	frontal left	109	109	0	0	0	0	2	0	0	-	methylated	Adult-type diffuse gliomas	0,98317443	diffuse glioma, IDH mutant	0,98243265	diffuse glioma, IDH-mutant and 1p19q retained [astroglial type]	0,80447289
LG-12	O_A_LG	IDH1 R132H	m	17	frontal right	68	45	1	0	0	0	2	0	0	-	not determinable	Adult-type diffuse gliomas	0,98225586	diffuse glioma, IDH mutant	0,98147667	diffuse glioma, IDH-mutant and 1p19q retained [astroglial type]	0,73209096

LG-13	O_A_LG	IDH1 R132H	f	19	frontal left	103	103	0	0	0	0	2	0	0	-	not determinable	Adult-type diffuse gliomas	0,38113754	diffuse glioma, IDH mutant	0,35306081	diffuse glioma, IDH-mutant and 1p19q retained [astroglial type]	0,26035604
LG-14	O_A_LG	IDH1 R132H	m	38	temporal left	36	35.5	0	0	0	0	2	0	0	-	methylated	Adult-type diffuse gliomas	0,95957384	diffuse glioma, IDH mutant	0,95724105	diffuse glioma, IDH-mutant and 1p19q retained [astroglial type]	0,52356601
LG-15	O_A_LG	IDH1 R132H	f	64	frontal right	2	NA	NA	0	0	0	2	0	0	-	methylated	Adult-type diffuse gliomas	0,95685364	diffuse glioma, IDH mutant	0,95489002	diffuse glioma, IDH-mutant and 1p19q retained [astroglial type]	0,78314884
LG-16	O_A_LG	IDH2 R172K	m	46	temporal left	162	122	1	NA	NA	0	2	0	0	-	not determinable	Adult-type diffuse gliomas	0,9277343	diffuse glioma, IDH mutant	0,92399392	diffuse glioma, IDH-mutant and 1p19q retained [astroglial type]	0,50002117
LG-17	O_A_LG	IDH1 R132H	m	31	temporal	10	10	NA	0	NA	0	NA	0	0	-	methylated	Adult-type diffuse gliomas	0,91251245	diffuse glioma, IDH mutant	0,90711059	diffuse glioma, IDH-mutant and 1p19q retained [astroglial type]	0,4485598
LG-18	O_A_LG	IDH1 R132H	f	16	frontal right	186	50	1	0	0	0	2	0	0	-	not determinable	Adult-type diffuse gliomas	0,90896662	diffuse glioma, IDH mutant	0,90471455	diffuse glioma, IDH-mutant and 1p19q retained [astroglial type]	0,76792628
LG-19	O_A_LG	IDH1 R132H	m	28	fontal left	85	85	0	0	0	0	2	0	0	-	methylated	Adult-type diffuse gliomas	0,99	diffuse glioma, IDH mutant	0,99	diffuse glioma, IDH-mutant and 1p19q retained [astroglial type]	0,95
LG-20	O_A_LG	IDH1 R132H	m	27	frontal right	NA	NA	NA	NA	NA	0	3	0	0	-	methylated	Adult-type diffuse gliomas	0,87532854	diffuse glioma, IDH mutant	0,85434404	diffuse glioma, IDH-mutant and 1p19q retained [astroglial type]	0,50153505
LG-21	O_A_LG	unknown	m	NA	NA	NA	NA	NA	NA	NA	NA	NA	NA	0	-	methylated	Adult-type diffuse gliomas	0,95682972	diffuse glioma, IDH mutant	0,95502482	diffuse glioma, IDH-mutant and 1p19q retained [astroglial type]	0,60168893
LG-22	O_A_LG	unknown	m	NA	NA	NA	NA	NA	NA	NA	NA	NA	NA	0	-	methylated	Adult-type diffuse gliomas	0,43106156	diffuse glioma, IDH mutant	0,41084296	diffuse glioma, IDH-mutant and 1p19q retained [astroglial type]	0,24055476
LG-23	O_A_LG	unknown	f	NA	NA	NA	NA	NA	NA	NA	NA	NA	NA	0	-	methylated	Adult-type diffuse gliomas	0,62561121	diffuse glioma, IDH mutant	0,60835121	diffuse glioma, IDH-mutant and 1p19q retained [astroglial type]	0,47016076
LG-24	O_A_LG	unknown	m	NA	NA	NA	NA	NA	NA	NA	NA	NA	NA	0	-	methylated	Adult-type diffuse gliomas	0,77388278	diffuse glioma, IDH mutant	0,76226028	diffuse glioma, IDH-mutant and 1p19q retained [astroglial type]	0,41831843
LG-25	O_A_LG	unknown	f	NA	NA	NA	NA	NA	NA	NA	NA	NA	NA	0	-	methylated	Adult-type diffuse gliomas	0,97661253	diffuse glioma, IDH mutant	0,97559367	diffuse glioma, IDH-mutant and 1p19q retained [astroglial type]	0,92840985

LG-26	O_A_LG	unknown	f	NA	left frontal	NA	NA	NA	NA	NA	NA	NA	NA	0	-	methylated	Adult-type diffuse gliomas	0,58491117	diffuse glioma, IDH mutant	0,53675609	diffuse glioma, IDH-mutant and 1p19q retained [astroglial type]	0,26889292
LG-27	O_A_LG	unknown	m	NA	NA	NA	NA	NA	NA	NA	NA	NA	NA	0	-	methylated	Adult-type diffuse gliomas	0,84777842	diffuse glioma, IDH mutant	0,83843057	diffuse glioma, IDH-mutant and 1p19q retained [astroglial type]	0,46102175
HG-1	O_A_HG	IDH1 R132H	m	64	parietooccipital right	3	3	1	0	NA	1	3	1	0	-	methylated	Adult-type diffuse gliomas	0,99	diffuse glioma, IDH mutant	0,99	diffuse glioma, IDH-mutant and 1p19q retained [astroglial type], high grade	0,64
HG-2	O_A_HG	IDH1 R132H	m	33	frontotemporal right	19	19	1	1	1	1	3	1	0	CDK4 amplification	methylated	Adult-type diffuse gliomas	0,99749742	diffuse glioma, IDH mutant	0,99721755	diffuse glioma, IDH-mutant and 1p19q retained [astroglial type], high grade	0,8712927
HG-3	O_A_HG	IDH1 R132H	f	27	temporal left	16	16	1	1	1	1	3	NA	0	0	methylated	Adult-type diffuse gliomas	0,98671519	diffuse glioma, IDH mutant	0,93021967	diffuse glioma, IDH-mutant and 1p19q retained [astroglial type], high grade	0,86501051
HG-4	O_A_HG	unknown	f	61	parietooccipital	215	186	1	0	0	1	3	1	0	0	methylated	Adult-type diffuse gliomas	0,98872862	diffuse glioma, IDH mutant	0,98531382	diffuse glioma, IDH-mutant and 1p19q retained [astroglial type], high grade	0,97460986
HG-5	O_A_HG	IDH1 R132H	m	34	frontal left	159	95	1	1	1	1	2	1	0	-	methylated	Adult-type diffuse gliomas	0,99792724	diffuse glioma, IDH mutant	0,9971047	diffuse glioma, IDH-mutant and 1p19q retained [astroglial type], high grade	0,98826153
HG-6	O_A_HG	IDH1 R132H	m	48	frontal right	11	10	1	1	1	0	3	0	0	MDM4 and CDK4 amplification	methylated	Adult-type diffuse gliomas	0,97757065	diffuse glioma, IDH mutant	0,96746557	diffuse glioma, IDH-mutant and 1p19q retained [astroglial type], high grade	0,94149483
HG-7	O_A_HG	IDH1 R132H	f	57	temporal	88	88	0	0	0	0	3	0	0	-	methylated	Adult-type diffuse gliomas	0,99967683	diffuse glioma, IDH mutant	0,99952854	diffuse glioma, IDH-mutant and 1p19q retained [astroglial type], high grade	0,99714735
HG-8	O_A_HG	IDH1 R132H	m	38	postzentral right	12	12	1	0	NA	1	3	NA	0	-	methylated	Adult-type diffuse gliomas	0,99805231	diffuse glioma, IDH mutant	0,9977241	diffuse glioma, IDH-mutant and 1p19q retained [astroglial type], high grade	0,99378893
HG-9	O_A_HG	IDH1 R132H	m	67	frontal right	14	14	1	1	0	0	3	0	0	-	methylated	Adult-type diffuse gliomas	0,9462622	diffuse glioma, IDH mutant	0,88530596	diffuse glioma, IDH-mutant and 1p19q retained [astroglial type], high grade	0,51779206
HG-10	O_A_HG	IDH1 R132H	m	44	corpus callosum	48	12	1	1	NA	0	NA	0	1	-	methylated	Adult-type diffuse gliomas	0,99927874	diffuse glioma, IDH mutant	0,99902188	diffuse glioma, IDH-mutant and 1p19q retained [astroglial type], high grade	0,95203962

HG-11	O_A_HG	IDH1 R132H	f	73	frontal right	18	NA	NA	1	NA	0	3	0	1	-	methylated	Adult-type diffuse gliomas	0,98036161	diffuse glioma, IDH mutant	0,95427259	diffuse glioma, IDH-mutant and 1p19q retained [astroglial type], high grade	0,84311985
HG-12	O_A_HG	IDH1 R132H	f	77	frontal right	32	31	1	1	0	0	3	0	1	-	not determinable	Adult-type diffuse gliomas	0,9979891	diffuse glioma, IDH mutant	0,99474235	diffuse glioma, IDH-mutant and 1p19q retained [astroglial type], high grade	0,9846876
HG-13	O_A_HG	IDH1 R132H	m	37	frontotemporal right	127	108	1	0	NA	1	3	1	0	-	methylated	Adult-type diffuse gliomas	0,98878374	diffuse glioma, IDH mutant	0,96515546	diffuse glioma, IDH-mutant and 1p19q retained [astroglial type], high grade	0,95236357
HG-14	O_A_HG	IDH1 R132H	f	29	parietal	12	12	NA	0	NA	0	NA	0	1	-	methylated	Adult-type diffuse gliomas	0,99829396	diffuse glioma, IDH mutant	0,99776423	diffuse glioma, IDH-mutant and 1p19q retained [astroglial type], high grade	0,98042155
HG-15	O_A_HG	IDH1 R132H	m	41	temporal	72	72	0	0	0	0	NA	0	0	-	methylated	Adult-type diffuse gliomas	0,99582519	diffuse glioma, IDH mutant	0,99521255	diffuse glioma, IDH-mutant and 1p19q retained [astroglial type], high grade	0,69126162
HG-16	O_A_HG	IDH1 R132H	f	37	temporal	24	24	0	0	0	0	NA	0	1	PDGFRA amplification	methylated	Adult-type diffuse gliomas	0,99891732	diffuse glioma, IDH mutant	0,99881441	diffuse glioma, IDH-mutant and 1p19q retained [astroglial type], high grade	0,97064517
HG-17	O_A_HG	IDH1 R132H	f	52	frontal right	5	5	1	0	0	1	3	1	0	-	methylated	Adult-type diffuse gliomas	0,99445117	diffuse glioma, IDH mutant	0,99370423	diffuse glioma, IDH-mutant and 1p19q retained [astroglial type], high grade	0,73127256
HG-18	O_A_HG	IDH1 R132H	m	42	temporal right	19	19	1	1	1	1	3	NA	0	-	methylated	Adult-type diffuse gliomas	0,96021803	diffuse glioma, IDH mutant	0,95357132	diffuse glioma, IDH-mutant and 1p19q retained [astroglial type], high grade	0,76629514
HG-19	O_A_HG	IDH1 R132H	m	30	bifrontal	153	65	1	0	0	1	2	1	0	-	methylated	Adult-type diffuse gliomas	0,93648832	diffuse glioma, IDH mutant	0,93016419	diffuse glioma, IDH-mutant and 1p19q retained [astroglial type], high grade	0,3587192
HG-20	O_A_HG	IDH1 R132H	m	40	frontal right	35	9	1	0	NA	0	3	0	0	-	methylated	Adult-type diffuse gliomas	0,99	diffuse glioma, IDH mutant	0,99	diffuse glioma, IDH-mutant and 1p19q retained [astroglial type], high grade	0,99
HG-21	O_A_HG	unknown	m	47	parietal right	5	5	1	0	1	1	3	NA	1	-	methylated	Adult-type diffuse gliomas	0,99980441	diffuse glioma, IDH mutant	0,999774	diffuse glioma, IDH-mutant and 1p19q retained [astroglial type], high grade	0,99824827
HG-22	O_A_HG	IDH1 R132H	f	NA	frontal left	NA	NA	NA	NA	NA	NA	NA	NA	0	-	methylated	Adult-type diffuse gliomas	0,99873362	diffuse glioma, IDH mutant	0,99841814	diffuse glioma, IDH-mutant and 1p19q retained [astroglial type], high grade	0,99278791

HG-23	O_A_HG	IDH1 R132H	m	NA	NA	NA	NA	NA	NA	NA	0	3	0	0	-	methylated	Adult-type diffuse gliomas	0,99988365	diffuse glioma, IDH mutant	0,99986775	diffuse glioma, IDH-mutant and 1p19q retained [astroglial type], high grade	0,99950106
HG-24	O_A_HG	IDH1 R132H	m	NA	left frontal	NA	NA	NA	NA	NA	NA	3	NA	0	PTEN homozygous deletion	methylated	Adult Type Diffuse Gliomas	0,98	diffuse glioma, IDH mutant	0,98	diffuse glioma, IDH-mutant and 1p19q retained [astroglial type], high grade	0.53
HG-25	O_A_HG	unknown	m	NA	NA	NA	NA	NA	NA	NA	NA	NA	NA	0	-	methylated	Adult-type diffuse gliomas	0,86773894	diffuse glioma, IDH mutant	0,85938951	diffuse glioma, IDH-mutant and 1p19q retained [astroglial type], high grade	0,49279091
HG-26	O_A_HG	unknown	m	NA	NA	NA	NA	NA	NA	NA	0	3	0	1	-	methylated	Adult-type diffuse gliomas	0,99988365	diffuse glioma, IDH mutant	0,99986775	diffuse glioma, IDH-mutant and 1p19q retained [astroglial type], high grade	0,99950106
HG-27	O_A_HG	unknown	m	NA	NA	NA	NA	NA	NA	NA	NA	NA	NA	0	-	methylated	Adult-type diffuse gliomas	0,98972693	diffuse glioma, IDH mutant	0,93740333	diffuse glioma, IDH-mutant and 1p19q retained [astroglial type], high grade	0,92260236
HG-28	O_A_HG	unknown	m	NA	NA	NA	NA	NA	NA	NA	NA	NA	NA	1	CDK4 gain	methylated	Adult-type diffuse gliomas	0,98794075	diffuse glioma, IDH mutant	0,98543206	diffuse glioma, IDH-mutant and 1p19q retained [astroglial type], high grade	0,98116603
HG-29	O_A_HG	unknown	m	NA	NA	NA	NA	NA	NA	NA	NA	NA	NA	1	-	methylated	Adult-type diffuse gliomas	0,99858499	diffuse glioma, IDH mutant	0,99839145	diffuse glioma, IDH-mutant and 1p19q retained [astroglial type], high grade	0,96184104
HG-30	O_A_HG	unknown	f	NA	NA	NA	NA	NA	NA	NA	NA	NA	NA	1	-	methylated	Adult-type diffuse gliomas	0,99537265	diffuse glioma, IDH mutant	0,99458843	diffuse glioma, IDH-mutant and 1p19q retained [astroglial type], high grade	0,97824607
HG-31	O_A_HG	unknown	m	NA	NA	NA	NA	NA	NA	NA	NA	NA	NA	0	-	methylated	Adult-type diffuse gliomas	0,95720796	diffuse glioma, IDH mutant	0,9487502	diffuse glioma, IDH-mutant and 1p19q retained [astroglial type], high grade	0,55919936
HG-32	O_A_HG	unknown	m	NA	NA	NA	NA	NA	NA	NA	NA	NA	NA	0	-	methylated	Adult-type diffuse gliomas	0,99265729	diffuse glioma, IDH mutant	0,98724291	diffuse glioma, IDH-mutant and 1p19q retained [astroglial type], high grade	0,91804631
HG-33	O_A_HG	unknown	f	NA	NA	NA	NA	NA	NA	NA	NA	NA	NA	0	CDK4 amplification	methylated	Adult-type diffuse gliomas	0,89232641	diffuse glioma, IDH mutant	0,87458068	diffuse glioma, IDH-mutant and 1p19q retained [astroglial type], high grade	0,69292595
HG-34	O_A_HG	unknown	m	NA	NA	NA	NA	NA	NA	NA	NA	NA	NA	1	-	methylated	Adult-type diffuse gliomas	0,99980441	diffuse glioma, IDH mutant	0,999774	diffuse glioma, IDH-mutant and 1p19q retained [astroglial type], high grade	0,99824827

HG-35	O_A_HG	unknown	m	NA	frontal right	NA	NA	NA	NA	NA	NA	NA	NA	1	-	methylated	Adult-type diffuse gliomas	0,99884095	diffuse glioma, IDH mutant	0,99870343	diffuse glioma, IDH-mutant and 1p19q retained [astroglial type], high grade	0,99423445
HG-36	O_A_HG	unknown	m	NA	NA	NA	NA	NA	NA	NA	NA	NA	NA	0	-	methylated	Adult-type diffuse gliomas	0,99621857	diffuse glioma, IDH mutant	0,99538632	diffuse glioma, IDH-mutant and 1p19q retained [astroglial type], high grade	0,90412748
HG-37	O_A_HG	unknown	f	NA	NA	NA	NA	NA	NA	NA	NA	NA	NA	0	-	methylated	Adult-type diffuse gliomas	0,99817491	diffuse glioma, IDH mutant	0,99680715	diffuse glioma, IDH-mutant and 1p19q retained [astroglial type], high grade	0,98616879
HG-38	O_A_HG	unknown	f	NA	NA	NA	NA	NA	NA	NA	NA	NA	NA	0	-	methylated	Adult-type diffuse gliomas	0,99992775	diffuse glioma, IDH mutant	0,99989514	diffuse glioma, IDH-mutant and 1p19q retained [astroglial type], high grade	0,99974323
HG-39	O_A_HG	unknown	m	NA	NA	NA	NA	NA	NA	NA	NA	NA	NA	1	-	methylated	Adult-type diffuse gliomas	0,9837955	diffuse glioma, IDH mutant	0,9732016	diffuse glioma, IDH-mutant and 1p19q retained [astroglial type], high grade	0,90457155
HG-40	O_A_HG	unknown	m	NA	NA	NA	NA	NA	NA	NA	NA	NA	NA	0	-	methylated	Adult-type diffuse gliomas	0,99570047	diffuse glioma, IDH mutant	0,99409854	diffuse glioma, IDH-mutant and 1p19q retained [astroglial type], high grade	0,88647086
HG-41	O_A_HG	unknown	m	NA	III. ventricle	NA	NA	NA	NA	NA	NA	NA	NA	1	-	methylated	Adult-type diffuse gliomas	0,96029073	diffuse glioma, IDH mutant	0,94015746	diffuse glioma, IDH-mutant and 1p19q retained [astroglial type], high grade	0,84422794
HG-42	O_A_HG	unknown	f	33	parietal right	NA	NA	NA	NA	NA	0	3	0	1	-	methylated	Adult-type diffuse gliomas	0,99097109	diffuse glioma, IDH mutant	0,98844899	diffuse glioma, IDH-mutant and 1p19q retained [astroglial type], high grade	0,98112548

Supplementary Table 2

Score for	IDH1	pTERT	CIC	CDKN2C	PIK3CA	FUBP1	SETD2	TP53	KRAS	PIK3R1
A_IDH_HG	NM_005896.4:c.395G>A | p.Arg132His | chr2:g.208248388C>T	NM_198253.3:c.-124C>T | chr5:g.1295113G>A	-	-	-	NM_003902.5:c.262C>T | p.Gln88* | chr1:g.77967655G>A	NM_014159.7:c.7266G>A | p.Trp2422* | chr3:g.47037750C>T	NM_000546.6:c.438G>A | p.Trp146* | chr17:g.7675174C>T	-	-
A_IDH	NM_005896.4:c.395G>A | p.Arg132His | chr2:g.208248388C>T	-	NM_001386298.1:c.6092_6093delTG | p.Val2031fs | chr19:g.42292755_42292756del	-	-	-	-	-	-	-
A_IDH_HG	NM_005896.4:c.395G>A | p.Arg132His | chr2:g.208248388C>T	NM_198253.3:c.-124C>T | chr5:g.1295113G>A	NM_001386298.1:c.3331C>T | p.Arg1111Trp | chr19:g.42287566C>T	-	-	-	-	-	-	-
A_IDH	NM_005896.4:c.395G>A | p.Arg132His | chr2:g.208248388C>T	-	NM_001386298.1:c.3328C>T | p.Arg1110Trp | chr19:g.42287563C>T	-	-	-	-	-	-	-
A_IDH_HG	NM_005896.4:c.395G>A | p.Arg132His | chr2:g.208248388C>T	NM_198253.3:c.-146C>T | chr5:g.1295135G>A	NM_001386298.1:c.3337A>G | p.Met1113Val | chr19:g.42287572A>G (VUS)	-	-	NM_003902.5:c.601delA | p.Ile201fs | chr1:g.77965104del	-	-	-	-
A_IDH_HG	NM_005896.4:c.395G>A | p.Arg132His | chr2:g.208248388C>T	NM_198253.3:c.-124C>T | chr5:g.1295113G>A	M_001386298.1:c.5932C>T | p.Gln1978* | chr19:g.42292595C>T	-	-	-	-	-	-	-
A_IDH_HG	NM_005896.4:c.395G>A | p.Arg132His | chr2:g.208248388C>T	NM_198253.3:c.-124C>T | chr5:g.1295113G>A	-	-	-	-	-	-	NM_033360.4:c.35G>C | p.Gly12Ala | chr12:g.25245350C>G	NM_181523.3:c.1690A>G | p.Asn564Asp | chr5:g.68295269A>G
A_IDH_HG	NM_005896.4:c.395G>A | p.Arg132His | chr2:g.208248388C>T	NM_198253.3:c.-124C>T | chr5:g.1295113G>A	NM_001386298.1:c.4301G>A | p.Gly1434Asp | chr19:g.42290342G>A (VUS)	-	-	-	-	-	-	-
A_IDH_HG	NM_005896.4:c.395G>A | p.Arg132His | chr2:g.208248388C>T	NM_198253.3:c.-124C>T | chr5:g.1295113G>A	NM_001386298.1:c.3431G>A | p.Gly1144Asp | chr19:g.42287666G>A	-	-	NM_003902.5:c.121-17_123delTTCTTTTTTTTTTTAGATT	NM_014159.7:c.71+2T>C	-	-	-
A_IDH_HG	NM_005896.4:c.395G>A | p.Arg132His | chr2:g.208248388C>T	NM_198253.3:c.-124C>T | chr5:g.1295113G>A	NM_001386298.1:c.3370C>T | p.Arg1124Trp | chr19:g.42287605C>T	NM_078626.3:c.288dupT | p.Asn97fs | chr1:g.50974051dup	NM_006218.4:c.3140A>G | p.His1047Arg | chr3:g.179234297A>G	-	-	-	-	-
A_IDH	NM_005896.4:c.395G>A | p.Arg132His | chr2:g.208248388C>T	NM_198253.3:c.-146C>T | chr5:g.1295135G>A	NM_001386298.1:c.5094dupG | p.Thr1699fs | chr19:g.42291135dup	-	NM_006218.4:c.3140A>G | p.His1047Arg | chr3:g.179234297A>G	-	-	-	-	-
A_IDH	NM_005896.4:c.395G>A | p.Arg132His | chr2:g.208248388C>T	NM_198253.3:c.-124C>T | chr5:g.1295113G>A	-	-	-	-	-	-	-	-
A_IDH	NM_005896.4:c.395G>A | p.Arg132His | chr2:g.208248388C>T	NM_198253.3:c.-124C>T | chr5:g.1295113G>A	-	-	-	-	-	-	-	-
A_IDH	NM_005896.4:c.395G>A | p.Arg132His | chr2:g.208248388C>T	NM_198253.3:c.-124C>T | chr5:g.1295113G>A	-	-	-	-	-	-	-	-
A_IDH	NM_005896.4:c.395G>A | p.Arg132His | chr2:g.208248388C>T	NM_198253.3:c.-124C>T | chr5:g.1295113G>A	NM_001386298.1:c.3361A>G | p.Lys1121Glu | chr19:g.42287596A>G (VUS)	-	-	-	-	-	-	-
A_IDH_HG	NM_005896.4:c.395G>A | p.Arg132His | chr2:g.208248388C>T	NM_198253.3:c.-146C>T | chr5:g.1295135G>A	NM_001386298.1:c.6092_6093delTG | p.Val2031fs | chr19:g.42292755_42292756del	-	-	-	-	-	-	-

Supplementary Data

HG-01

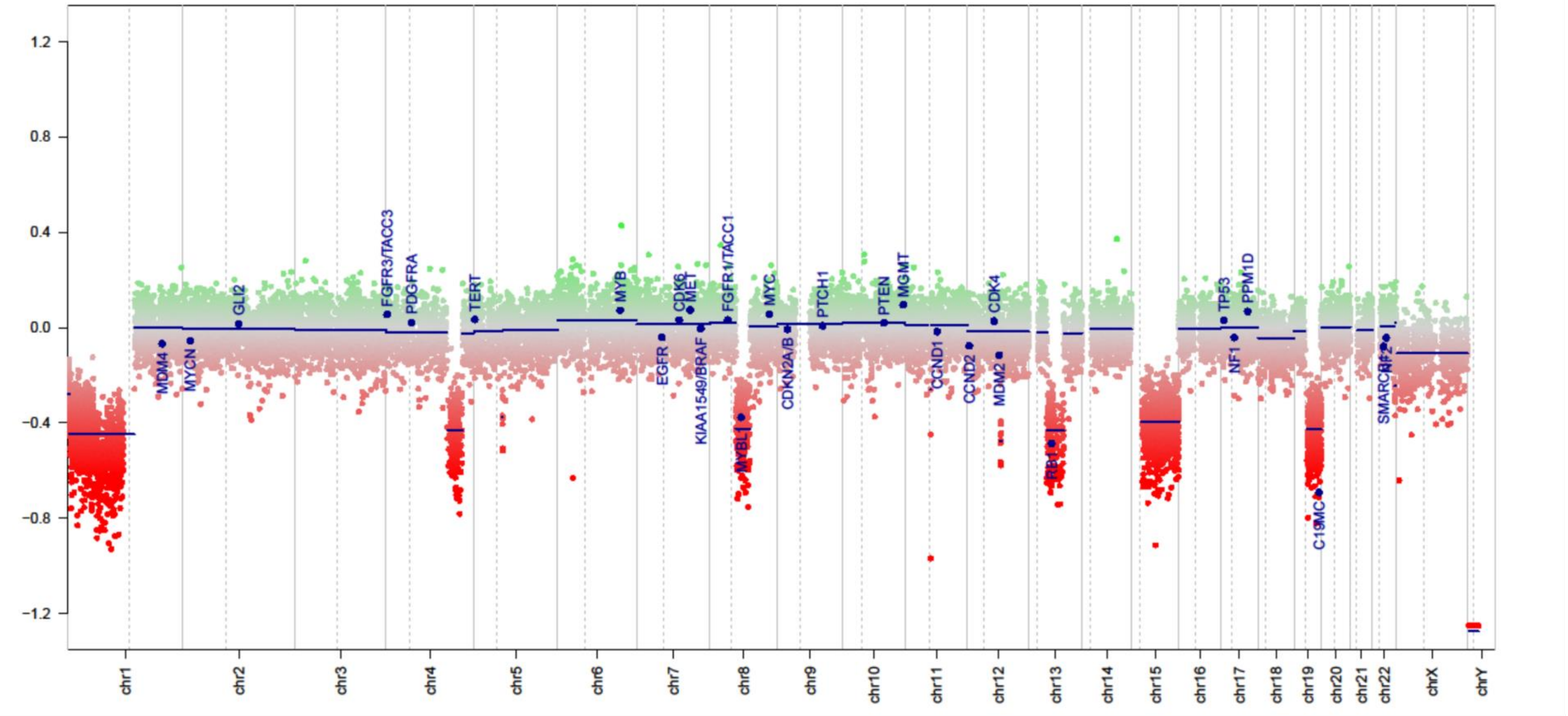
1.2
0.8
0.4
0.0
−0.4
−0.8
−1.2
MDM4
MYCN
GLI2
FGFR3/TACC3
PDGFRA
TERT
MYB
EGFR
CDK6
MET
KIAA1549/BRAF
FGFR1/TACC1
MYBL1
MYC
CDKN2A/B
PTCH1
PTEN
MGMT
CCND1
CCND2
CDK4
MDM2
RB1
TP53
NF1
PPM1D
C19MC
SMARCB1
chr1
chr2
chr3
chr4
chr5
chr6
chr7
chr8
chr9
chr10
chr11
chr12
chr13
chr14
chr15
chr16
chr17
chr18
chr19
chr20
chr21
chr22
chrX
chrY

HG-02

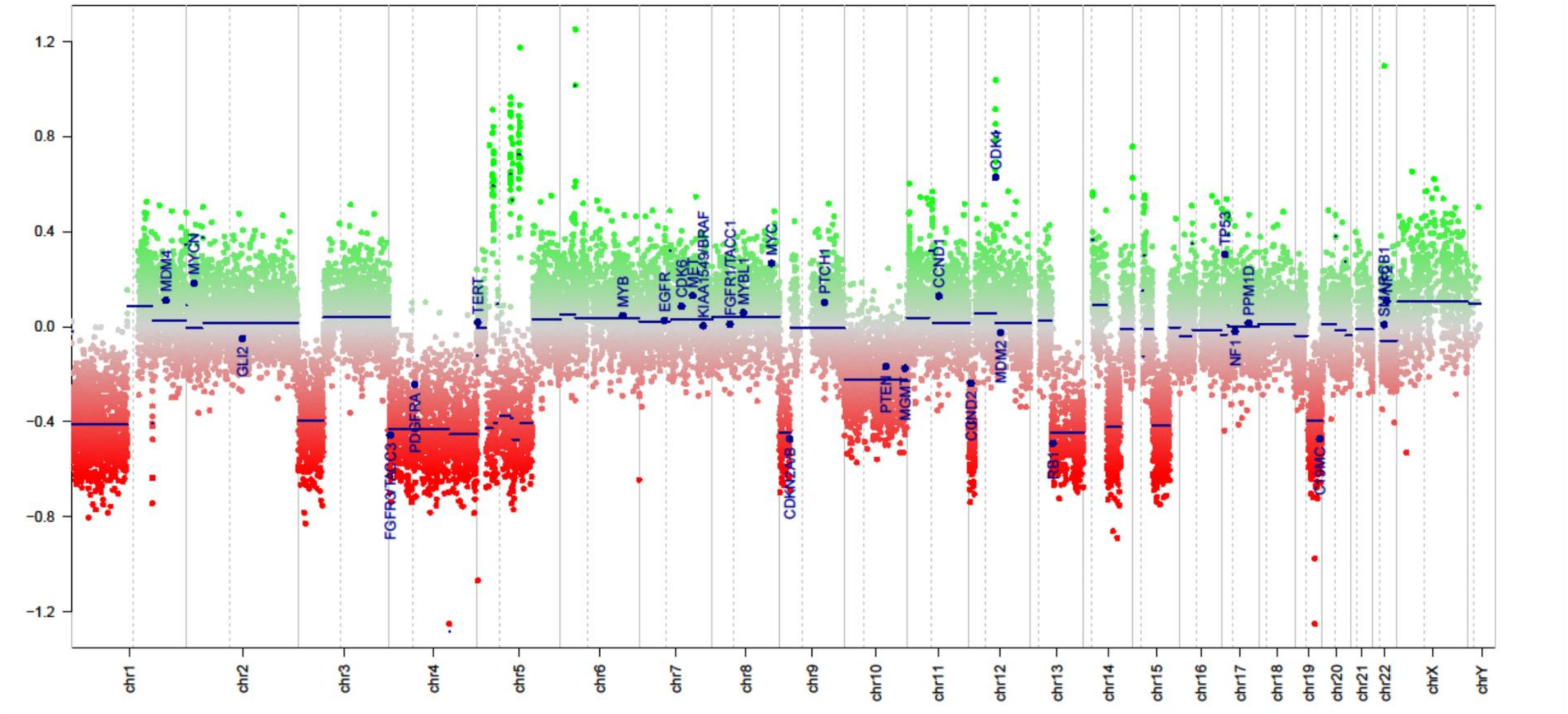

1.2
0.8
0.4
0.0
-0.4
-0.8
-1.2
MDM4
MYCN
GLI2
TERT
FGFR3/TACC3
PDGFRA
MYB
EGFR
CDK6
MET
KIAA1549/BRAF
FGFR1/TACC1
MYBL1
MYC
CDKN2A/B
PTCH1
PTEN
MGMT
CCND1
CCND2
CDK4
MDM2
RB1
TP53
NF1
PPM1D
SMARCB1
chr1
chr2
chr3
chr4
chr5
chr6
chr7
chr8
chr9
chr10
chr11
chr12
chr13
chr14
chr15
chr16
chr17
chr18
chr19
chr20
chr21
chr22
chrX
chrY

HG-03

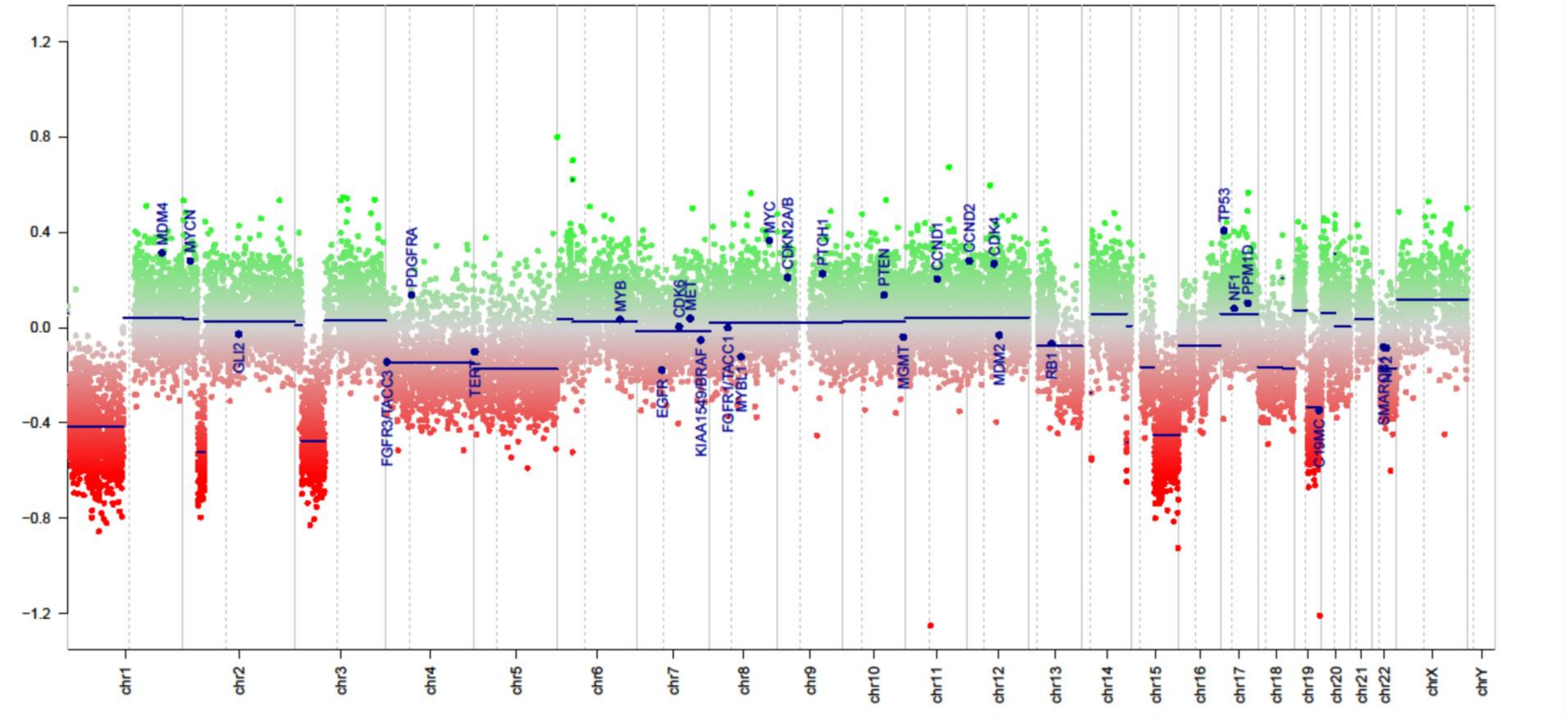
1.2
0.8
0.4
0.0
-0.4
-0.8
-1.2
MDM4
MYCN
GLI2
PDGFRA
FGFR3/TACC3
TERT
MYB
CDK6
MET
EGFR
KIAA1549/BRAF
FGFR1/TACC1
MYBL1
MYC
CDKN2A/B
PTCH1
PTEN
MGMT
CCND1
CCND2
CDK4
MDM2
RB1
TP53
NF1
PPM1D
C19MC
SMARCB1
chr1
chr2
chr3
chr4
chr5
chr6
chr7
chr8
chr9
chr10
chr11
chr12
chr13
chr14
chr15
chr16
chr17
chr18
chr19
chr20
chr21
chr22
chrX
chrY

HG-04

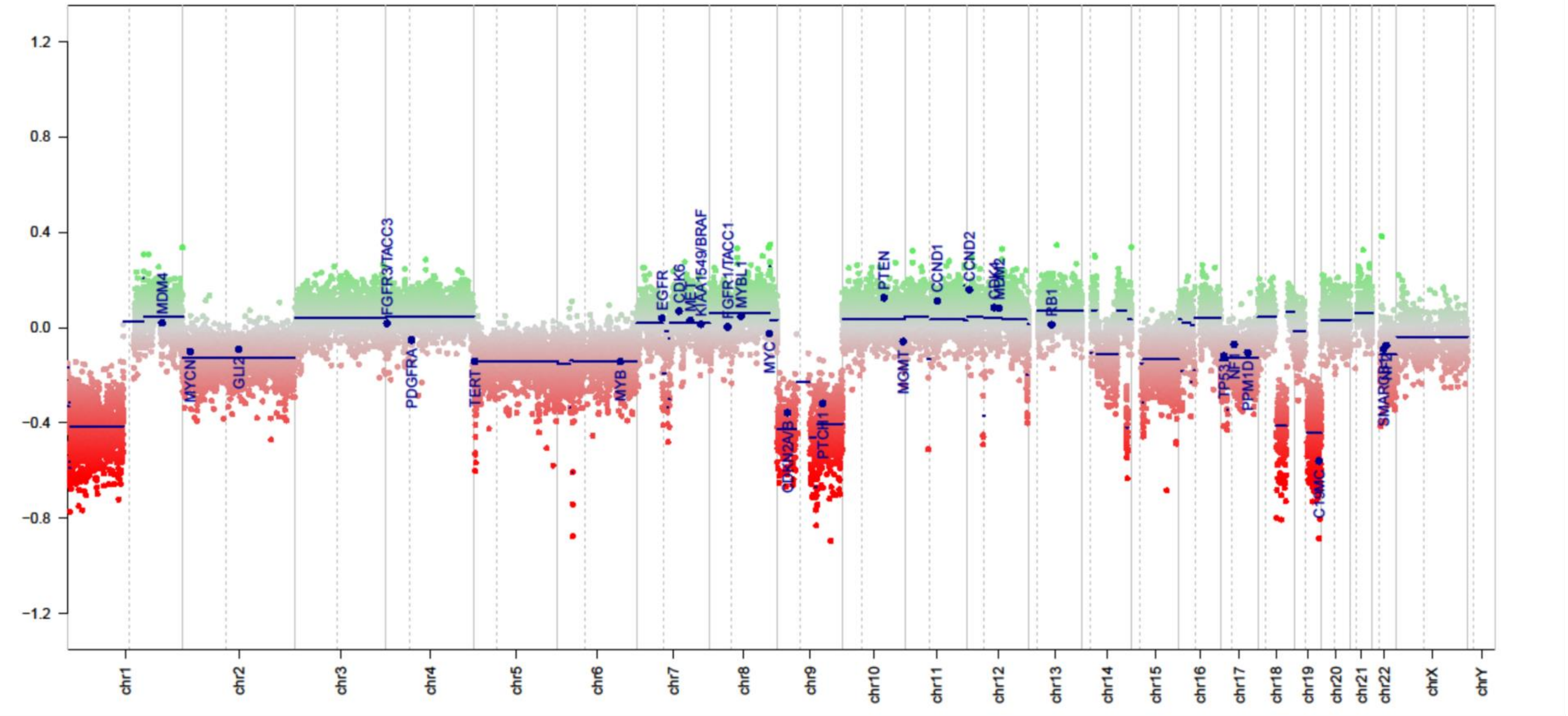

1.2
0.8
0.4
0.0
-0.4
-0.8
-1.2
MDM4
MYCN
GLI2
FGFR3/TACC3
PDGFRA
TERT
MYB
EGFR
CDK6
MET
KIAA1549/BRAF
FGFR1/TACC1
MYBL1
MYC
CDKN2A/B
PTCH1
PTEN
MGMT
CCND1
CCND2
MDM2
RB1
TP53
PPM1D
chr1
chr2
chr3
chr4
chr5
chr6
chr7
chr8
chr9
chr10
chr11
chr12
chr13
chr14
chr15
chr16
chr17
chr18
chr19
chr20
chr21
chr22
chrX
chrY

HG-05

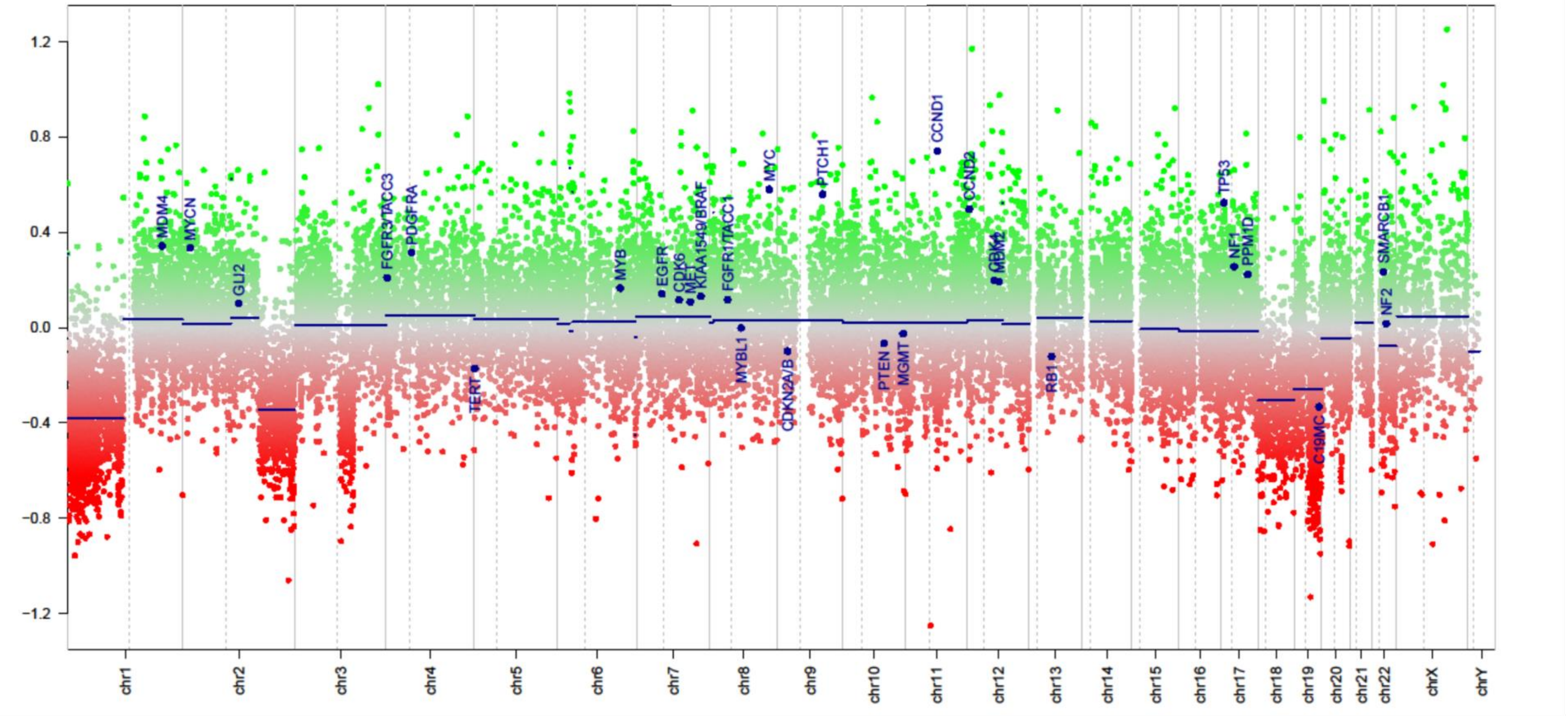

1.2
0.8
0.4
0.0
-0.4
-0.8
-1.2
MDM4
MYCN
GLI2
FGFR3/TACC3
PDGFRA
TERT
MYB
EGFR
CDK6
MET
KIAA1549/BRAF
FGFR1/TACC1
MYBL1
MYC
CDKN2A/B
PTCH1
PTEN
MGMT
CCND1
CCND2
CDK4
MDM2
RB1
TP53
NF1
PPM1D
C19MC
SMARCB1
NF2
chr1
chr2
chr3
chr4
chr5
chr6
chr7
chr8
chr9
chr10
chr11
chr12
chr13
chr14
chr15
chr16
chr17
chr18
chr19
chr20
chr21
chr22
chrX
chrY

HG-06

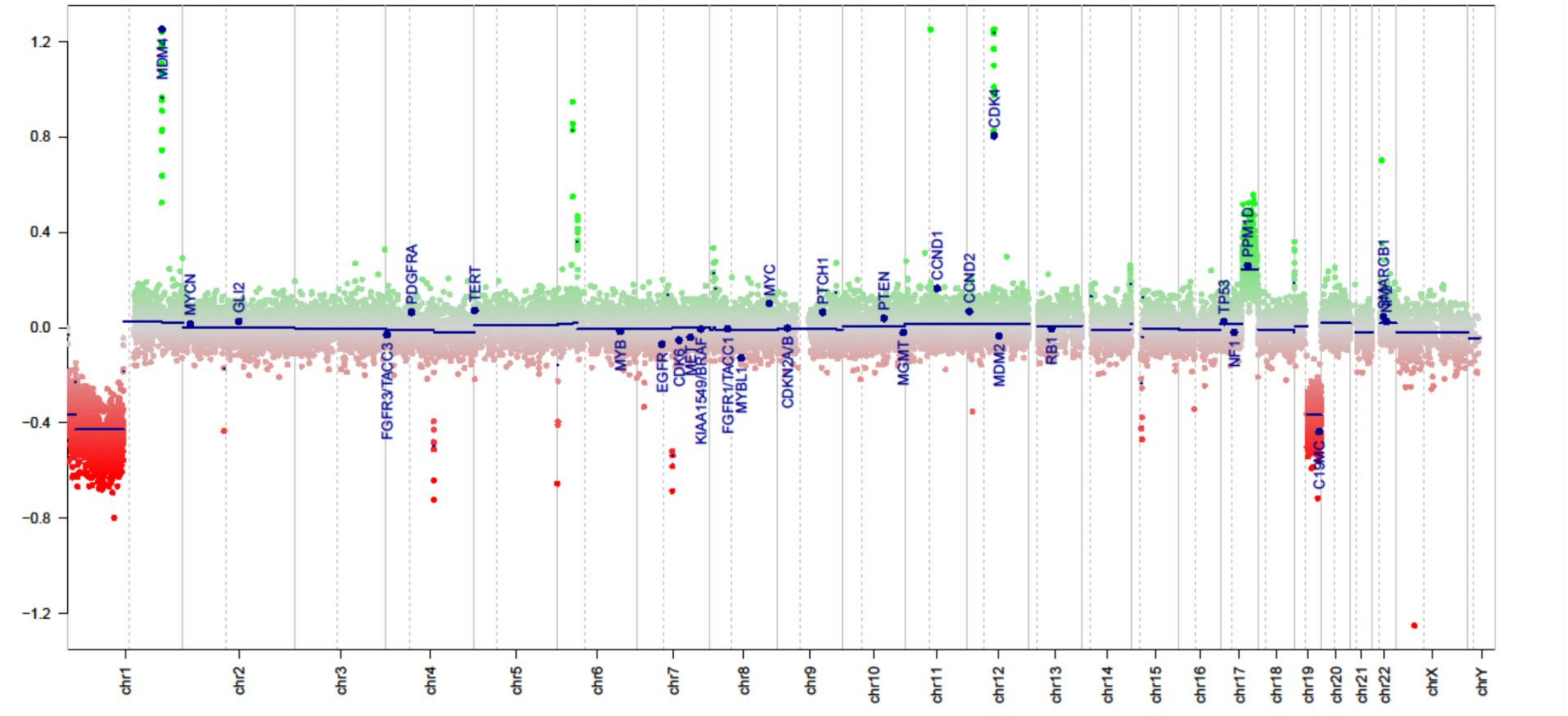

HG-07

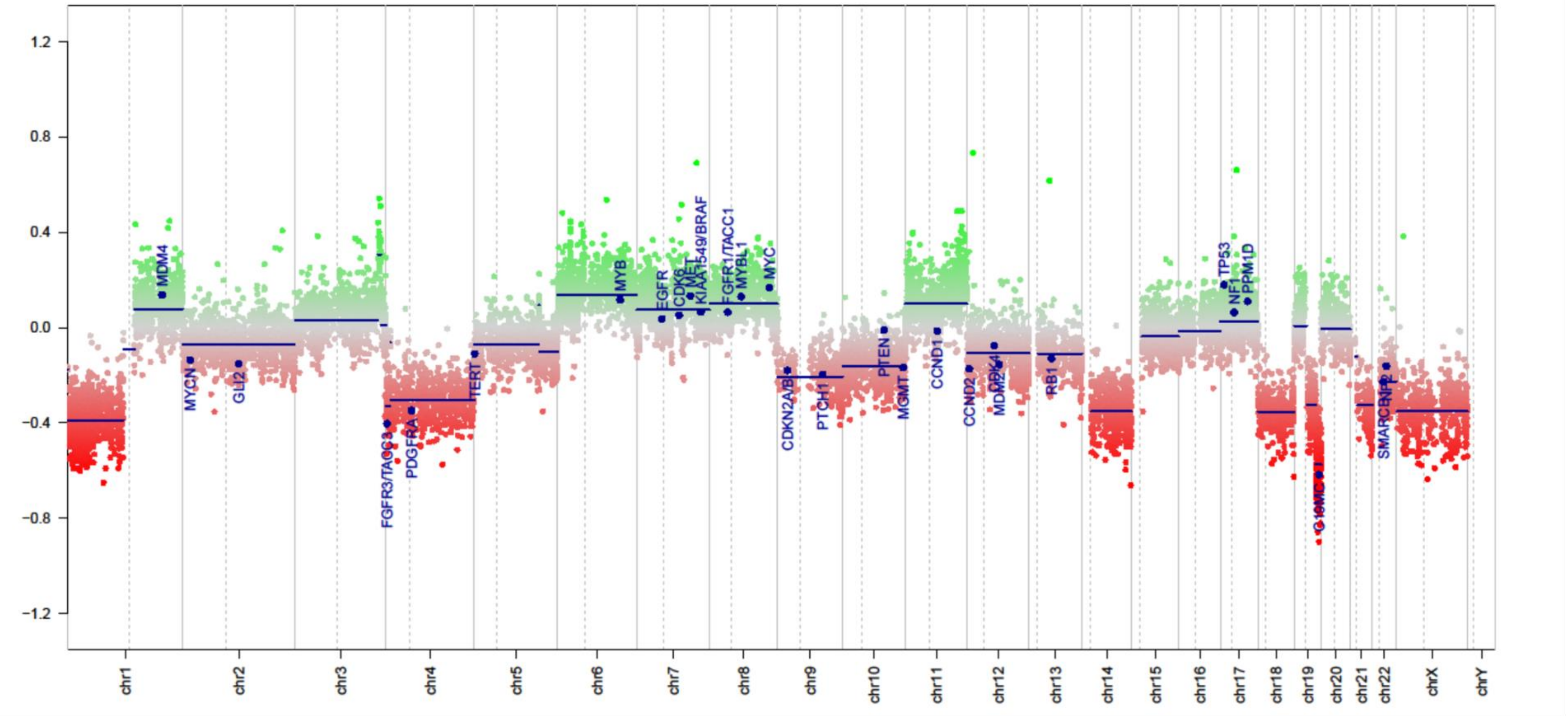

1.2
0.8
0.4
0.0
−0.4
−0.8
−1.2
MDM4
MYCN
GLI2
FGFR3/TACC3
PDGFRA
TERT
MYB
EGFR
CDK6
MET
KIAA1549/BRAF
FGFR1/TACC1
MYBL1
MYC
PTCH1
PTEN
MGMT
CCND1
CCND2
MDM2
RB1
TP53
NF1
PPM1D
chr1
chr2
chr3
chr4
chr5
chr6
chr7
chr8
chr9
chr10
chr11
chr12
chr13
chr14
chr15
chr16
chr17
chr18
chr19
chr20
chr21
chr22
chrX
chrY

HG-08

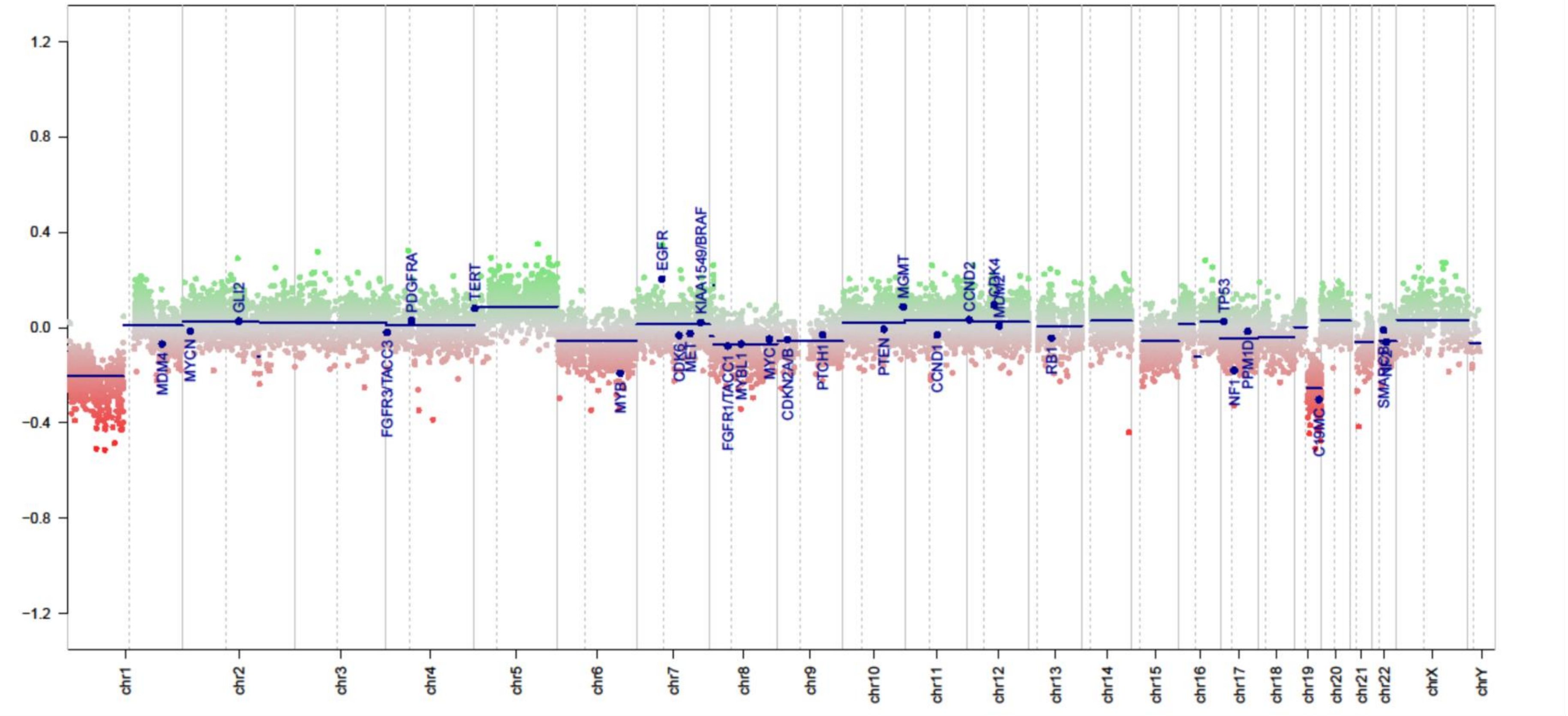

1.2
0.8
0.4
0.0
−0.4
−0.8
−1.2
MDM4
MYCN
GLI2
FGFR3/TACC3
PDGFRA
TERT
MYB
EGFR
CDK6
MET
KIAA1549/BRAF
FGFR1/TACC1
MYBL1
MYC
CDKN2A/B
PTCH1
PTEN
MGMT
CCND1
CCND2
CDK4
MDM2
RB1
TP53
NF1
PPM1D
SMARCB1
chr1
chr2
chr3
chr4
chr5
chr6
chr7
chr8
chr9
chr10
chr11
chr12
chr13
chr14
chr15
chr16
chr17
chr18
chr19
chr20
chr21
chr22
chrX
chrY

HG-09

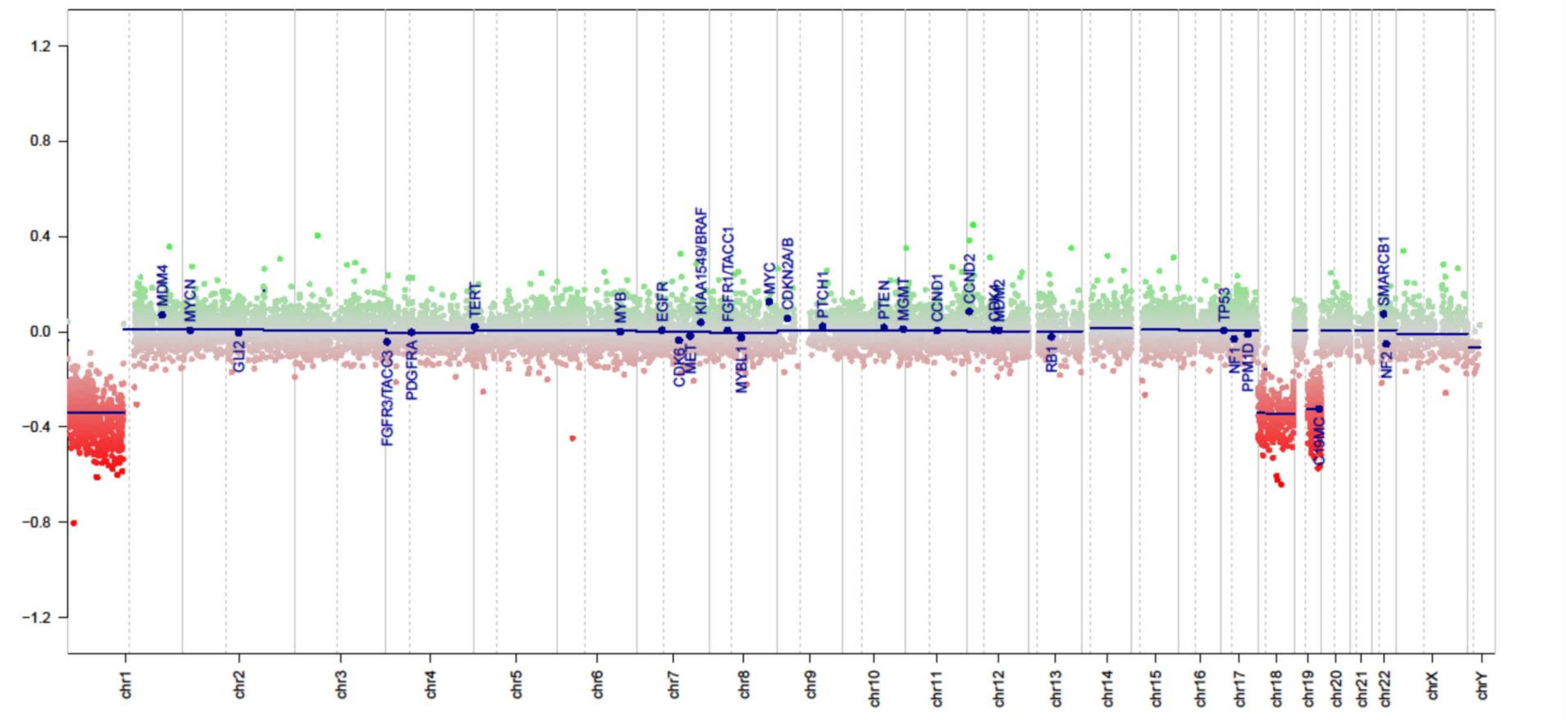
1.2
0.8
0.4
0.0
-0.4
-0.8
-1.2
MDM4
MYCN
GLI2
FGFR3/TACC3
PDGFRA
TERT
MYB
EGFR
CDK6
MET
KIAA1549/BRAF
FGFR1/TACC1
MYBL1
MYC
CDKN2A/B
PTCH1
PTEN
MGMT
CCND1
CCND2
CDK4
MDM2
RB1
TP53
NF1
PPM1D
C19MC
SMARCB1
NF2
chr1
chr2
chr3
chr4
chr5
chr6
chr7
chr8
chr9
chr10
chr11
chr12
chr13
chr14
chr15
chr16
chr17
chr18
chr19
chr20
chr21
chr22
chrX
chrY

HG-10

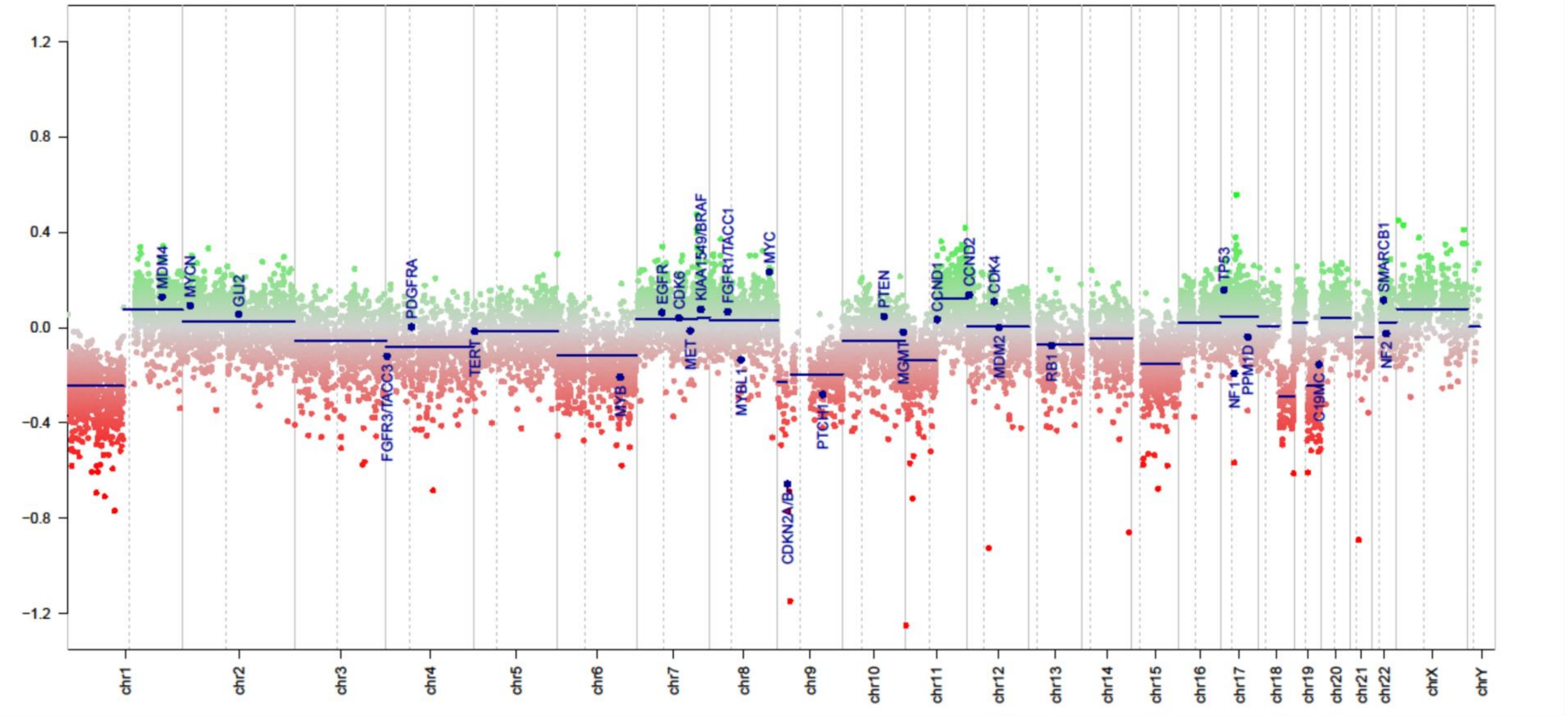

1.2
0.8
0.4
0.0
−0.4
−0.8
−1.2
MDM4
MYCN
GLI2
PDGFRA
FGFR3/TACC3
TERT
MYB
EGFR
CDK6
MET
KIAA1549/BRAF
FGFR1/TACC1
MYBL1
MYC
CDKN2A/B
PTCH1
PTEN
MGMT
CCND1
CCND2
CDK4
MDM2
RB1
TP53
NF1
PPM1D
C19MC
SMARCB1
NF2
chr1
chr2
chr3
chr4
chr5
chr6
chr7
chr8
chr9
chr10
chr11
chr12
chr13
chr14
chr15
chr16
chr17
chr18
chr19
chr20
chr21
chr22
chrX
chrY

HG-11

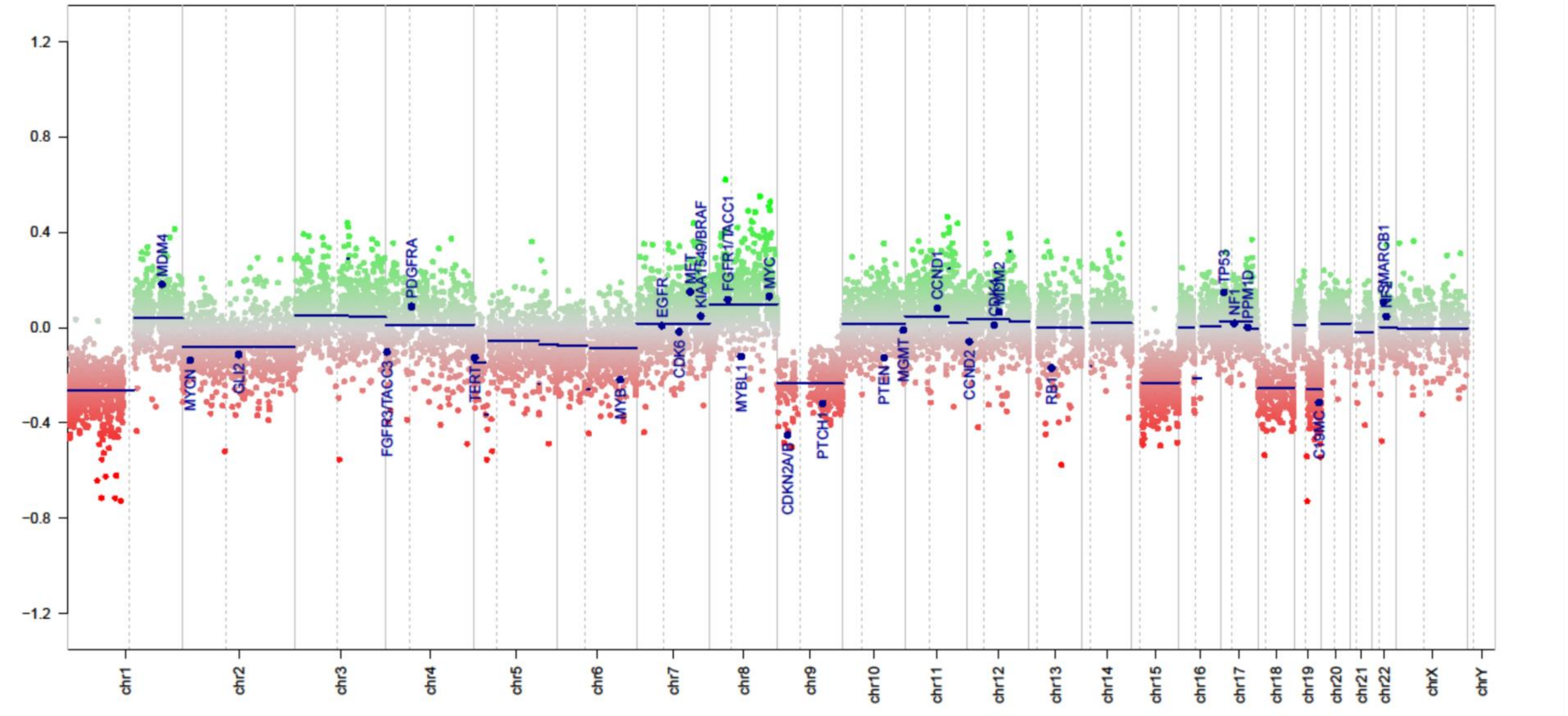
1.2
0.8
0.4
0.0
−0.4
−0.8
−1.2
MDM4
MYCN
GLI2
FGFR3/TACC3
PDGFRA
TERT
MYB
EGFR
CDK6
MET
KIAA1549/BRAF
FGFR1/TACC1
MYBL1
MYC
CDKN2A/B
PTCH1
PTEN
MGMT
CCND1
CCND2
CDK4
MDM2
RB1
TP53
NF1
PPM1D
C19MC
SMARCB1
chr1
chr2
chr3
chr4
chr5
chr6
chr7
chr8
chr9
chr10
chr11
chr12
chr13
chr14
chr15
chr16
chr17
chr18
chr19
chr20
chr21
chr22
chrX
chrY

HG-12

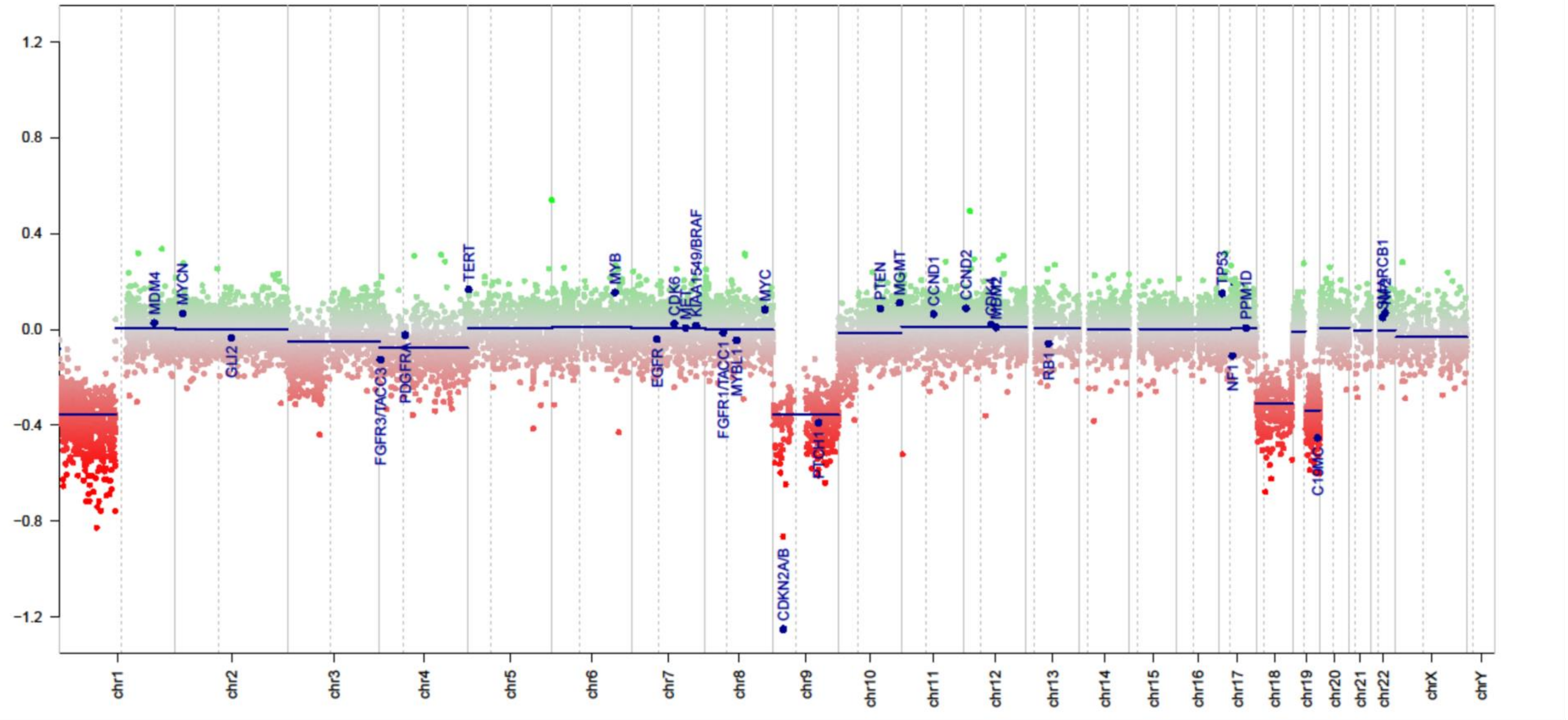

1.2
0.8
0.4
0.0
−0.4
−0.8
−1.2
MDM4
MYCN
GLI2
FGFR3/TACC3
PDGFRA
TERT
MYB
EGFR
CDK6
MET
KIAA1549/BRAF
FGFR1/TACC1
MYBL1
MYC
CDKN2A/B
PTCH1
PTEN
MGMT
CCND1
CCND2
CDK4
MDM2
RB1
TP53
NF1
PPM1D
C19MC
SMARCB1
chr1
chr2
chr3
chr4
chr5
chr6
chr7
chr8
chr9
chr10
chr11
chr12
chr13
chr14
chr15
chr16
chr17
chr18
chr19
chr20
chr21
chr22
chrX
chrY

HG-13

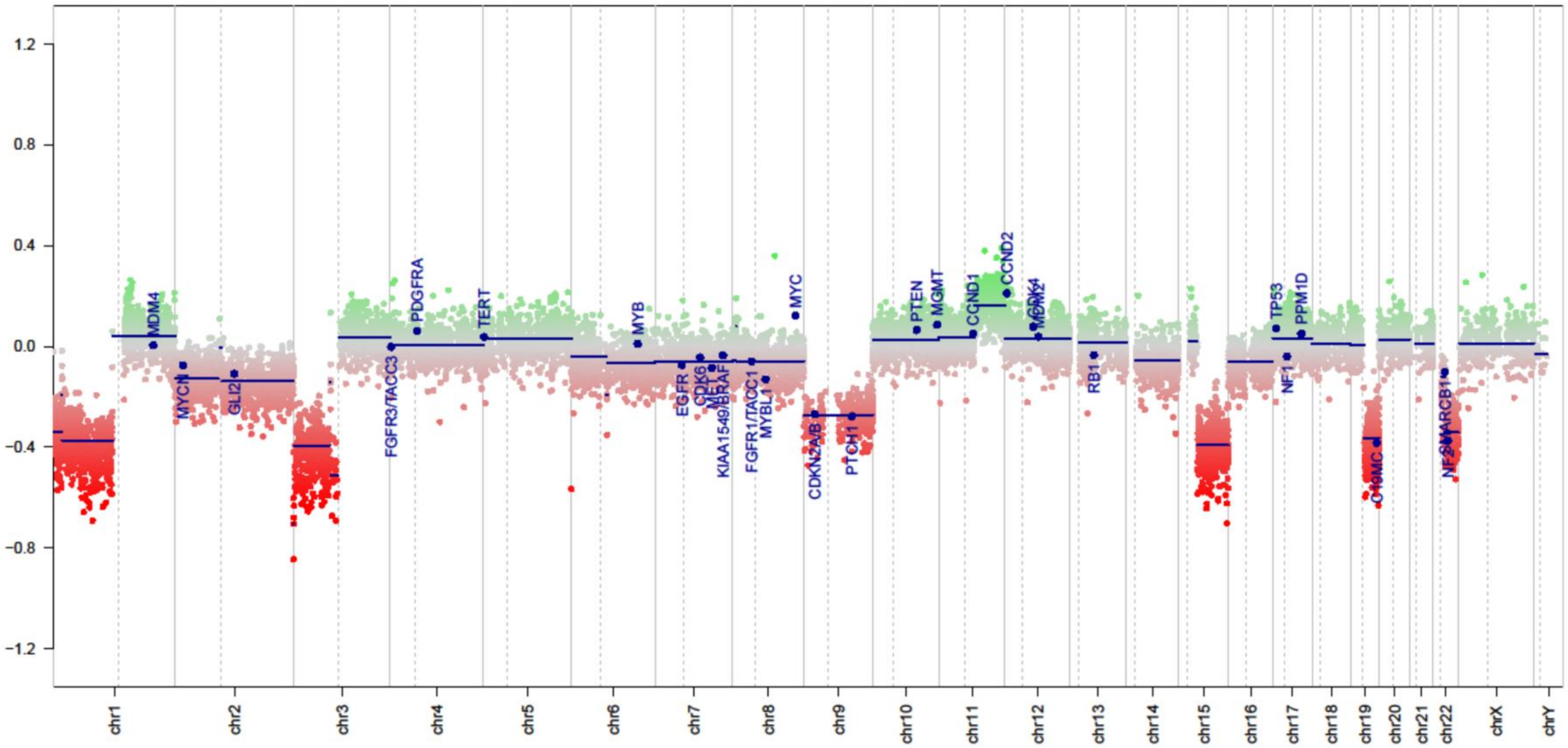

1.2
0.8
0.4
0.0
-0.4
-0.8
-1.2
MDM4
MYCN
GLI2
FGFR3/TACC3
PDGFRA
TERT
MYB
EGFR
CDK6
MET
KIAA1549/BRAF
FGFR1/TACC1
MYBL1
MYC
CDKN2A/B
PTCH1
PTEN
MGMT
CCND1
CCND2
CDK4
MDM2
RB1
TP53
NF1
PPM1D
C19MC
SMARCB1
NF2
chr1
chr2
chr3
chr4
chr5
chr6
chr7
chr8
chr9
chr10
chr11
chr12
chr13
chr14
chr15
chr16
chr17
chr18
chr19
chr20
chr21
chr22
chrX
chrY

HG-14

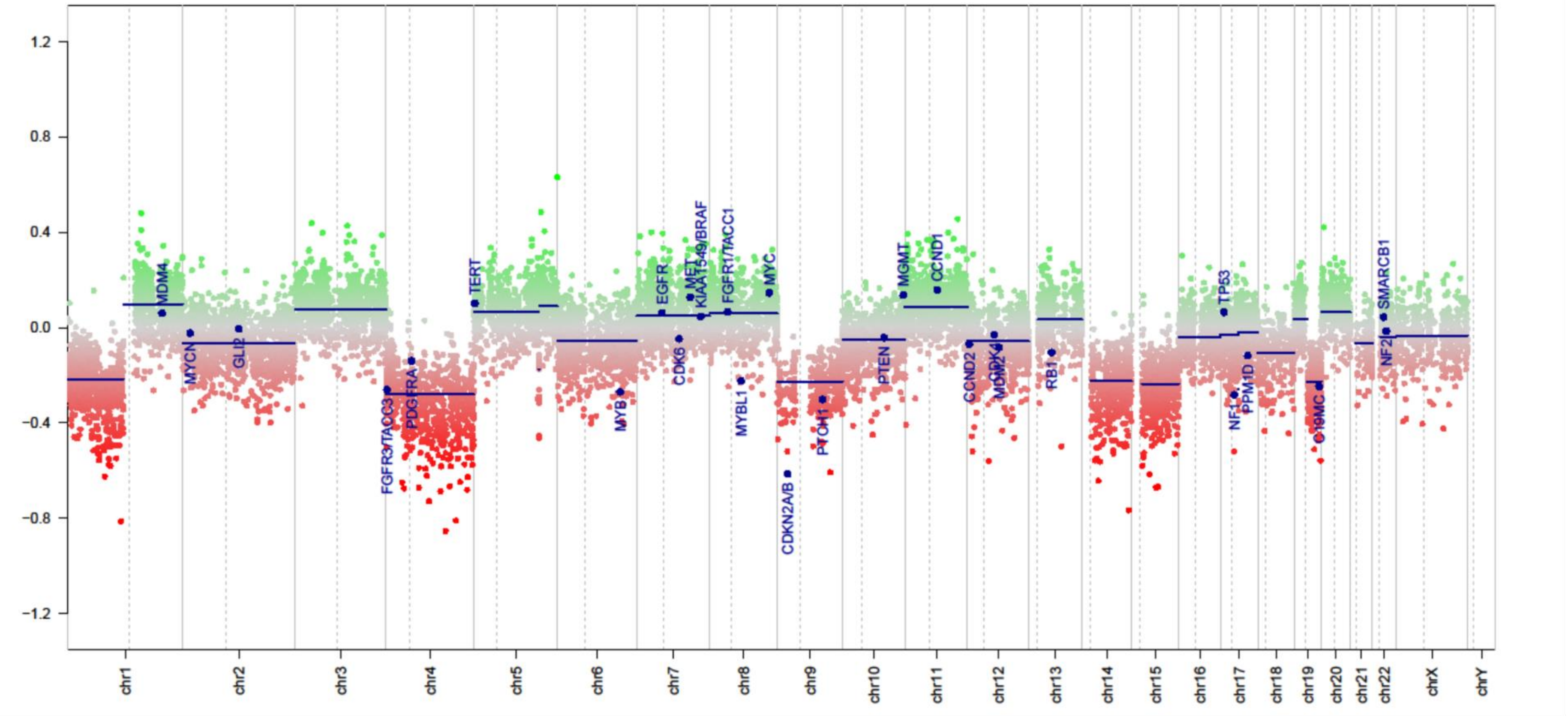
1.2
0.8
0.4
0.0
-0.4
-0.8
-1.2
MDM4
MYCN
GLI2
FGFR3/TACC3
PDGFRA
TERT
MYB
EGFR
CDK6
MET
KIAA1549/BRAF
FGFR1/TACC1
MYBL1
MYC
CDKN2A/B
PTCH1
PTEN
MGMT
CCND1
CCND2
CDK4
MDM2
RB1
TP53
NF1
PPM1D
C19MC
SMARCB1
NF2
chr1
chr2
chr3
chr4
chr5
chr6
chr7
chr8
chr9
chr10
chr11
chr12
chr13
chr14
chr15
chr16
chr17
chr18
chr19
chr20
chr21
chr22
chrX
chrY

HG-15

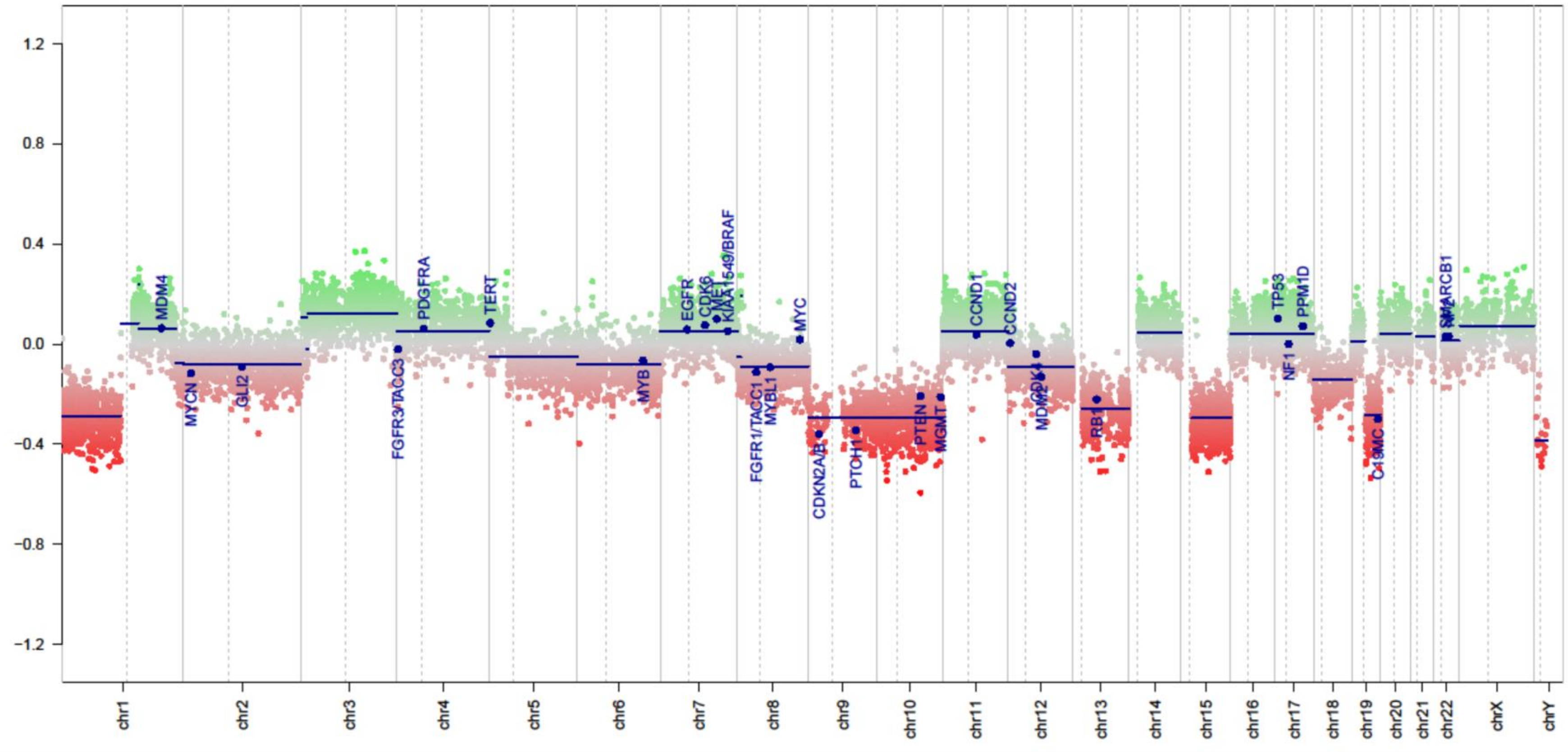

1.2
0.8
0.4
0.0
−0.4
−0.8
−1.2
MDM4
MYCN
GLI2
FGFR3/TACC3
PDGFRA
TERT
MYB
EGFR
CDK6
MET
KIAA1549/BRAF
FGFR1/TACC1
MYBL1
MYC
CDKN2A/B
PTCH1
PTEN
MGMT
CCND1
CCND2
CDK4
MDM2
RB1
TP53
NF1
PPM1D
C19MC
SMARCB1
chr1
chr2
chr3
chr4
chr5
chr6
chr7
chr8
chr9
chr10
chr11
chr12
chr13
chr14
chr15
chr16
chr17
chr18
chr19
chr20
chr21
chr22
chrX
chrY

HG-16

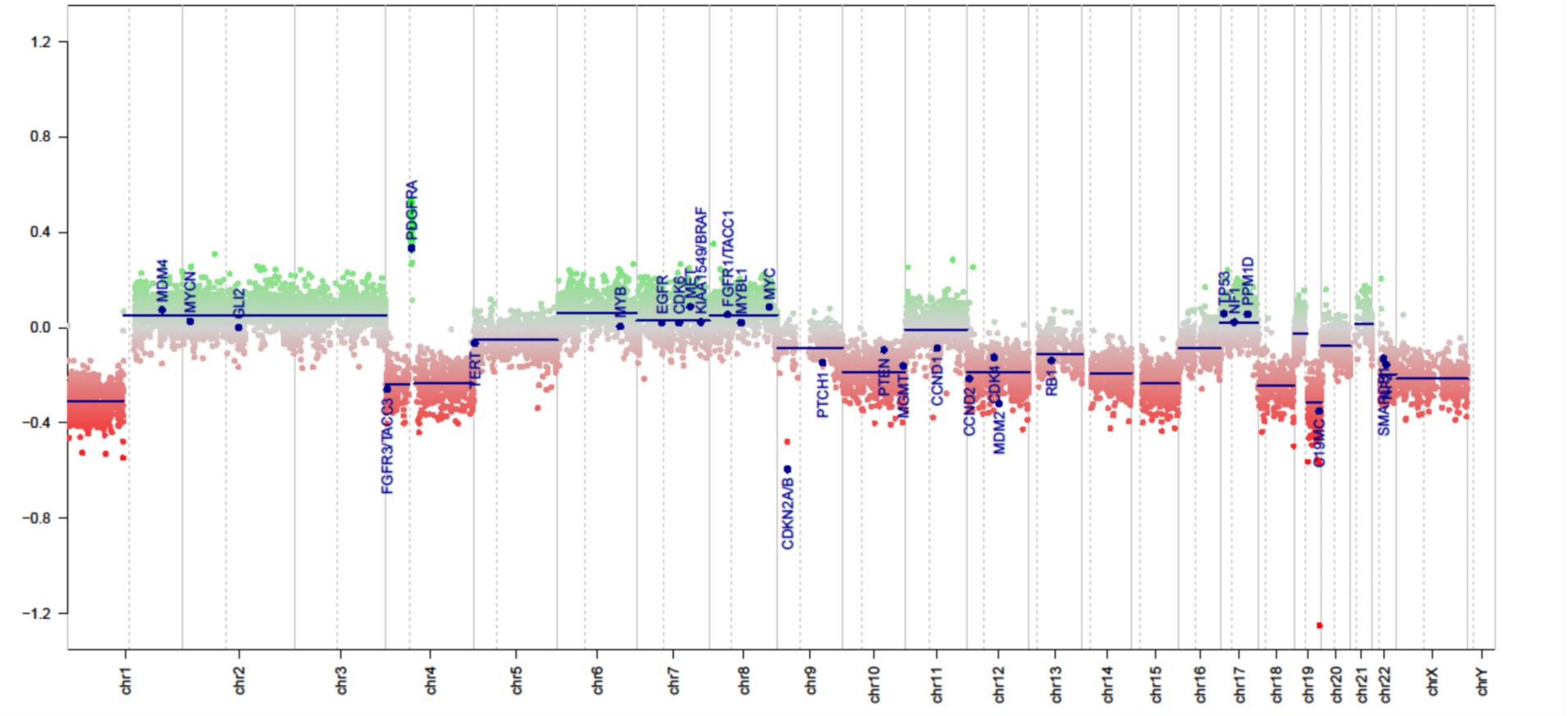

1.2
0.8
0.4
0.0
−0.4
−0.8
−1.2
MDM4
MYCN
GLI2
PDGFRA
FGFR3/TACC3
TERT
MYB
EGFR
CDK6
MET
KIAA1549/BRAF
FGFR1/TACC1
MYBL1
MYC
CDKN2A/B
PTCH1
PTEN
MGMT
CCND1
CCND2
CDK4
MDM2
RB1
TP53
NF1
PPM1D
C19MC
SMARCB1
chr1
chr2
chr3
chr4
chr5
chr6
chr7
chr8
chr9
chr10
chr11
chr12
chr13
chr14
chr15
chr16
chr17
chr18
chr19
chr20
chr21
chr22
chrX
chrY

HG-17

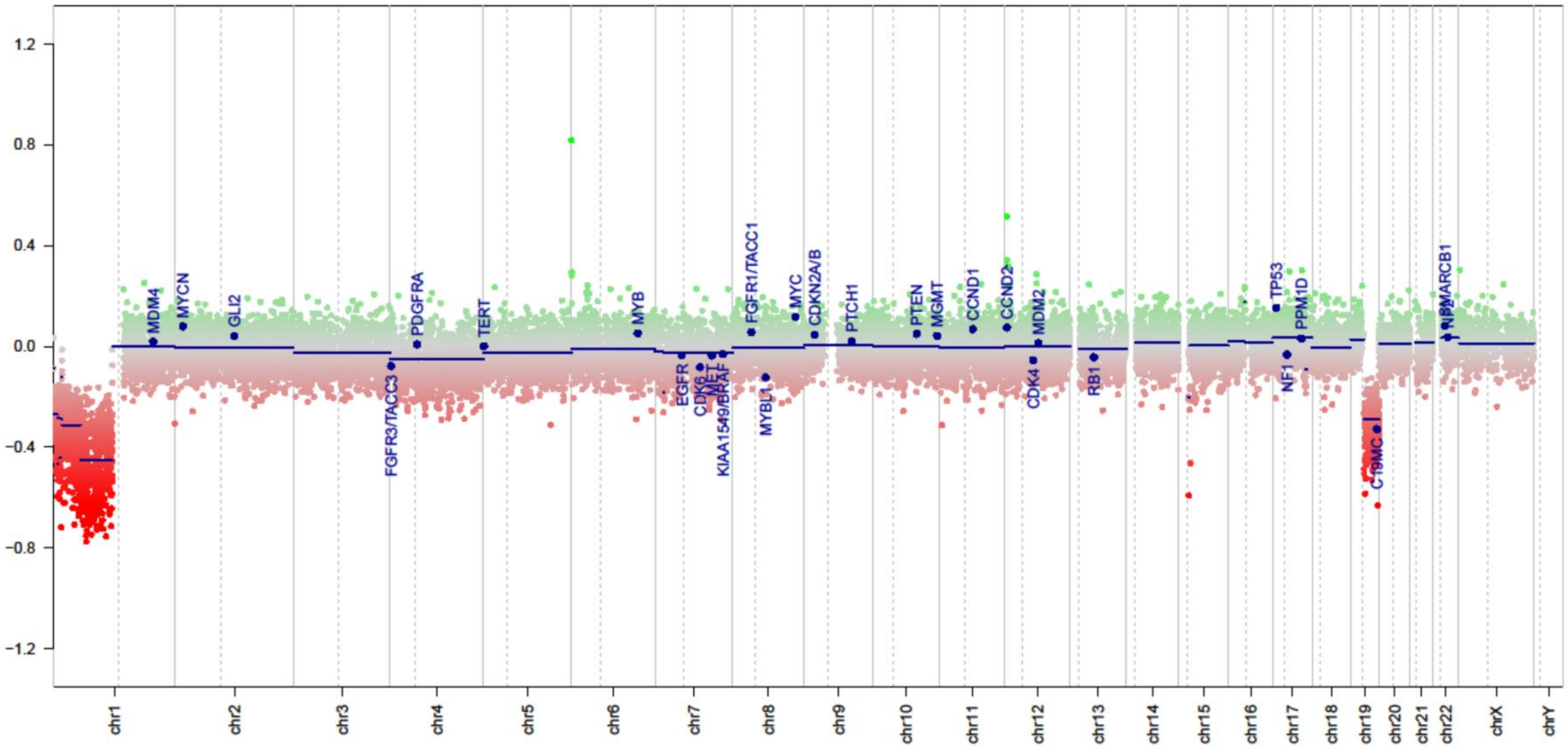

1.2
0.8
0.4
0.0
−0.4
−0.8
−1.2
MDM4
MYCN
GLI2
PDGFRA
FGFR3/TACC3
TERT
MYB
EGFR
CDK6
MET
KIAA1549/BRAF
FGFR1/TACC1
MYBL1
MYC
CDKN2A/B
PTCH1
PTEN
MGMT
CCND1
CCND2
MDM2
CDK4
RB1
TP53
PPM1D
NF1
C19MC
NF2
SMARCB1
chr1
chr2
chr3
chr4
chr5
chr6
chr7
chr8
chr9
chr10
chr11
chr12
chr13
chr14
chr15
chr16
chr17
chr18
chr19
chr20
chr21
chr22
chrX
chrY

HG-18

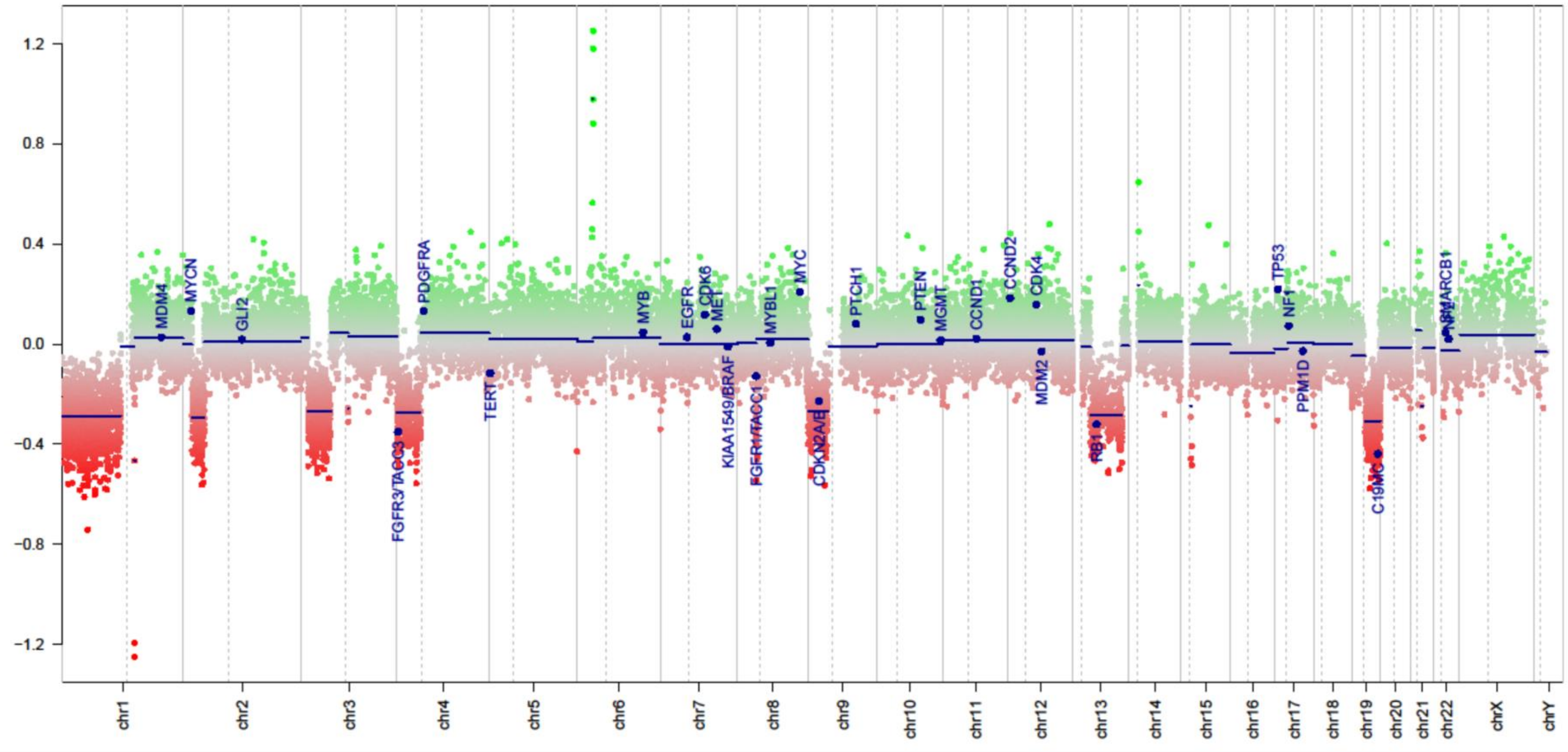

1.2
0.8
0.4
0.0
−0.4
−0.8
−1.2
MDM4
MYCN
GLI2
PDGFRA
FGFR3/TACC3
TERT
MYB
EGFR
CDK6
MET
KIAA1549/BRAF
FGFR1/TACC1
MYBL1
MYC
CDKN2A/B
PTCH1
PTEN
MGMT
CCND1
CCND2
CDK4
MDM2
RB1
TP53
NF1
PPM1D
C19MC
SMARCB1
chr1
chr2
chr3
chr4
chr5
chr6
chr7
chr8
chr9
chr10
chr11
chr12
chr13
chr14
chr15
chr16
chr17
chr18
chr19
chr20
chr21
chr22
chrX
chrY

HG-19

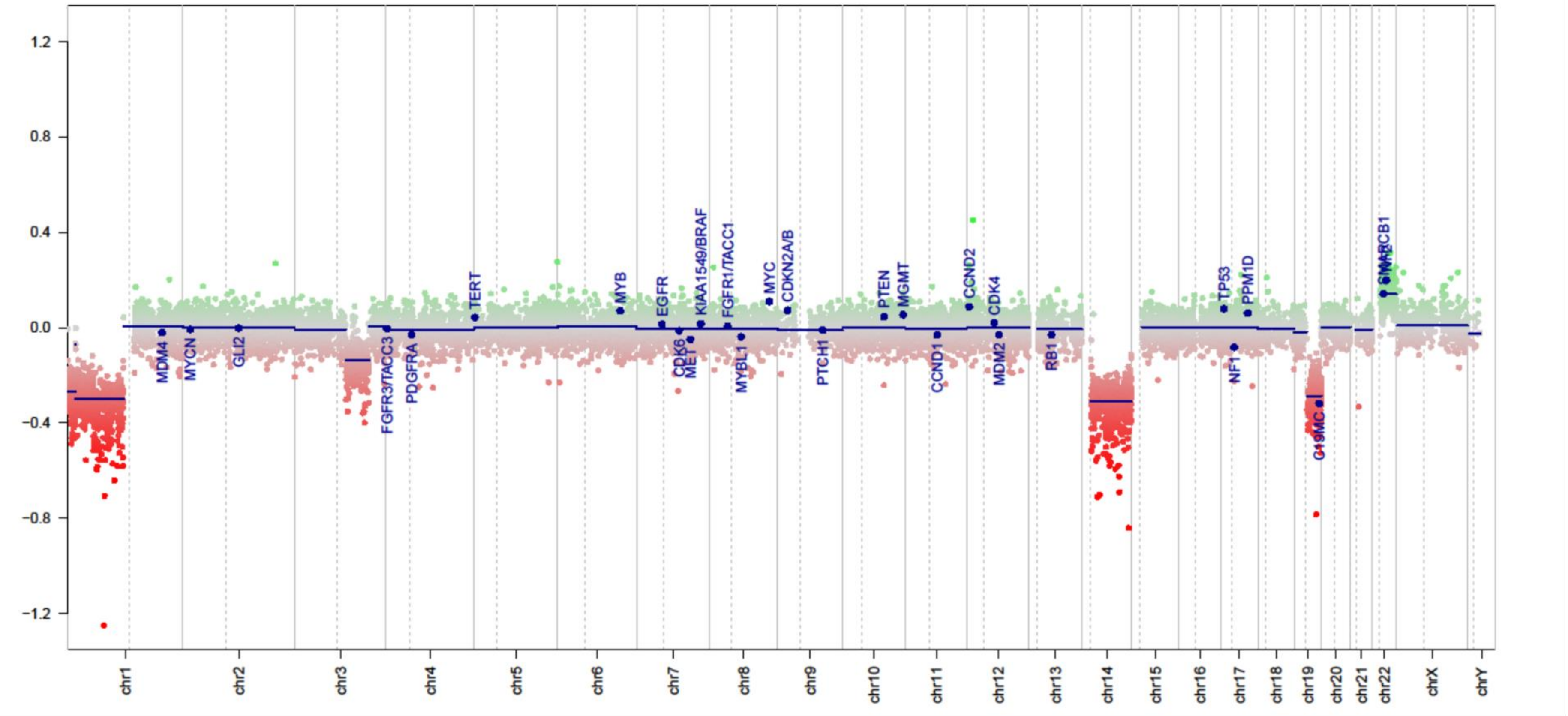
1.2
0.8
0.4
0.0
−0.4
−0.8
−1.2
MDM4
MYCN
GLI2
FGFR3/TACC3
PDGFRA
TERT
MYB
EGFR
CDK6
MET
KIAA1549/BRAF
FGFR1/TACC1
MYBL1
MYC
CDKN2A/B
PTCH1
PTEN
MGMT
CCND1
CCND2
CDK4
MDM2
RB1
TP53
NF1
PPM1D
C19MC
SMARCB1
chr1
chr2
chr3
chr4
chr5
chr6
chr7
chr8
chr9
chr10
chr11
chr12
chr13
chr14
chr15
chr16
chr17
chr18
chr19
chr20
chr21
chr22
chrX
chrY

HG-20

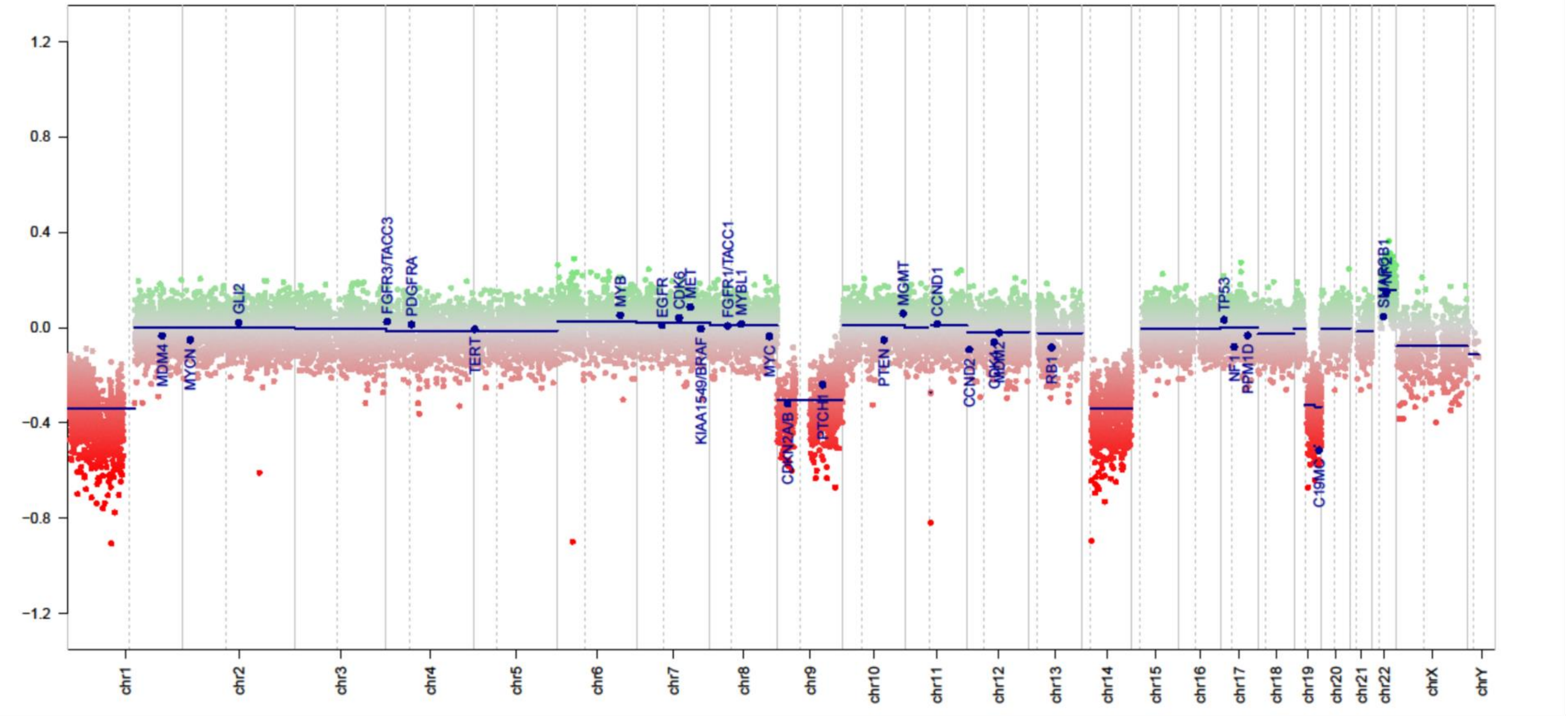
1.2
0.8
0.4
0.0
-0.4
-0.8
-1.2
MDM4
MYCN
GLI2
FGFR3/TACC3
PDGFRA
TERT
MYB
EGFR
CDK6
MET
KIAA1549/BRAF
FGFR1/TACC1
MYBL1
MYC
CDKN2A/B
PTCH1
PTEN
MGMT
CCND1
CCND2
CDK4
MDM2
RB1
TP53
NF1
PPM1D
C19MC
SMARCB1
NF2
chr1
chr2
chr3
chr4
chr5
chr6
chr7
chr8
chr9
chr10
chr11
chr12
chr13
chr14
chr15
chr16
chr17
chr18
chr19
chr20
chr21
chr22
chrX
chrY

HG-21

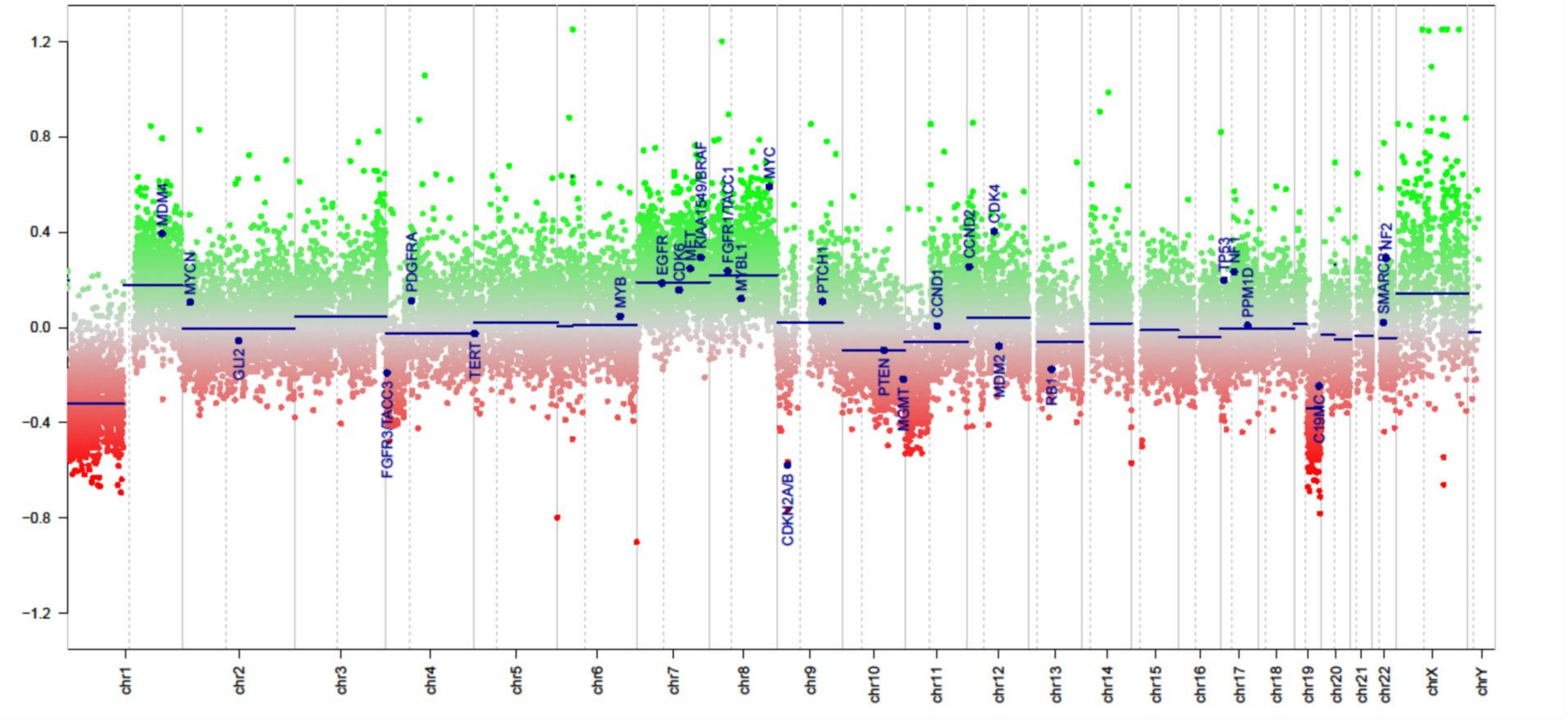

1.2
0.8
0.4
0.0
-0.4
-0.8
-1.2
MDM4
MYCN
GLI2
FGFR3/TACC3
PDGFRA
TERT
MYB
EGFR
CDK6
MET
KIAA1549/BRAF
FGFR1/TACC1
MYBL1
MYC
CDKN2A/B
PTCH1
PTEN
MGMT
CCND1
CCND2
CDK4
MDM2
RB1
TP53
NF1
PPM1D
C19MC
SMARCB1
NF2
chr1
chr2
chr3
chr4
chr5
chr6
chr7
chr8
chr9
chr10
chr11
chr12
chr13
chr14
chr15
chr16
chr17
chr18
chr19
chr20
chr21
chr22
chrX
chrY

HG-22

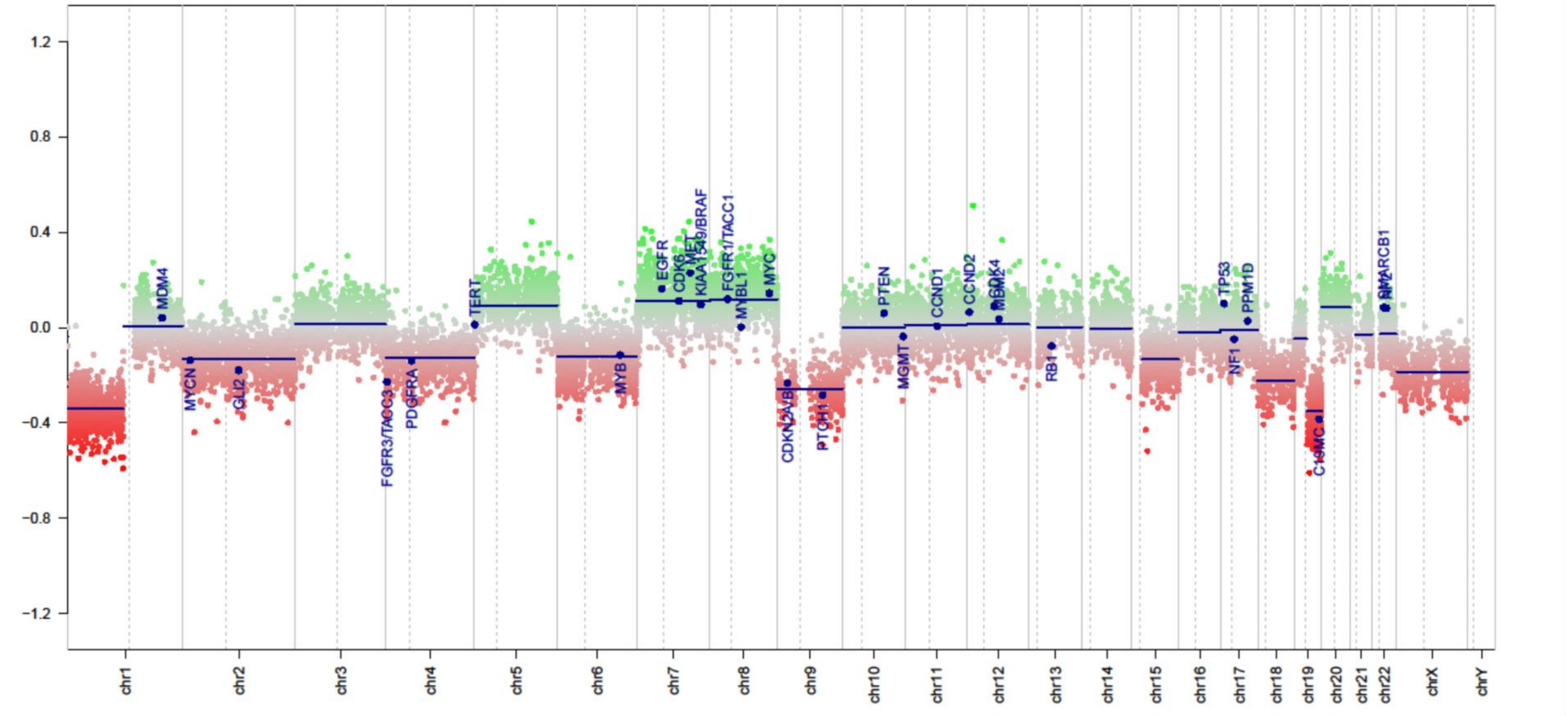

1.2
0.8
0.4
0.0
−0.4
−0.8
−1.2
MDM4
MYCN
GLI2
FGFR3/TACC3
PDGFRA
TERT
MYB
EGFR
CDK6
MET
KIAA1549/BRAF
FGFR1/TACC1
MYBL1
MYC
PTCH1
PTEN
MGMT
CCND1
CCND2
CDK4
MDM2
RB1
TP53
PPM1D
NF1
SMARCB1
NF2
chr1
chr2
chr3
chr4
chr5
chr6
chr7
chr8
chr9
chr10
chr11
chr12
chr13
chr14
chr15
chr16
chr17
chr18
chr19
chr20
chr21
chr22
chrX
chrY

HG-23

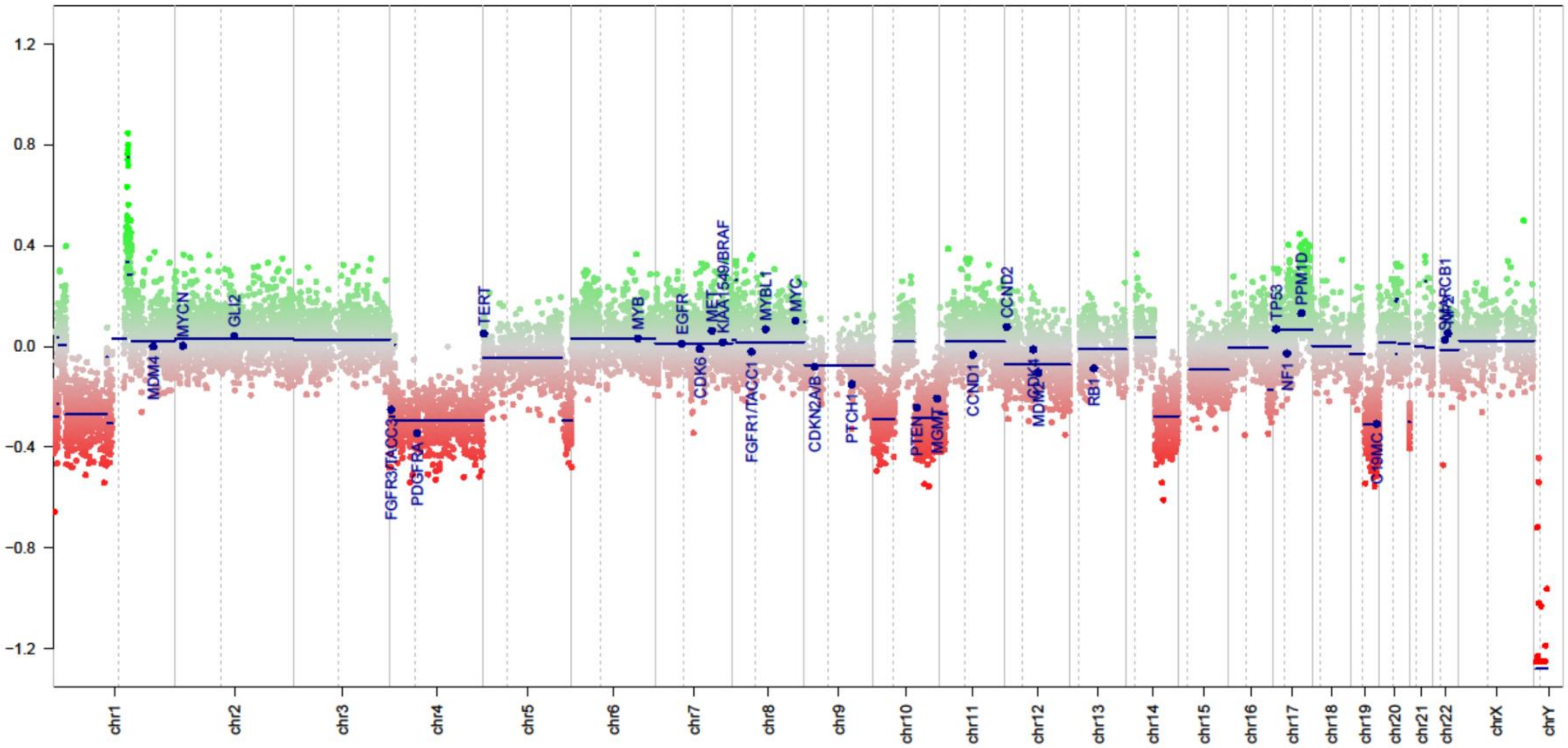

1.2
0.8
0.4
0.0
−0.4
−0.8
−1.2
MDM4
MYCN
GLI2
FGFR3/TACC3
PDGFRA
TERT
MYB
EGFR
CDK6
MET
KIAA1549/BRAF
FGFR1/TACC1
MYBL1
MYC
CDKN2A/B
PTCH1
PTEN
MGMT
CCND1
CCND2
CDK4
MDM2
RB1
TP53
NF1
PPM1D
chr1
chr2
chr3
chr4
chr5
chr6
chr7
chr8
chr9
chr10
chr11
chr12
chr13
chr14
chr15
chr16
chr17
chr18
chr19
chr20
chr21
chr22
chrX
chrY

HG-24

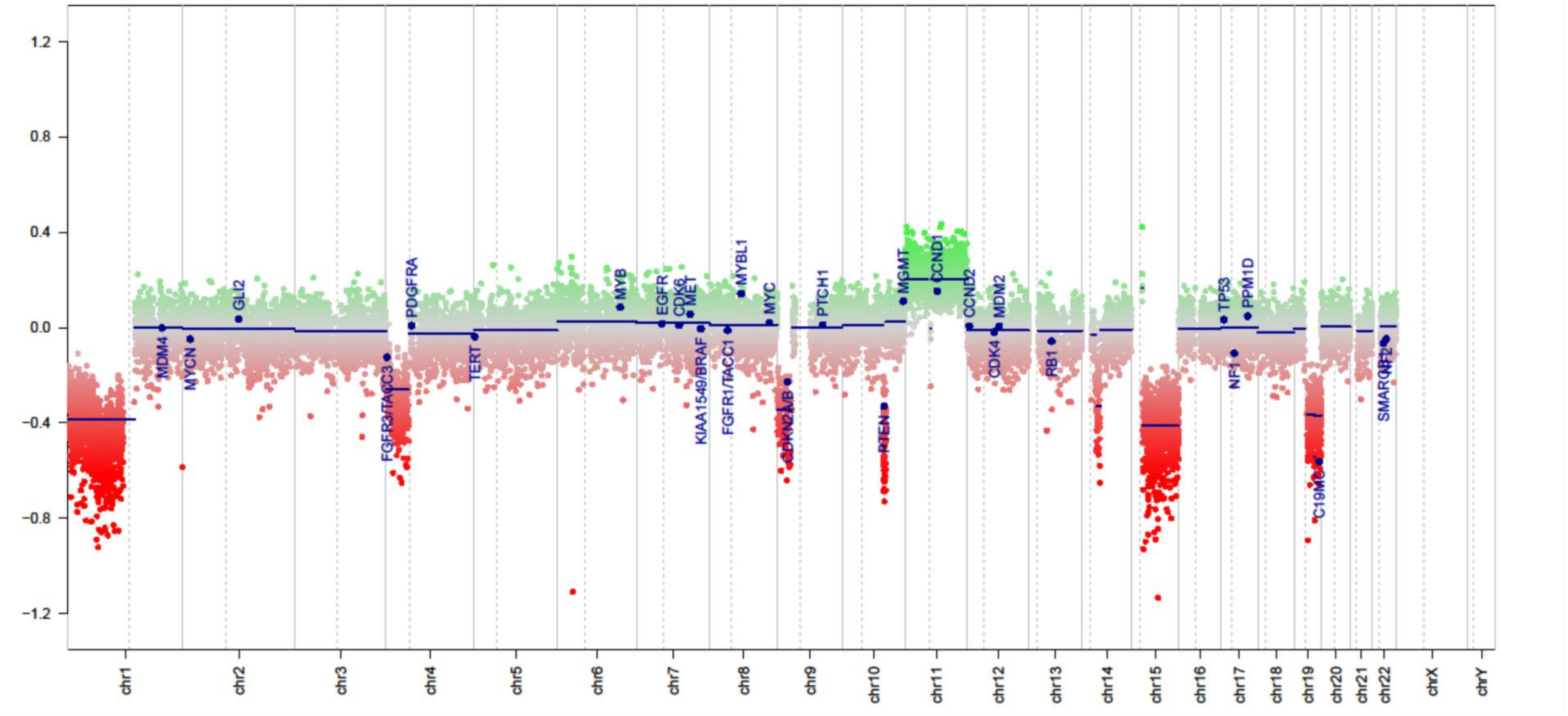

1.2
0.8
0.4
0.0
-0.4
-0.8
-1.2
MDM4
MYCN
GLI2
FGFR3/TACC3
PDGFRA
TERT
MYB
EGFR
CDK6
MET
KIAA1549/BRAF
FGFR1/TACC1
MYBL1
MYC
CDKN2A/B
PTCH1
PTEN
MGMT
CCND1
CCND2
CDK4
MDM2
RB1
TP53
NF1
PPM1D
C19MC
SMARCB1
NF2
chr1
chr2
chr3
chr4
chr5
chr6
chr7
chr8
chr9
chr10
chr11
chr12
chr13
chr14
chr15
chr16
chr17
chr18
chr19
chr20
chr21
chr22
chrX
chrY

HG-25

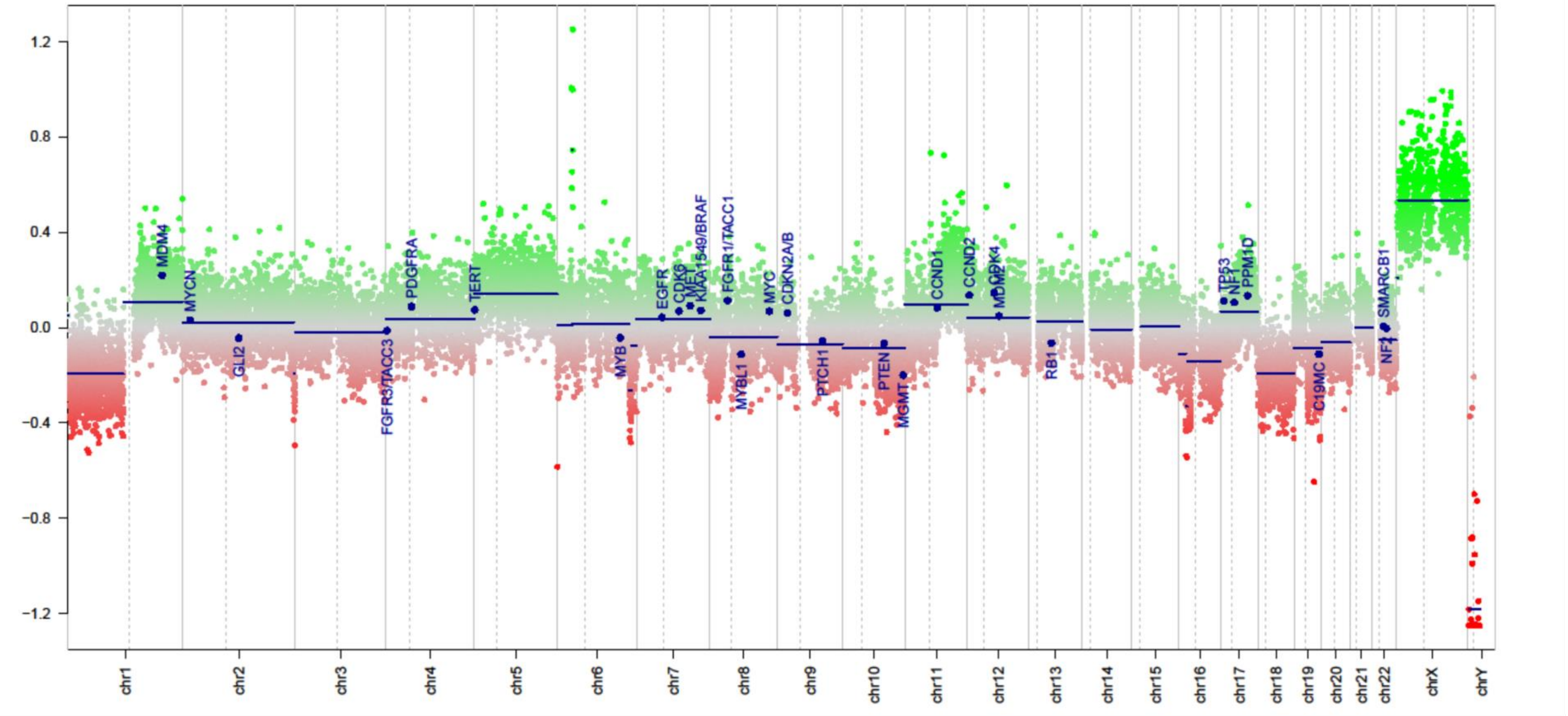

1.2
0.8
0.4
0.0
-0.4
-0.8
-1.2
MDM4
MYCN
GLI2
FGFR3/TACC3
PDGFRA
TERT
MYB
EGFR
CDK6
MET
KIAA1549/BRAF
FGFR1/TACC1
MYBL1
MYC
CDKN2A/B
PTCH1
PTEN
MGMT
CCND1
CCND2
CDK4
MDM2
RB1
TP53
NF1
PPM1D
C19MC
SMARCB1
NF2
chr1
chr2
chr3
chr4
chr5
chr6
chr7
chr8
chr9
chr10
chr11
chr12
chr13
chr14
chr15
chr16
chr17
chr18
chr19
chr20
chr21
chr22
chrX
chrY

HG-26

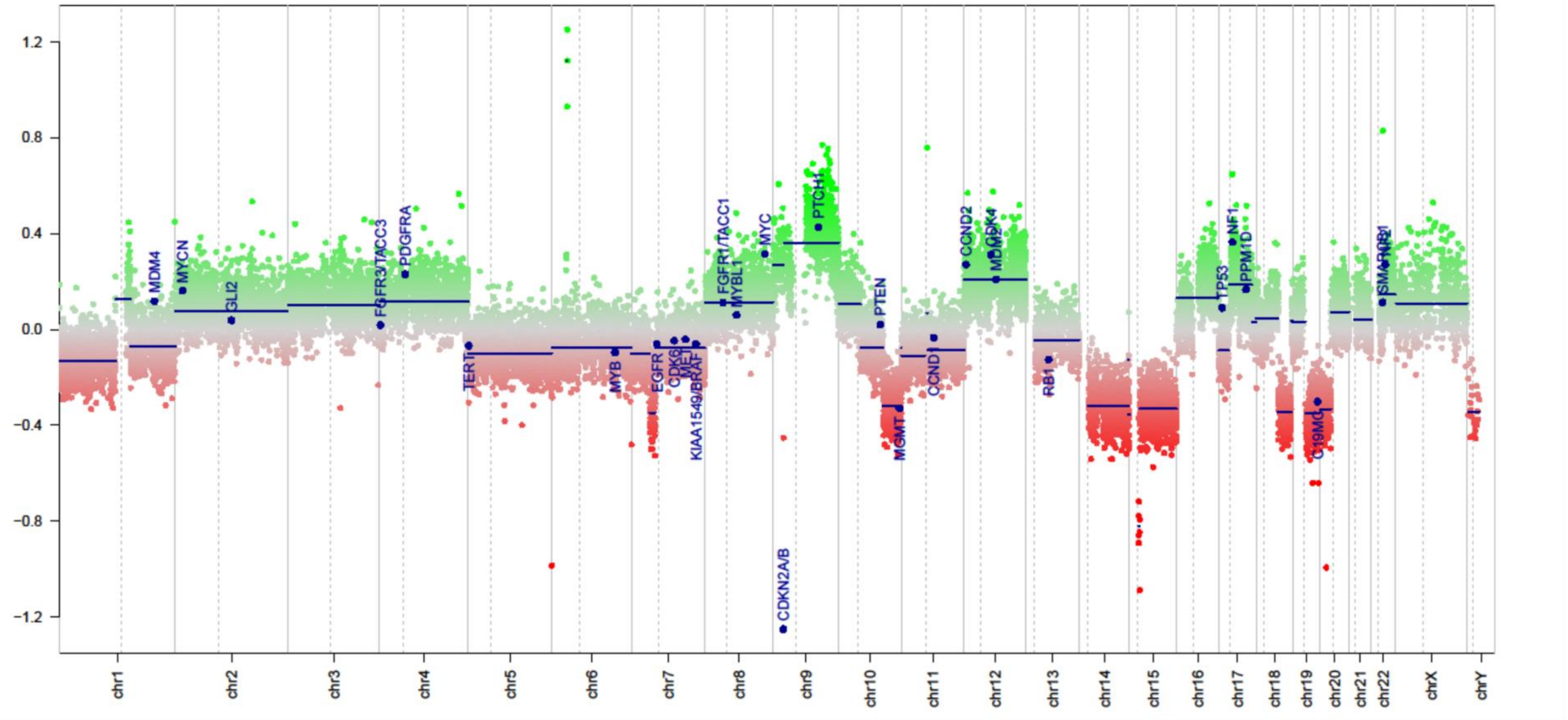

1.2
0.8
0.4
0.0
-0.4
-0.8
-1.2
MDM4
MYCN
GLI2
FGFR3/TACC3
PDGFRA
TERT
MYB
EGFR
CDK6
MET
KIAA1549/BRAF
FGFR1/TACC1
MYBL1
MYC
CDKN2A/B
PTCH1
PTEN
MGMT
CCND1
CCND2
CDK4
MDM2
RB1
TP53
NF1
PPM1D
C19MC
chr1
chr2
chr3
chr4
chr5
chr6
chr7
chr8
chr9
chr10
chr11
chr12
chr13
chr14
chr15
chr16
chr17
chr18
chr19
chr20
chr21
chr22
chrX
chrY

HG-27

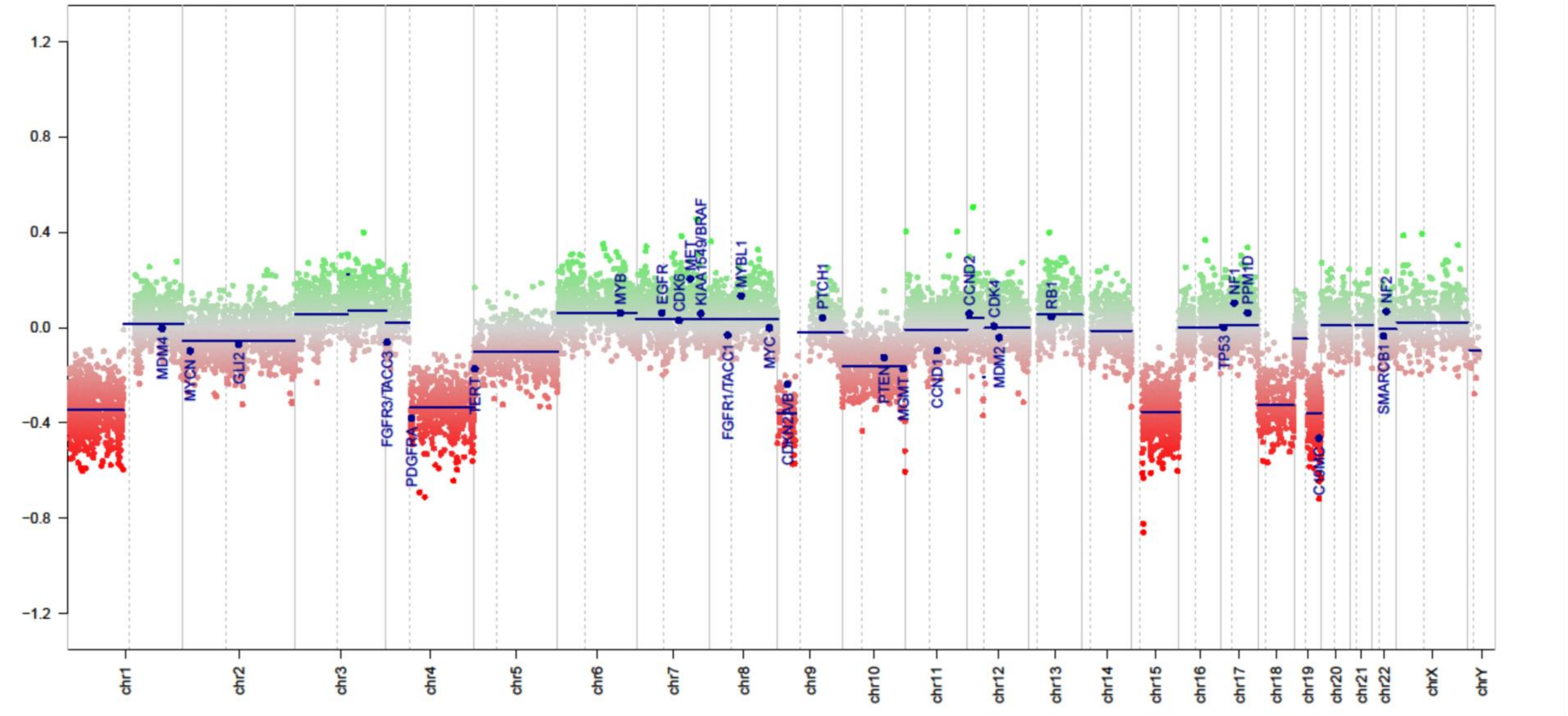
1.2
0.8
0.4
0.0
−0.4
−0.8
−1.2
MDM4
MYCN
GLI2
FGFR3/TACC3
PDGFRA
TERT
MYB
EGFR
CDK6
MET
KIAA1549/BRAF
MYBL1
FGFR1/TACC1
MYC
CDKN2A/B
PTCH1
PTEN
MGMT
CCND1
CCND2
CDK4
MDM2
RB1
TP53
NF1
PPM1D
C19MC
NF2
SMARCB1
chr1
chr2
chr3
chr4
chr5
chr6
chr7
chr8
chr9
chr10
chr11
chr12
chr13
chr14
chr15
chr16
chr17
chr18
chr19
chr20
chr21
chr22
chrX
chrY

HG-28

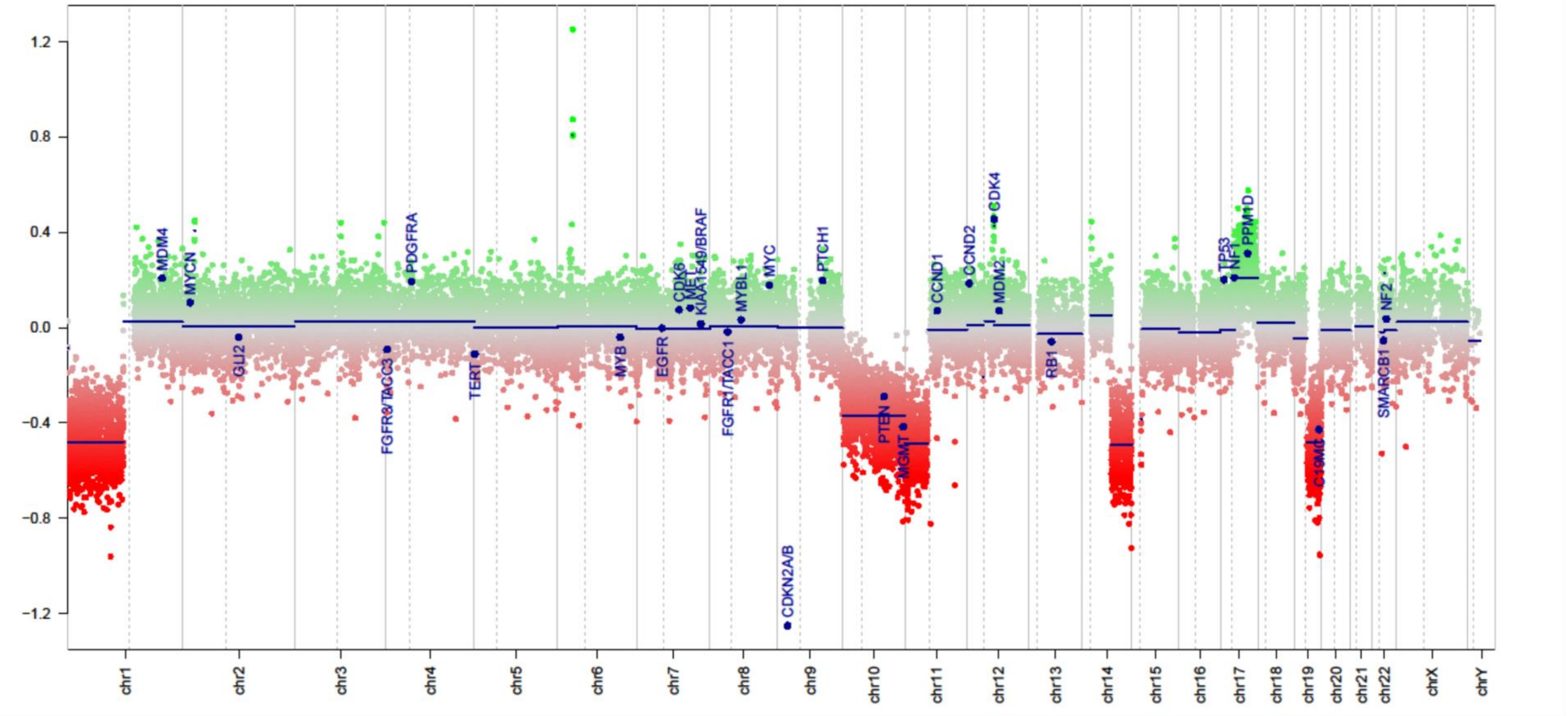

1.2
0.8
0.4
0.0
-0.4
-0.8
-1.2
MDM4
MYCN
GLI2
FGFR3/TACC3
PDGFRA
TERT
MYB
EGFR
CDK6
MET
KIAA1549/BRAF
FGFR1/TACC1
MYBL1
MYC
CDKN2A/B
PTCH1
PTEN
MGMT
CCND1
CCND2
CDK4
MDM2
RB1
TP53
NF1
PPM1D
C19MC
SMARCB1
NF2
chr1
chr2
chr3
chr4
chr5
chr6
chr7
chr8
chr9
chr10
chr11
chr12
chr13
chr14
chr15
chr16
chr17
chr18
chr19
chr20
chr21
chr22
chrX
chrY

HG-29

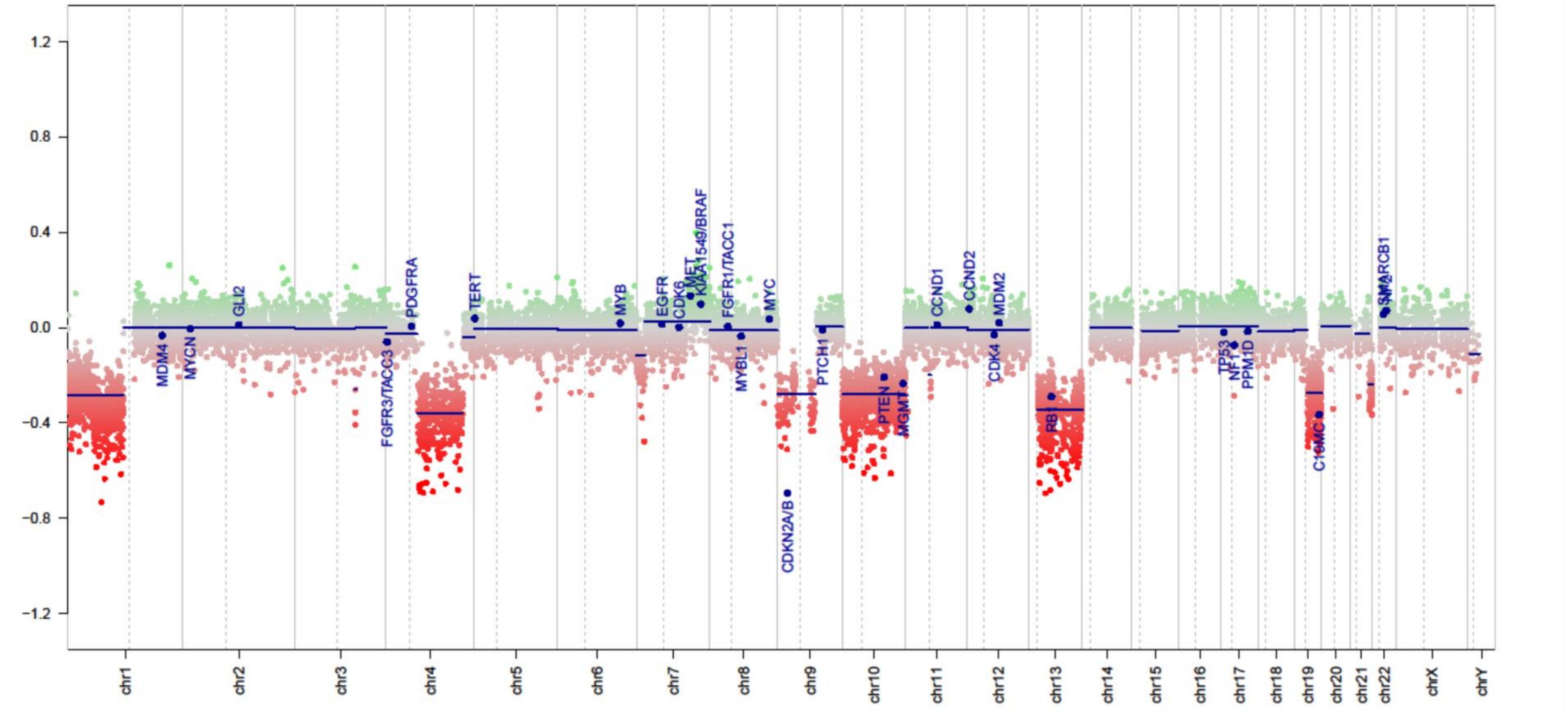
1.2
0.8
0.4
0.0
−0.4
−0.8
−1.2
chr1
chr2
chr3
chr4
chr5
chr6
chr7
chr8
chr9
chr10
chr11
chr12
chr13
chr14
chr15
chr16
chr17
chr18
chr19
chr20
chr21
chr22
chrX
chrY
MDM4
MYCN
GLI2
FGFR3/TACC3
PDGFRA
TERT
MYB
EGFR
CDK6
MET
KIAA1549/BRAF
FGFR1/TACC1
MYBL1
MYC
CDKN2A/B
PTCH1
PTEN
MGMT
CCND1
CCND2
CDK4
MDM2
RB1
TP53
NF1
PPM1D
C19MC
SMARCB1

HG-30

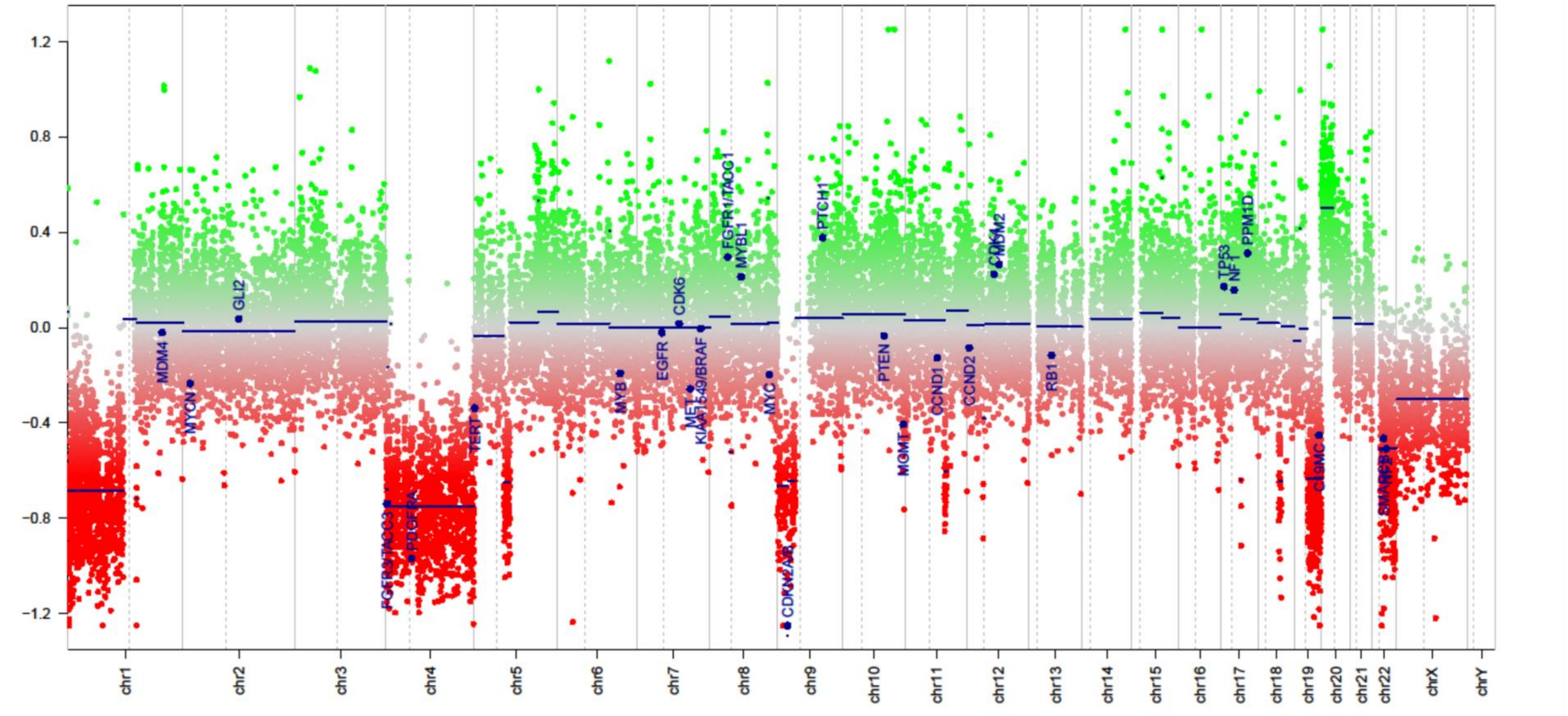

1.2
0.8
0.4
0.0
-0.4
-0.8
-1.2
chr1
chr2
chr3
chr4
chr5
chr6
chr7
chr8
chr9
chr10
chr11
chr12
chr13
chr14
chr15
chr16
chr17
chr18
chr19
chr20
chr21
chr22
chrX
chrY
MDM4
MYCN
GLI2
TERT
PDGFRA
CDK6
EGFR
MYB
FGFR1/TACC1
MYBL1
MYC
PTCH1
PTEN
MGMT
CCND1
CCND2
CDK4
MDM2
RB1
TP53
NF1
PPM1D

HG-31

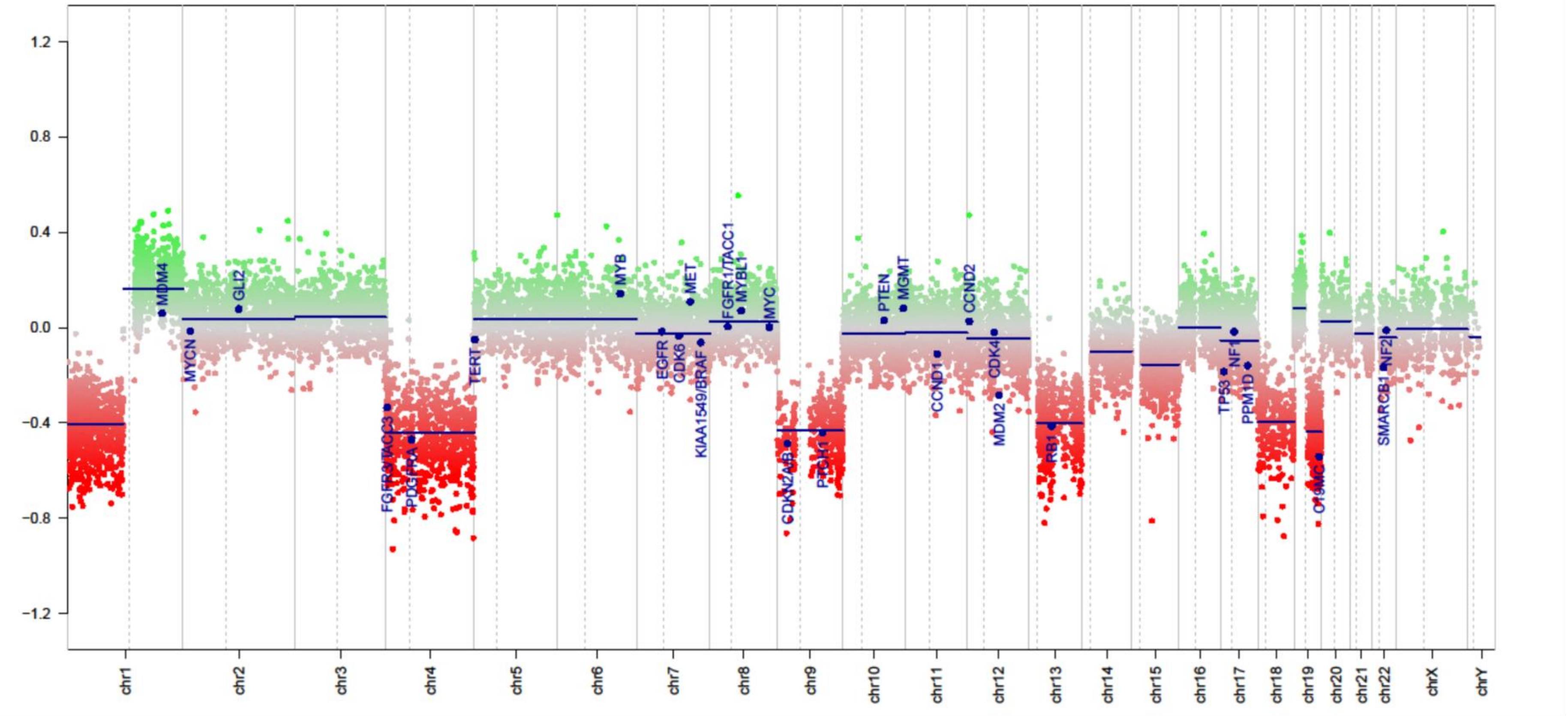

1.2
0.8
0.4
0.0
-0.4
-0.8
-1.2
MDM4
MYCN
GLI2
FGFR3/TACC3
PDGFRA
TERT
MYB
EGFR
CDK6
MET
KIAA1549/BRAF
FGFR1/TACC1
MYBL1
MYC
CDKN2A/B
PTCH1
PTEN
MGMT
CCND1
CCND2
CDK4
MDM2
RB1
TP53
NF1
PPM1D
C19MC
SMARCB1
NF2
chr1
chr2
chr3
chr4
chr5
chr6
chr7
chr8
chr9
chr10
chr11
chr12
chr13
chr14
chr15
chr16
chr17
chr18
chr19
chr20
chr21
chr22
chrX
chrY

HG-32

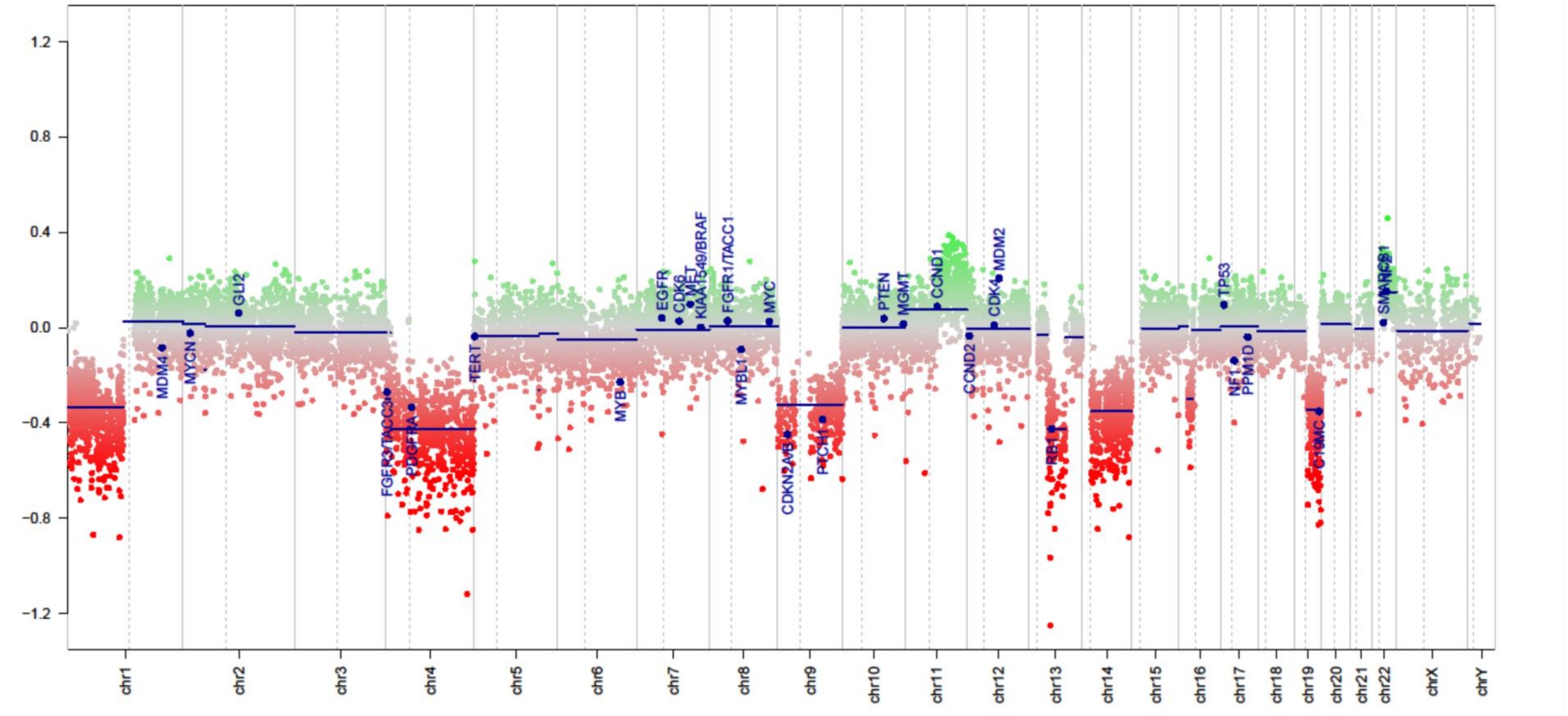

1.2
0.8
0.4
0.0
-0.4
-0.8
-1.2
MDM4
MYCN
GLI2
FGFR3/TACC3
PDGFRA
TERT
MYB
EGFR
CDK6
MET
KIAA1549/BRAF
FGFR1/TACC1
MYBL1
MYC
CDKN2A/B
PTCH1
PTEN
MGMT
CCND1
CCND2
CDK4
MDM2
RB1
TP53
NF1
PPM1D
chr1
chr2
chr3
chr4
chr5
chr6
chr7
chr8
chr9
chr10
chr11
chr12
chr13
chr14
chr15
chr16
chr17
chr18
chr19
chr20
chr21
chr22
chrX
chrY

HG-33

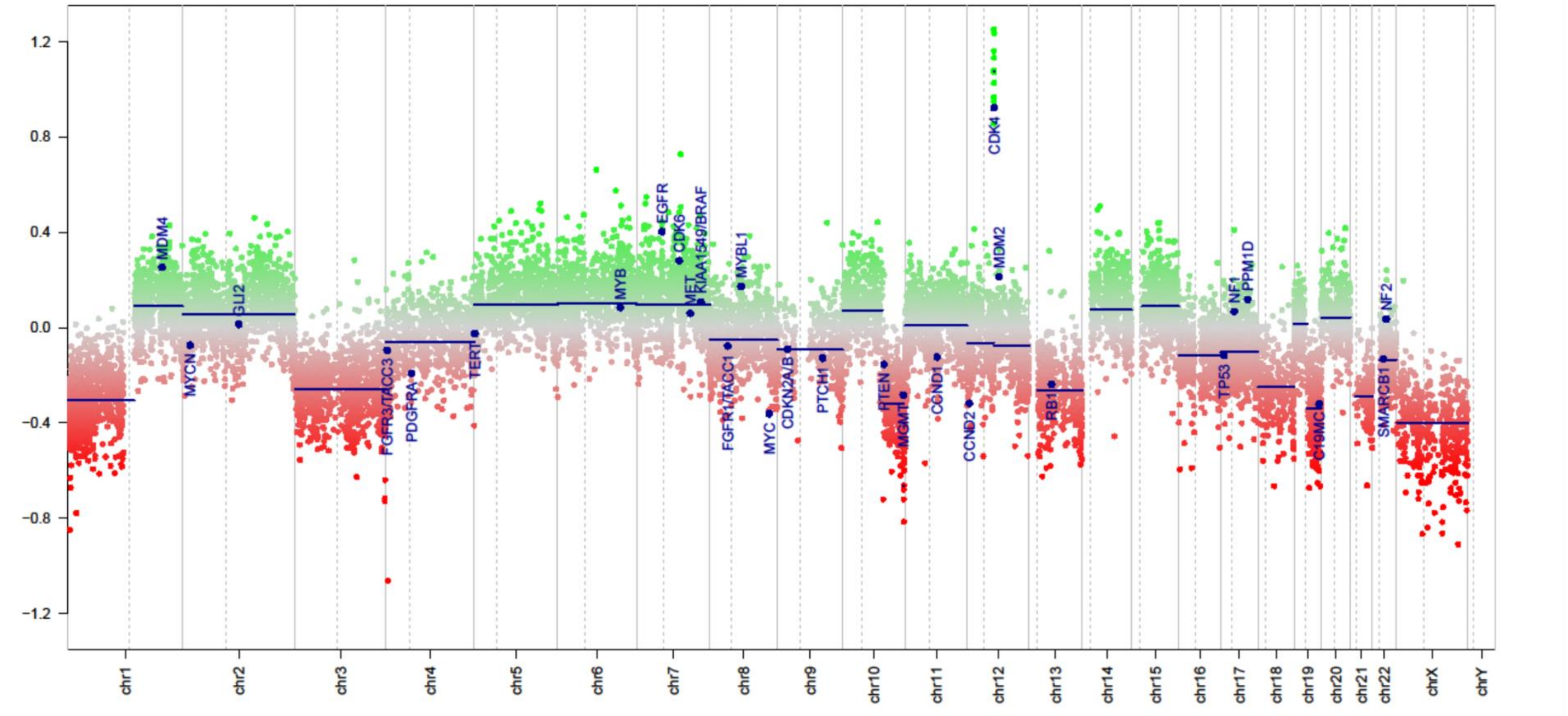
1.2
0.8
0.4
0.0
-0.4
-0.8
-1.2
MDM4
MYCN
GLI2
FGFR3/TACC3
PDGFRA
TERT
MYB
EGFR
CDK6
MET
KIAA1549/BRAF
MYBL1
FGFR1/TACC1
MYC
CDKN2A/B
PTCH1
PTEN
MGMT
CCND1
CCND2
CDK4
MDM2
RB1
TP53
NF1
PPM1D
SMARCB1
NF2
chr1
chr2
chr3
chr4
chr5
chr6
chr7
chr8
chr9
chr10
chr11
chr12
chr13
chr14
chr15
chr16
chr17
chr18
chr19
chr20
chr21
chr22
chrX
chrY

HG-34

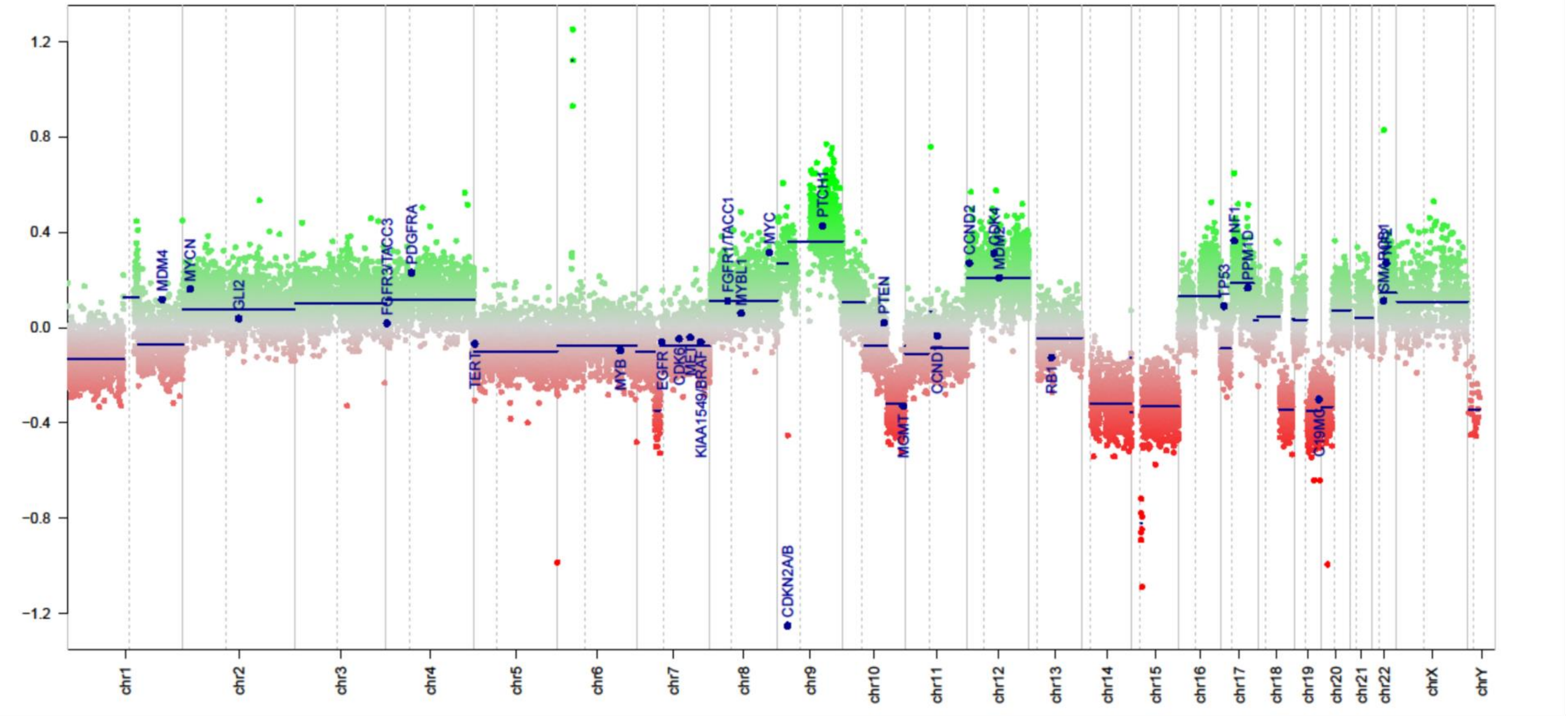

1.2
0.8
0.4
0.0
−0.4
−0.8
−1.2
MDM4
MYCN
GLI2
FGFR3/TACC3
PDGFRA
TERT
MYB
EGFR
CDK6
MET
KIAA1549/BRAF
FGFR1/TACC1
MYBL1
MYC
CDKN2A/B
PTCH1
PTEN
MGMT
CCND1
CCND2
CDK4
MDM2
RB1
TP53
NF1
PPM1D
C19MC
SMARCB1
chr1
chr2
chr3
chr4
chr5
chr6
chr7
chr8
chr9
chr10
chr11
chr12
chr13
chr14
chr15
chr16
chr17
chr18
chr19
chr20
chr21
chr22
chrX
chrY

HG-35

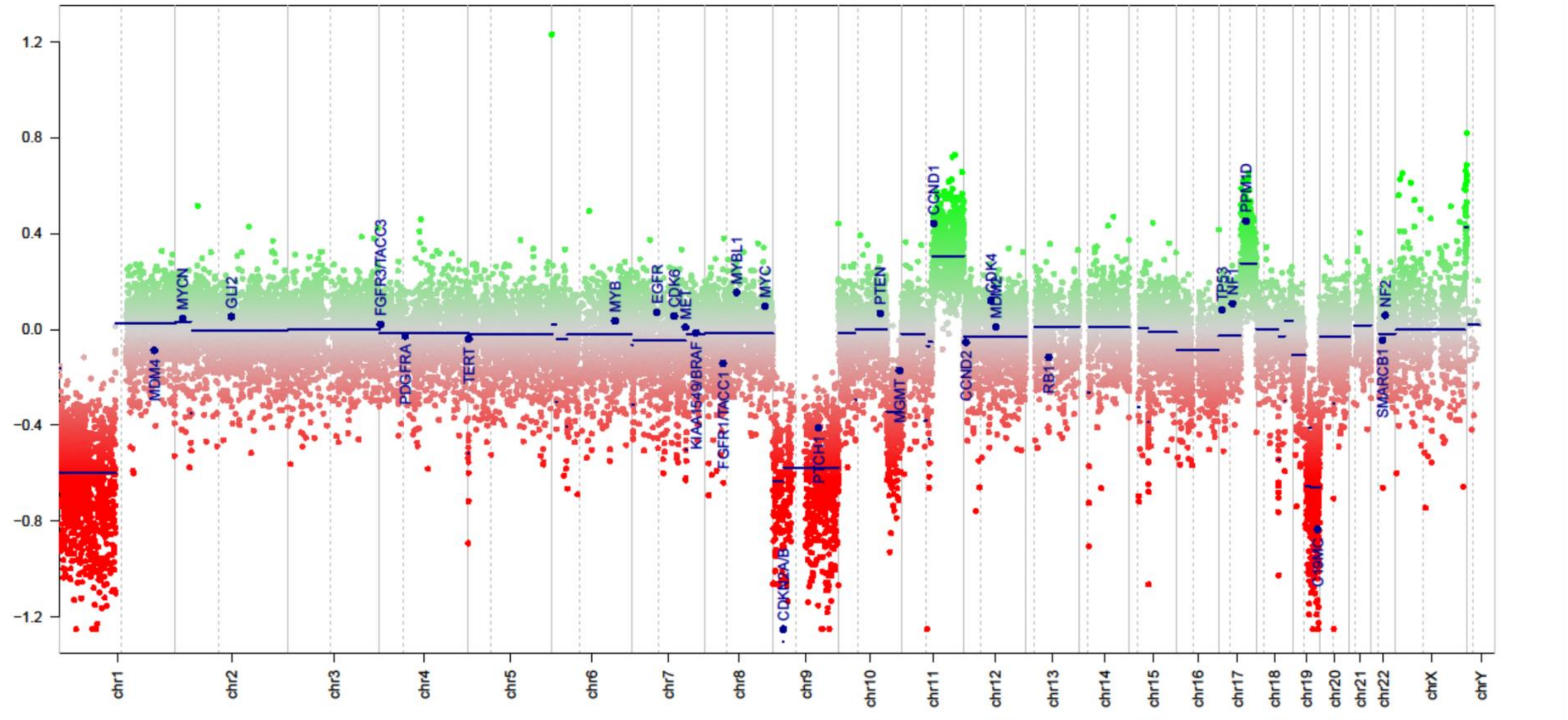

1.2
0.8
0.4
0.0
-0.4
-0.8
-1.2
MYCN
GLI2
FGFR3/TACC3
MYB
EGFR
CDK6
MET
MYBL1
MYC
PTEN
CCND1
CDK4
MDM2
TP53
NF1
PPM1D
NF2
MDM4
PDGFRA
TERT
KIAA1549/BRAF
FGFR1/TACC1
CDKN2A/B
PTCH1
MGMT
CCND2
RB1
SMARCB1
chr1
chr2
chr3
chr4
chr5
chr6
chr7
chr8
chr9
chr10
chr11
chr12
chr13
chr14
chr15
chr16
chr17
chr18
chr19
chr20
chr21
chr22
chrX
chrY

HG-36

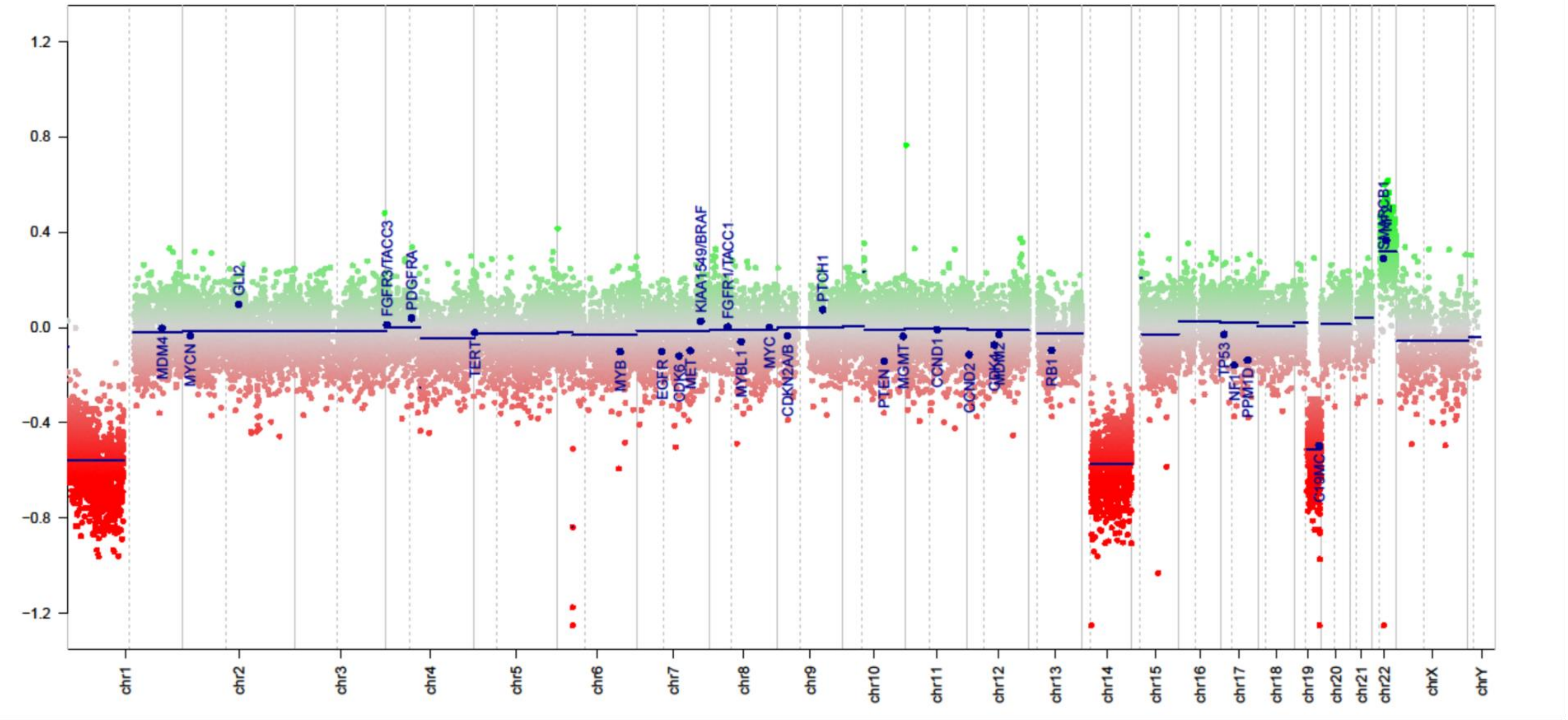
1.2
0.8
0.4
0.0
-0.4
-0.8
-1.2
chr1
chr2
chr3
chr4
chr5
chr6
chr7
chr8
chr9
chr10
chr11
chr12
chr13
chr14
chr15
chr16
chr17
chr18
chr19
chr20
chr21
chr22
chrX
chrY
MDM4
MYCN
GLI2
FGFR3/TACC3
PDGFRA
TERT
MYB
EGFR
CDK6
MET
KIAA1549/BRAF
FGFR1/TACC1
MYBL1
MYC
CDKN2A/B
PTCH1
PTEN
MGMT
CCND1
CCND2
CDK4
MDM2
RB1
TP53
NF1
PPM1D

HG-37

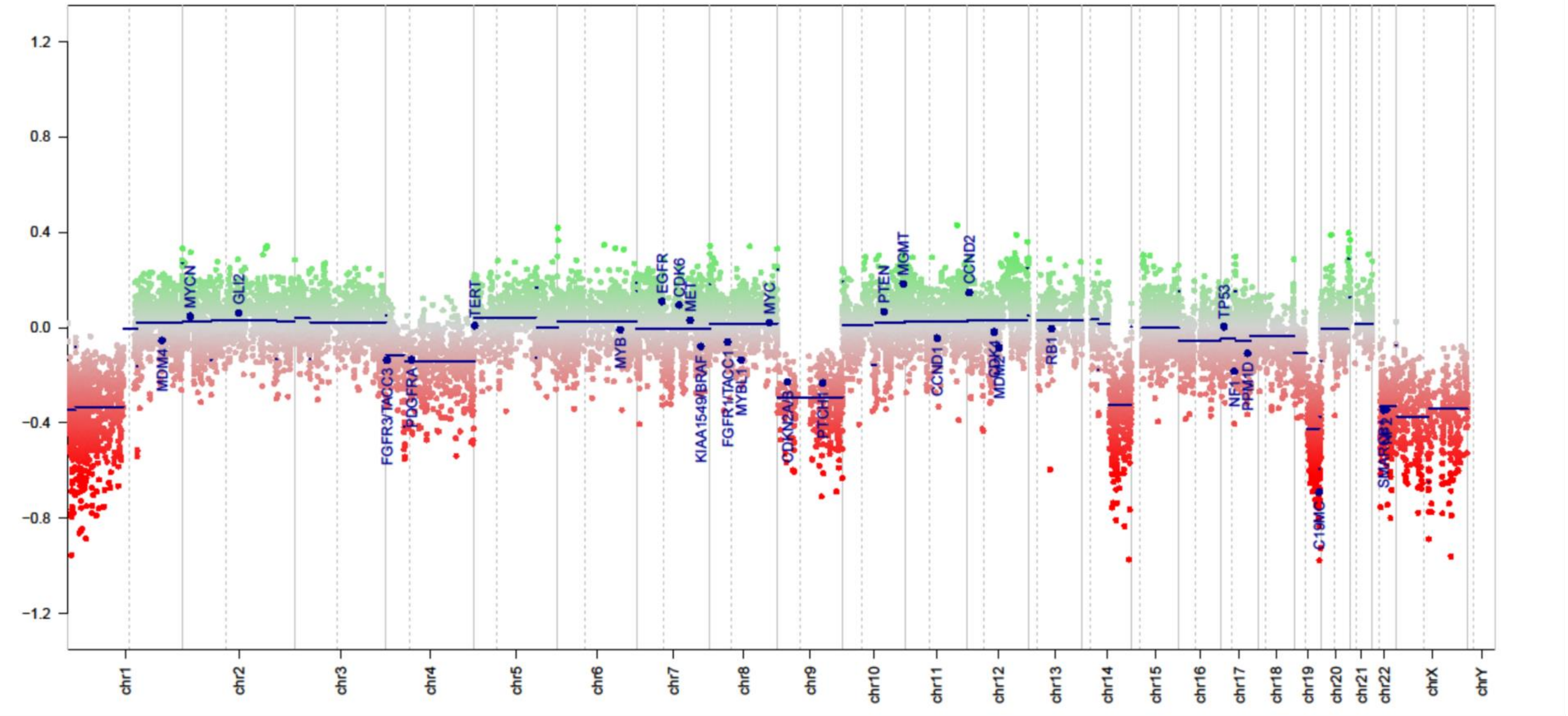

1.2
0.8
0.4
0.0
-0.4
-0.8
-1.2
chr1
chr2
chr3
chr4
chr5
chr6
chr7
chr8
chr9
chr10
chr11
chr12
chr13
chr14
chr15
chr16
chr17
chr18
chr19
chr20
chr21
chr22
chrX
chrY
MDM4
MYCN
GLI2
FGFR3/TACC3
PDGFRA
TERT
MYB
EGFR
CDK6
MET
KIAA1549/BRAF
FGFR1/TACC1
MYBL1
MYC
CDKN2A/B
PTCH1
PTEN
MGMT
CCND1
CCND2
CDK4
MDM2
RB1
TP53
NF1
PPM1D

HG-38

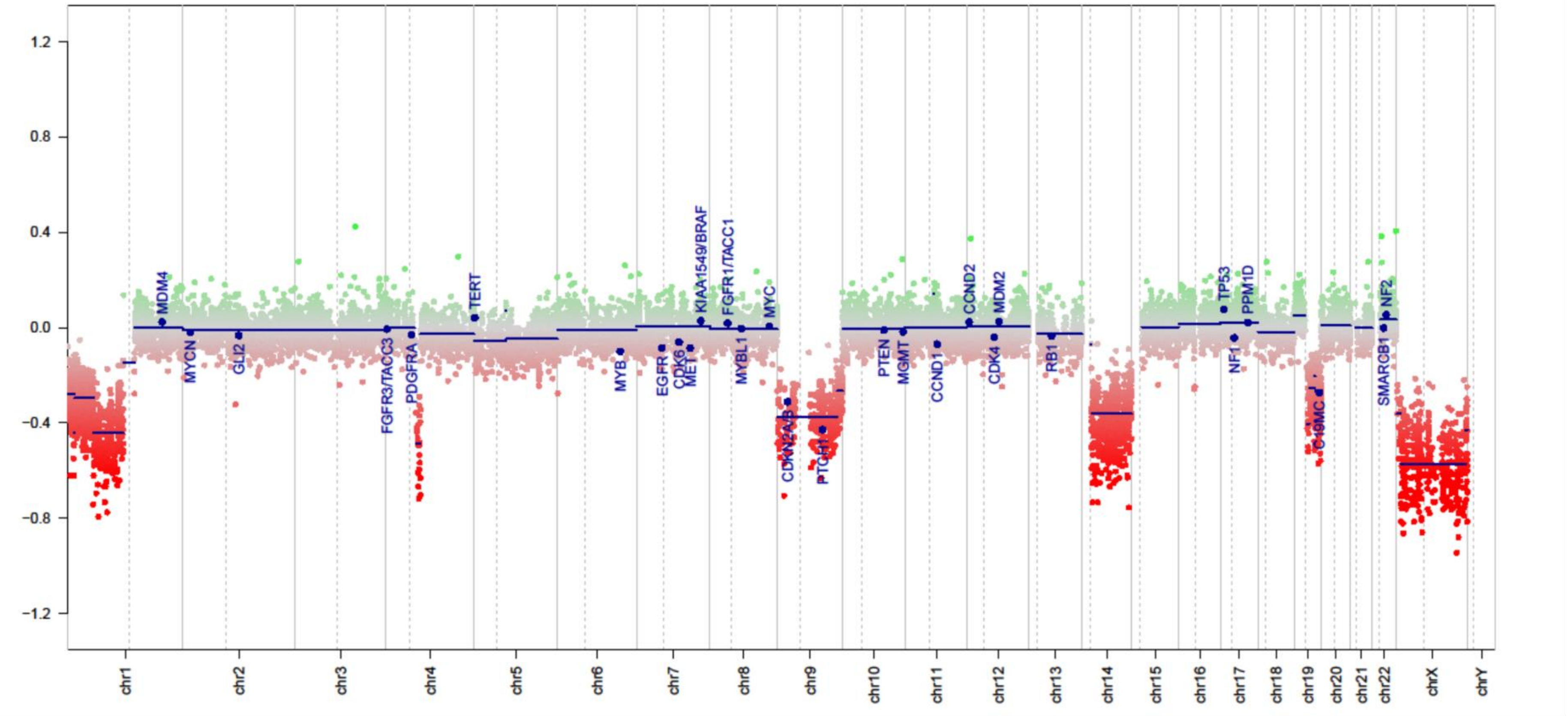
1.2
0.8
0.4
0.0
-0.4
-0.8
-1.2
MDM4
MYCN
GLI2
FGFR3/TACC3
PDGFRA
TERT
MYB
EGFR
CDK6
MET
KIAA1549/BRAF
FGFR1/TACC1
MYBL1
MYC
CDKN2A/B
PTCH1
PTEN
MGMT
CCND1
CCND2
CDK4
MDM2
RB1
TP53
NF1
PPM1D
C19MC
SMARCB1
NF2
chr1
chr2
chr3
chr4
chr5
chr6
chr7
chr8
chr9
chr10
chr11
chr12
chr13
chr14
chr15
chr16
chr17
chr18
chr19
chr20
chr21
chr22
chrX
chrY

HG-39

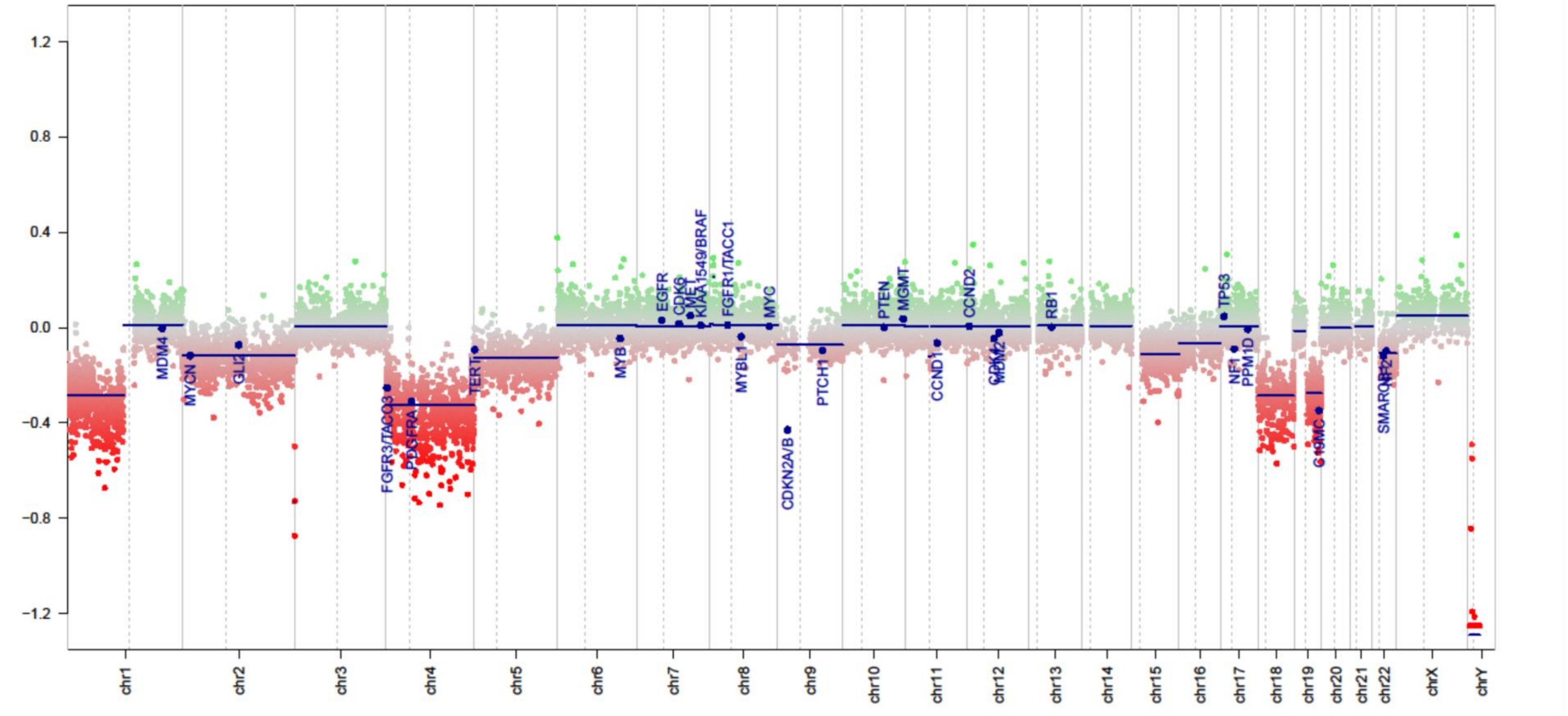
1.2
0.8
0.4
0.0
-0.4
-0.8
-1.2
MDM4
MYCN
GLI2
FGFR3/TACC3
PDGFRA
TERT
MYB
EGFR
CDK6
MET
KIAA1549/BRAF
FGFR1/TACC1
MYBL1
MYC
CDKN2A/B
PTCH1
PTEN
MGMT
CCND1
CCND2
CDK4
MDM2
RB1
TP53
NF1
PPM1D
SMARCB1
chr1
chr2
chr3
chr4
chr5
chr6
chr7
chr8
chr9
chr10
chr11
chr12
chr13
chr14
chr15
chr16
chr17
chr18
chr19
chr20
chr21
chr22
chrX
chrY

HG-40

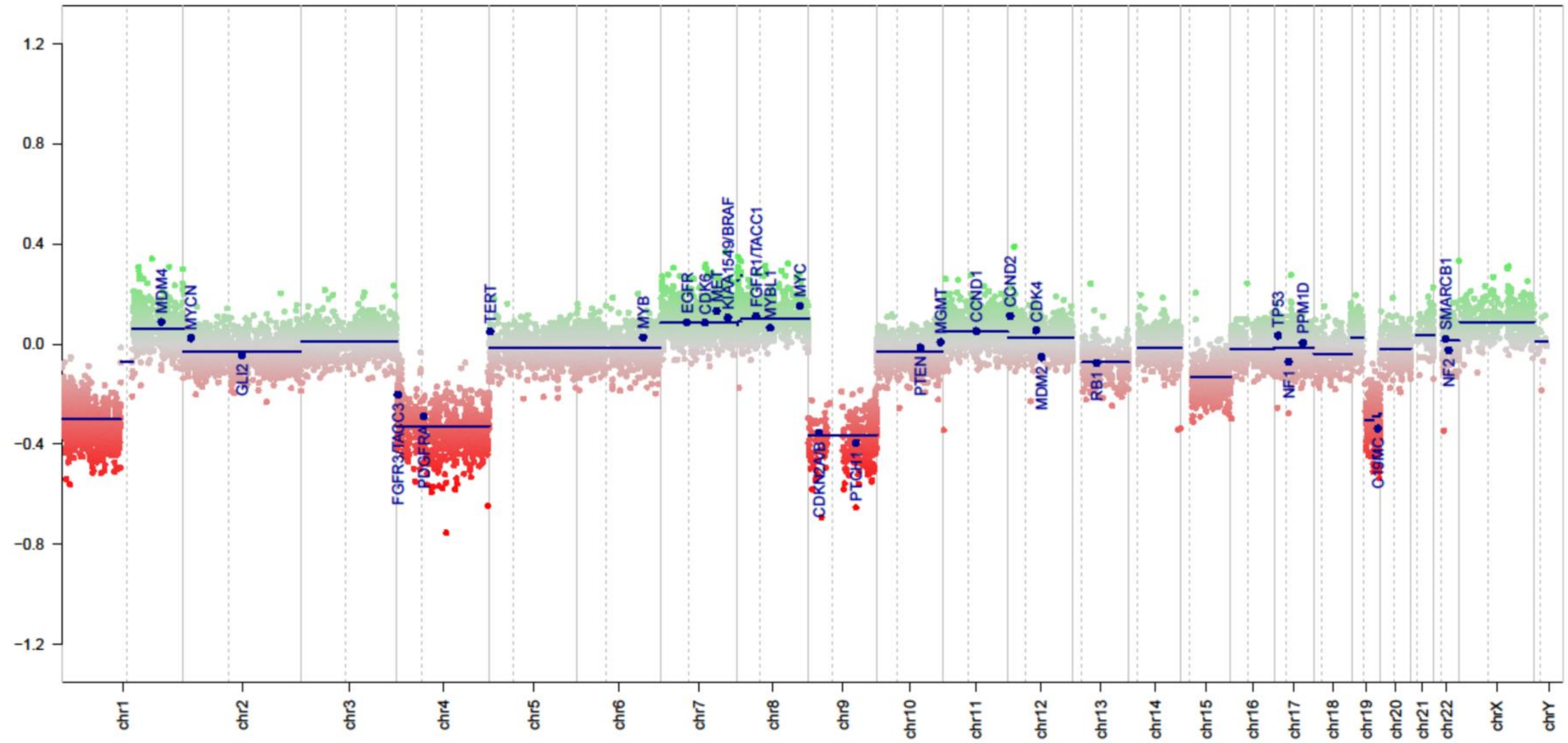

1.2
0.8
0.4
0.0
−0.4
−0.8
−1.2
MDM4
MYCN
GLI2
FGFR3/TACC3
PDGFRA
TERT
MYB
EGFR
CDK6
MET
KIAA1549/BRAF
FGFR1/TACC1
MYBL1
MYC
CDKN2A/B
PTCH1
PTEN
MGMT
CCND1
CCND2
CDK4
MDM2
RB1
TP53
NF1
PPM1D
SMARCB1
NF2
chr1
chr2
chr3
chr4
chr5
chr6
chr7
chr8
chr9
chr10
chr11
chr12
chr13
chr14
chr15
chr16
chr17
chr18
chr19
chr20
chr21
chr22
chrX
chrY

HG-41

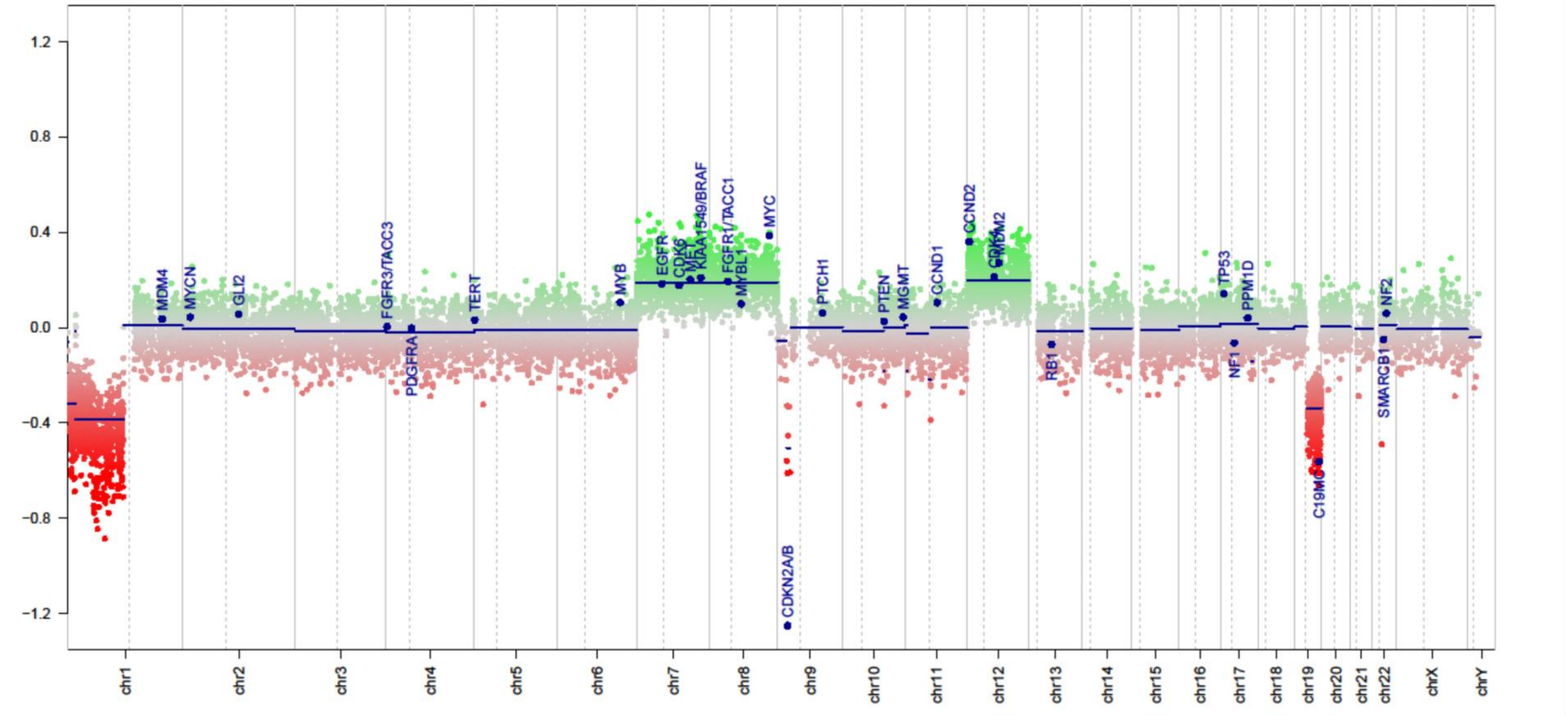
1.2
0.8
0.4
0.0
−0.4
−0.8
−1.2
MDM4
MYCN
GLI2
FGFR3/TACC3
PDGFRA
TERT
MYB
EGFR
CDK6
MET
KIAA1549/BRAF
FGFR1/TACC1
MYBL1
MYC
CDKN2A/B
PTCH1
PTEN
MGMT
CCND1
CCND2
CDK4
MDM2
RB1
TP53
PPM1D
NF1
C19MC
NF2
SMARCB1
chr1
chr2
chr3
chr4
chr5
chr6
chr7
chr8
chr9
chr10
chr11
chr12
chr13
chr14
chr15
chr16
chr17
chr18
chr19
chr20
chr21
chr22
chrX
chrY

HG-42

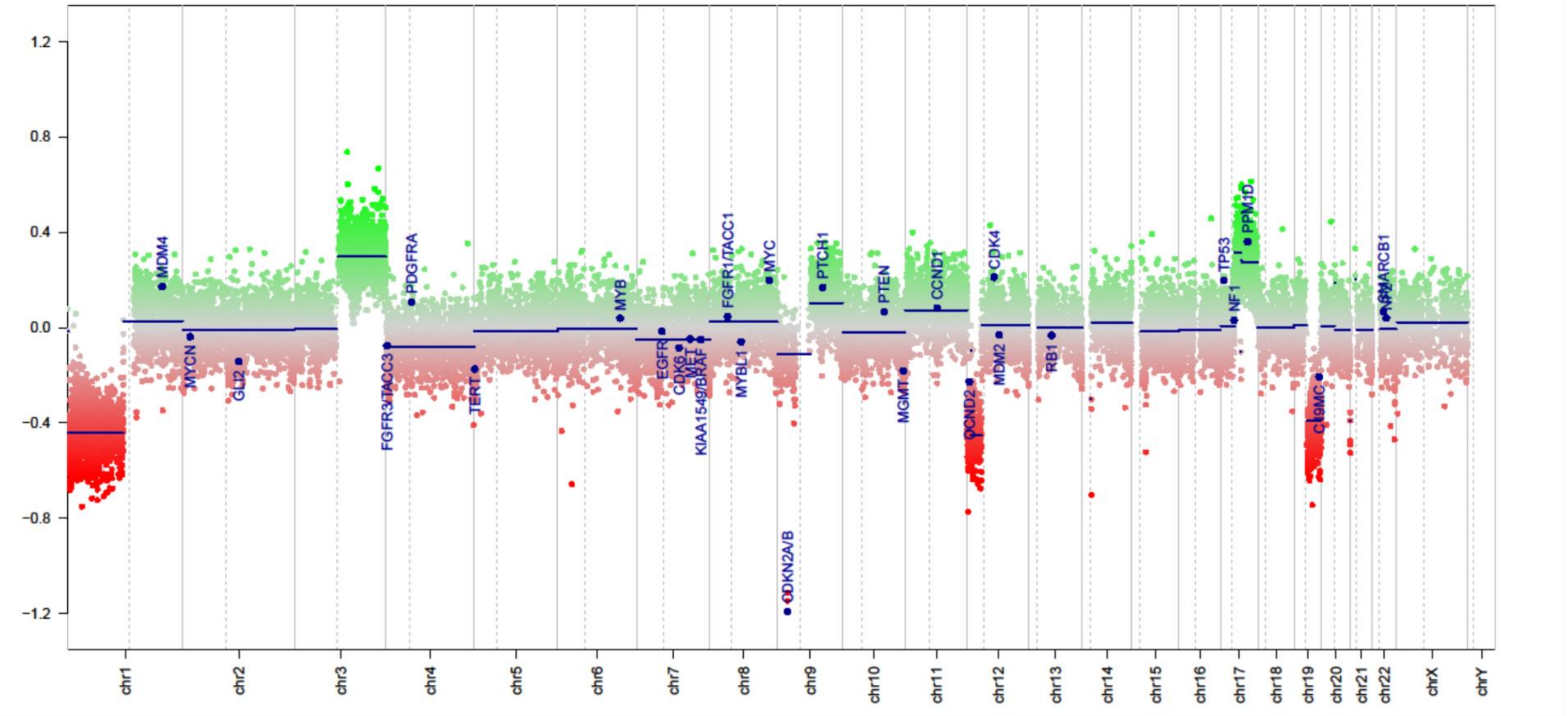

1.2
0.8
0.4
0.0
−0.4
−0.8
−1.2
MDM4
MYCN
GLI2
FGFR3/TACC3
PDGFRA
TERT
MYB
EGFR
CDK6
MET
KIAA1549/BRAF
FGFR1/TACC1
MYBL1
MYC
CDKN2A/B
PTCH1
PTEN
MGMT
CCND1
CCND2
CDK4
MDM2
RB1
TP53
NF1
PPM1D
C19MC
SMARCB1
NF2
chr1
chr2
chr3
chr4
chr5
chr6
chr7
chr8
chr9
chr10
chr11
chr12
chr13
chr14
chr15
chr16
chr17
chr18
chr19
chr20
chr21
chr22
chrX
chrY

LG-01

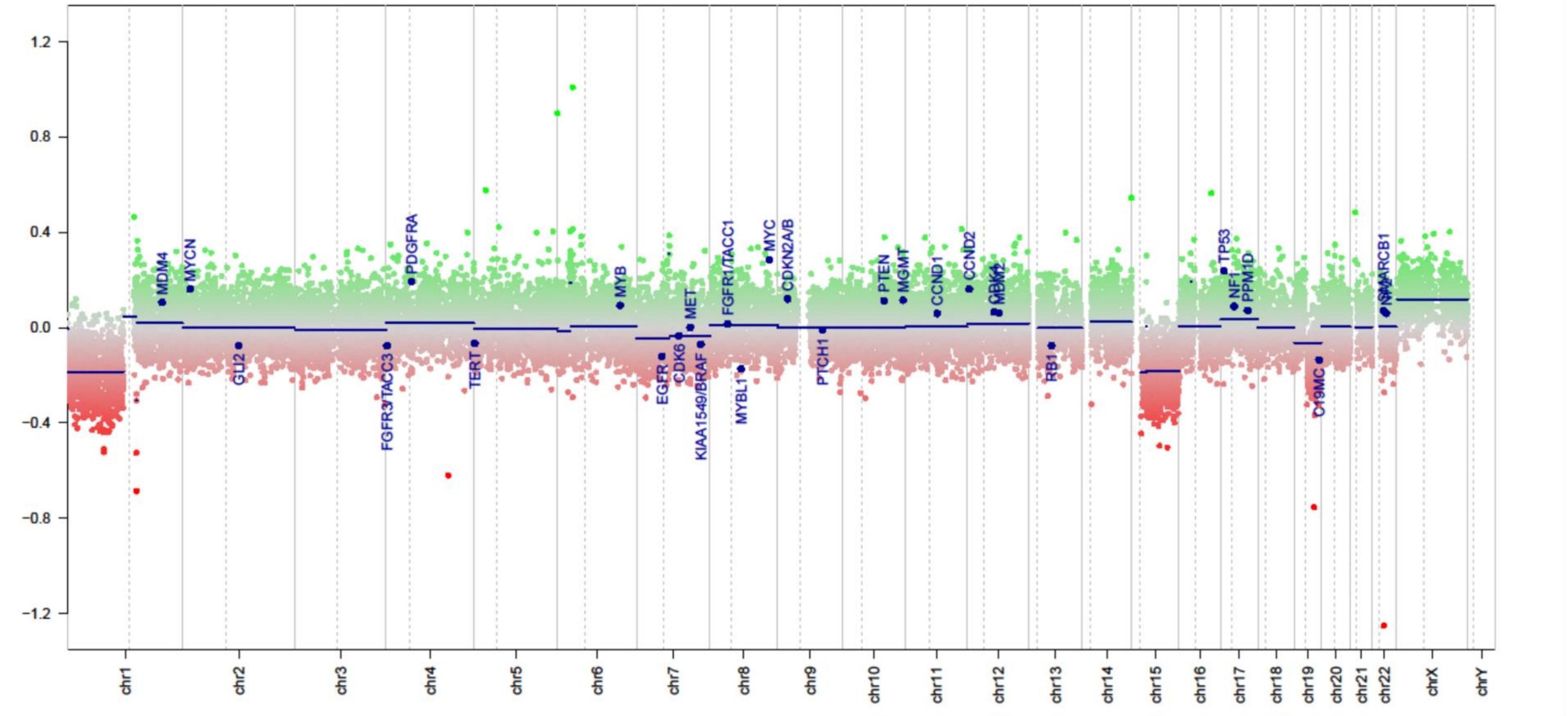

1.2
0.8
0.4
0.0
−0.4
−0.8
−1.2
MDM4
MYCN
GLI2
FGFR3/TACC3
PDGFRA
TERT
MYB
EGFR
CDK6
MET
KIAA1549/BRAF
FGFR1/TACC1
MYBL1
MYC
CDKN2A/B
PTCH1
PTEN
MGMT
CCND1
CCND2
CDK4
MDM2
RB1
TP53
NF1
PPM1D
C19MC
SMARCB1
chr1
chr2
chr3
chr4
chr5
chr6
chr7
chr8
chr9
chr10
chr11
chr12
chr13
chr14
chr15
chr16
chr17
chr18
chr19
chr20
chr21
chr22
chrX
chrY

LG-02

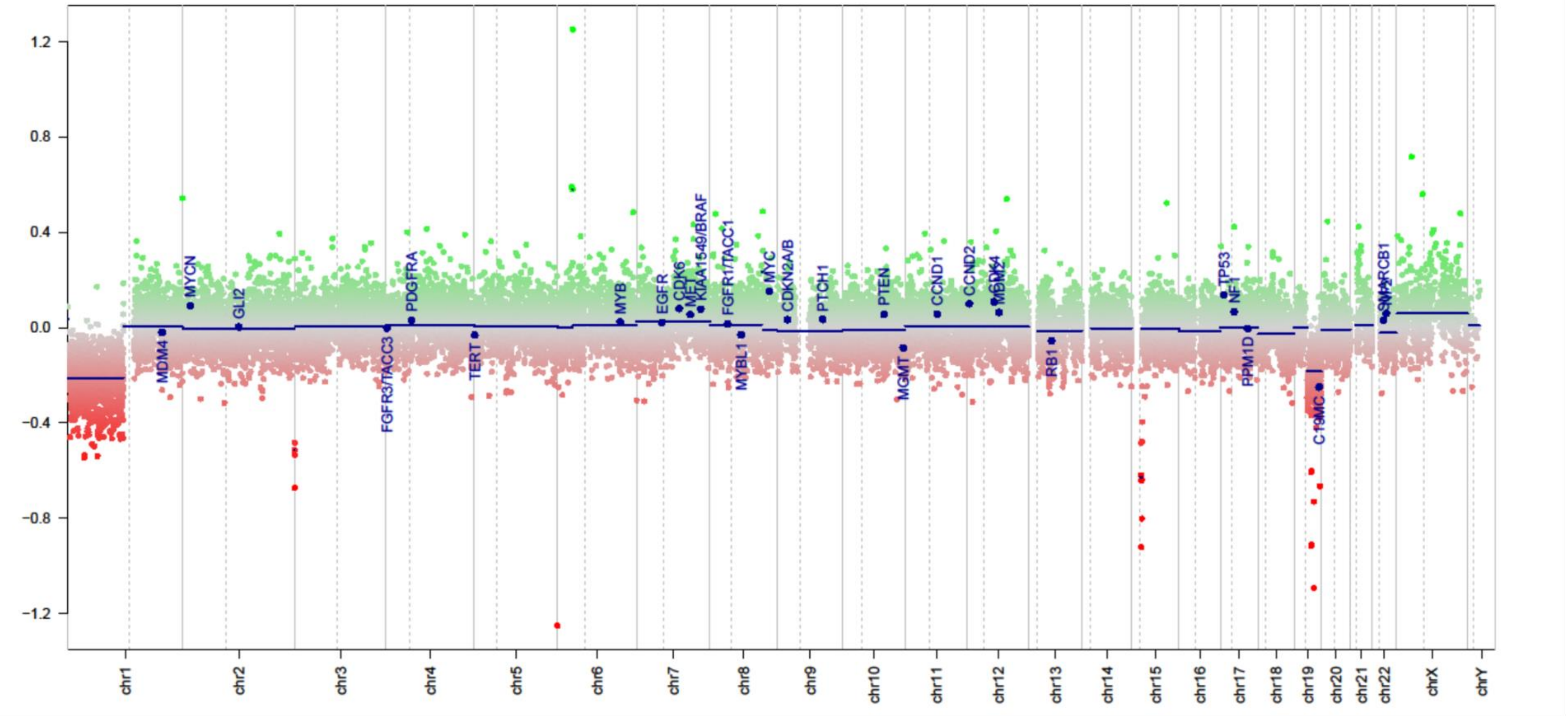

1.2
0.8
0.4
0.0
-0.4
-0.8
-1.2
MDM4
MYCN
GLI2
FGFR3/TACC3
PDGFRA
TERT
MYB
EGFR
CDK6
MET
KIAA1549/BRAF
FGFR1/TACC1
MYBL1
MYC
CDKN2A/B
PTCH1
PTEN
MGMT
CCND1
CCND2
CDK4
MDM2
RB1
TP53
NF1
PPM1D
C19MC
SMARCB1
chr1
chr2
chr3
chr4
chr5
chr6
chr7
chr8
chr9
chr10
chr11
chr12
chr13
chr14
chr15
chr16
chr17
chr18
chr19
chr20
chr21
chr22
chrX
chrY

LG-03

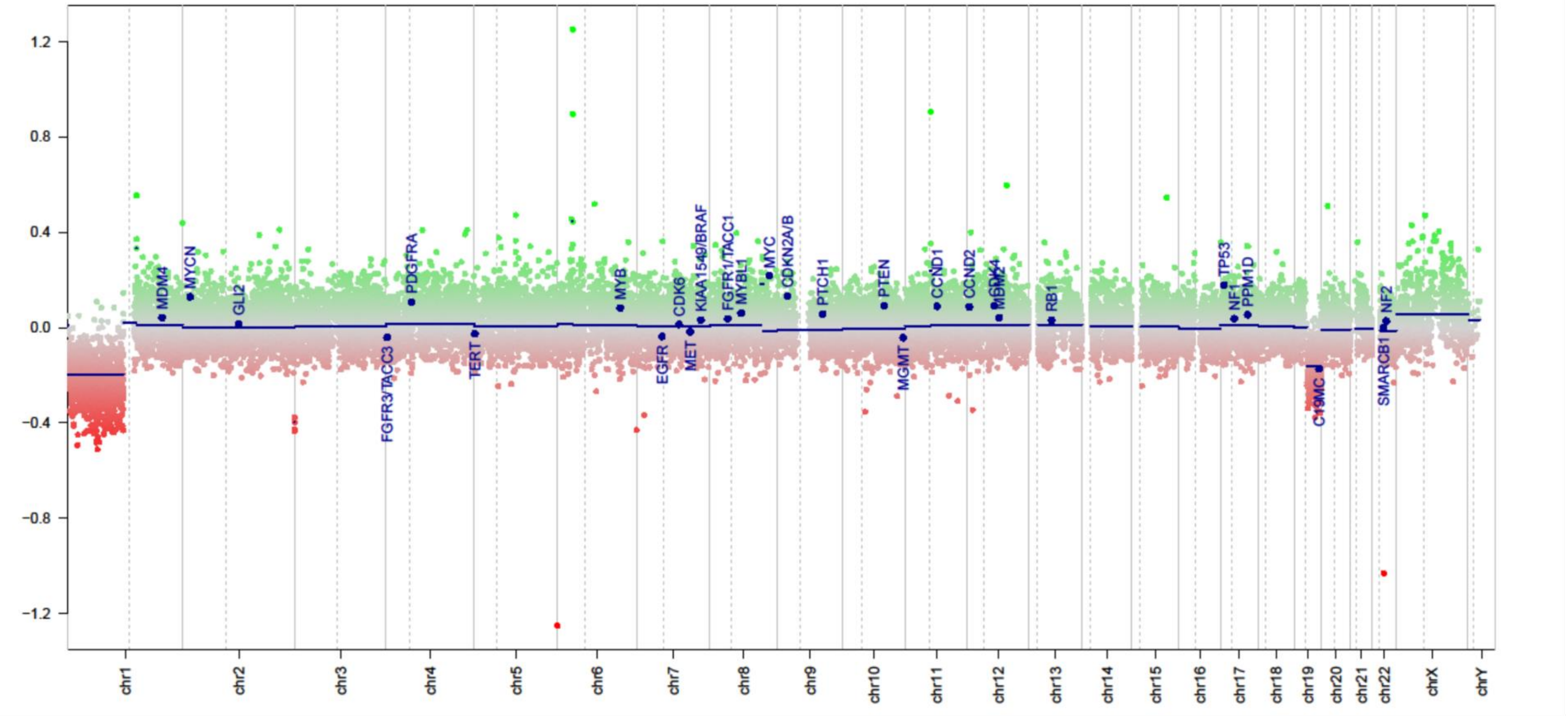

1.2
0.8
0.4
0.0
-0.4
-0.8
-1.2
MDM4
MYCN
GLI2
FGFR3/TACC3
PDGFRA
TERT
MYB
EGFR
CDK6
MET
KIAA1549/BRAF
FGFR1/TACC1
MYBL1
MYC
CDKN2A/B
PTCH1
PTEN
MGMT
CCND1
CCND2
CDK4
MDM2
RB1
TP53
NF1
PPM1D
C19MC
SMARCB1
NF2
chr1
chr2
chr3
chr4
chr5
chr6
chr7
chr8
chr9
chr10
chr11
chr12
chr13
chr14
chr15
chr16
chr17
chr18
chr19
chr20
chr21
chr22
chrX
chrY

LG-04

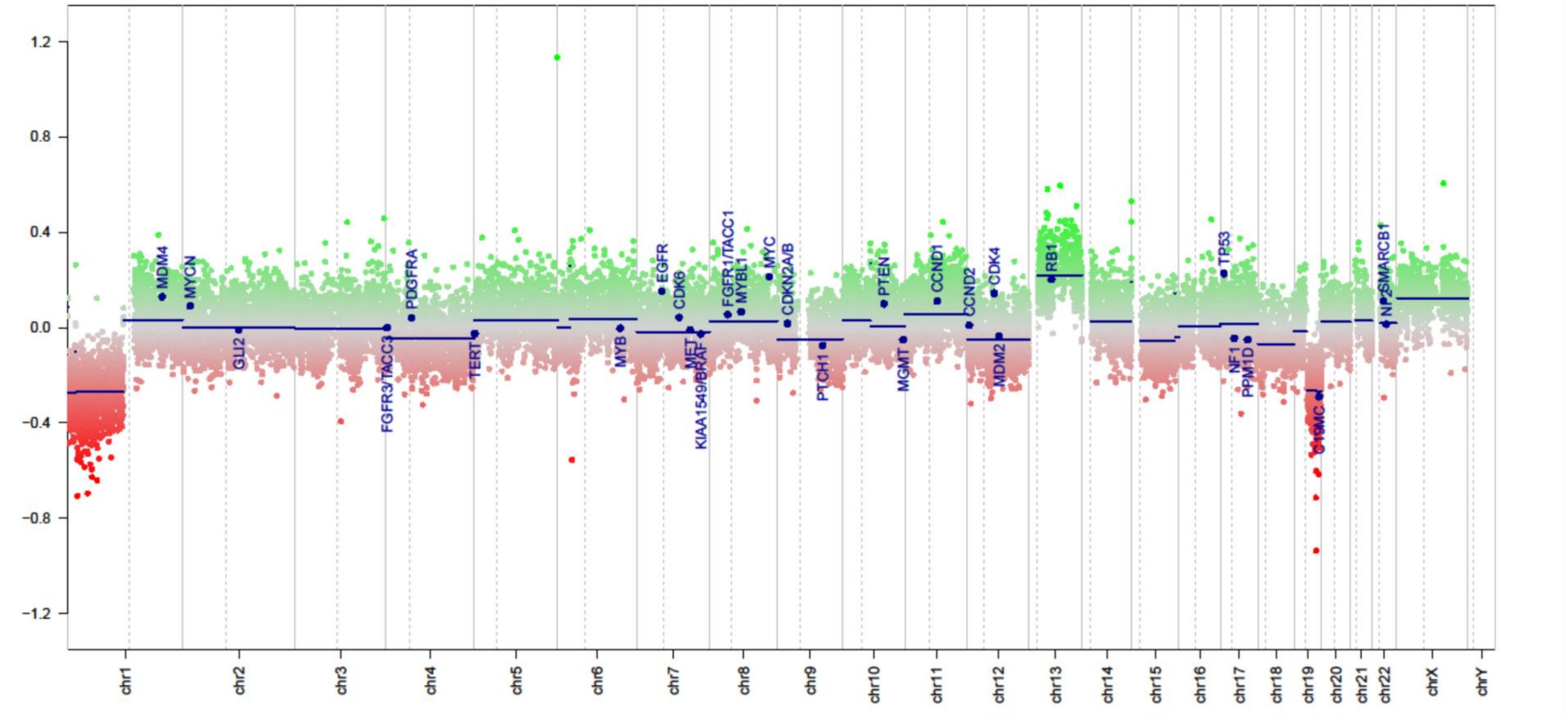
1.2
0.8
0.4
0.0
−0.4
−0.8
−1.2
MDM4
MYCN
GLI2
FGFR3/TACC3
PDGFRA
TERT
MYB
EGFR
CDK6
MET
KIAA1549/BRAF
FGFR1/TACC1
MYBL1
MYC
CDKN2A/B
PTCH1
PTEN
MGMT
CCND1
CCND2
CDK4
MDM2
RB1
TP53
NF1
PPM1D
C19MC
NF2
SMARCB1
chr1
chr2
chr3
chr4
chr5
chr6
chr7
chr8
chr9
chr10
chr11
chr12
chr13
chr14
chr15
chr16
chr17
chr18
chr19
chr20
chr21
chr22
chrX
chrY

LG-05

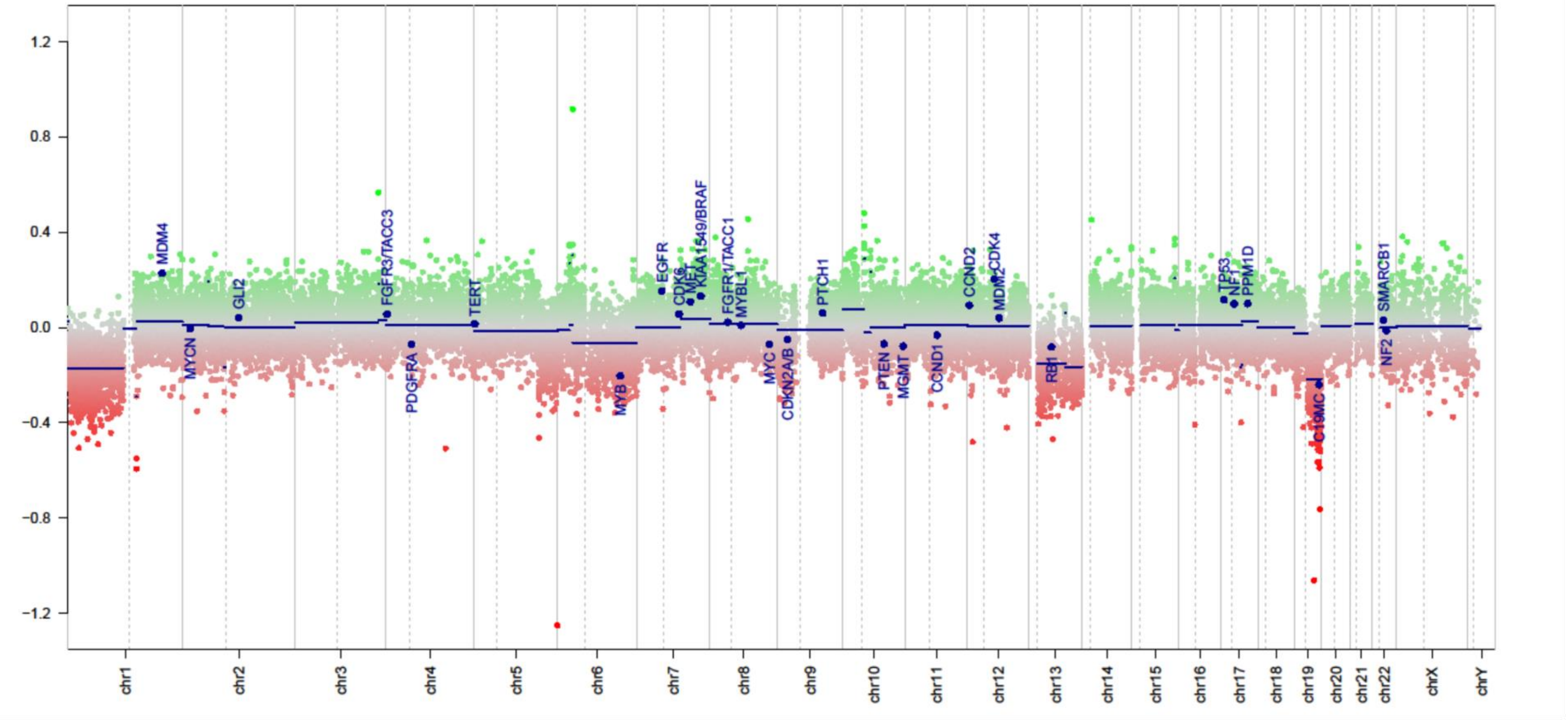

1.2
0.8
0.4
0.0
−0.4
−0.8
−1.2
MDM4
MYCN
GLI2
FGFR3/TACC3
PDGFRA
TERT
MYB
EGFR
CDK6
MET
KIAA1549/BRAF
FGFR1/TACC1
MYBL1
MYC
CDKN2A/B
PTCH1
PTEN
MGMT
CCND1
CCND2
MDM2
CDK4
RB1
TP53
NF1
PPM1D
C19MC
SMARCB1
NF2
chr1
chr2
chr3
chr4
chr5
chr6
chr7
chr8
chr9
chr10
chr11
chr12
chr13
chr14
chr15
chr16
chr17
chr18
chr19
chr20
chr21
chr22
chrX
chrY

LG-06

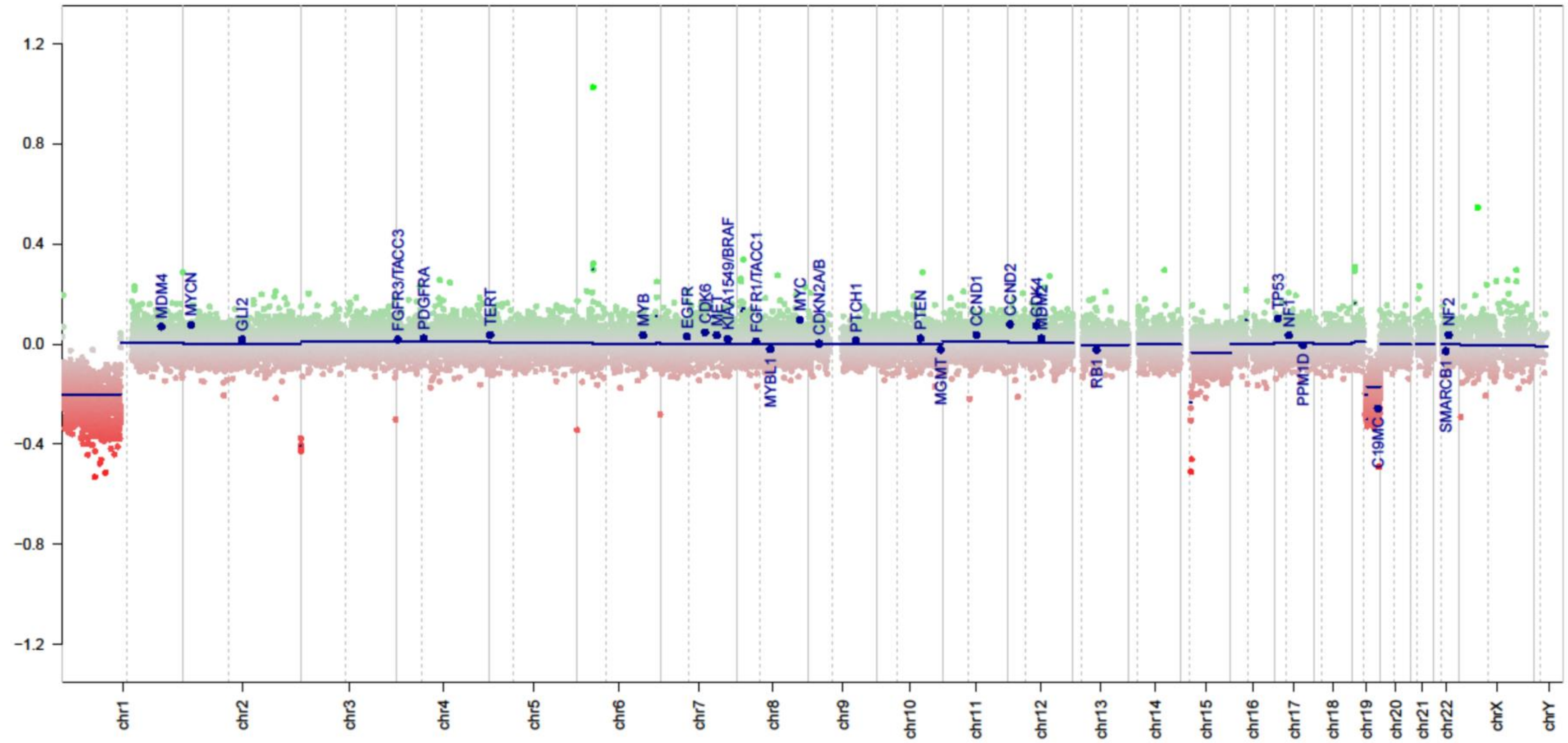

1.2
0.8
0.4
0.0
−0.4
−0.8
−1.2
MDM4
MYCN
GLI2
FGFR3/TACC3
PDGFRA
TERT
MYB
EGFR
CDK6
MET
KIAA1549/BRAF
FGFR1/TACC1
MYBL1
MYC
CDKN2A/B
PTCH1
PTEN
MGMT
CCND1
CCND2
CDK4
MDM2
RB1
TP53
NF1
PPM1D
C19MC
SMARCB1
NF2
chr1
chr2
chr3
chr4
chr5
chr6
chr7
chr8
chr9
chr10
chr11
chr12
chr13
chr14
chr15
chr16
chr17
chr18
chr19
chr20
chr21
chr22
chrX
chrY

LG-07

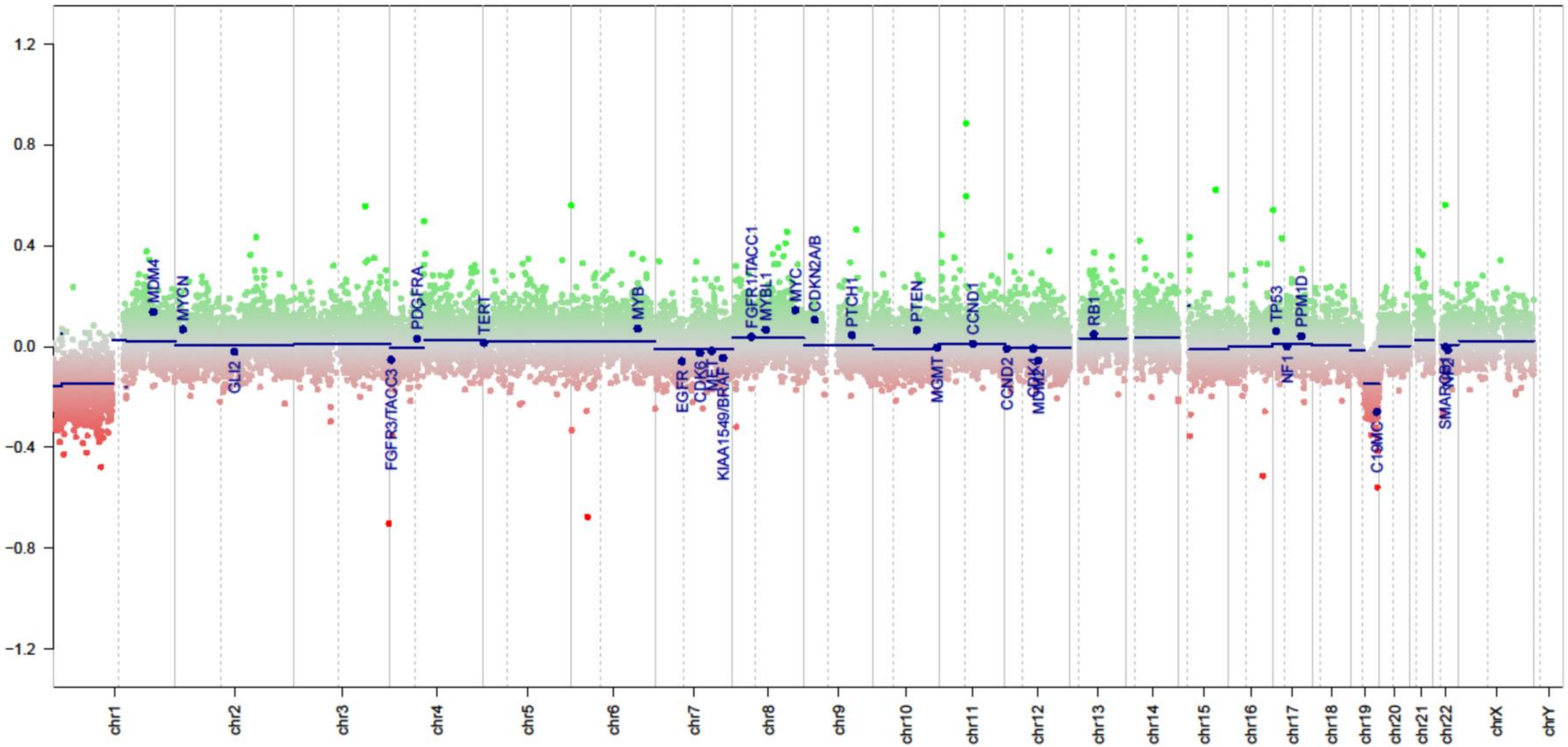

1.2
0.8
0.4
0.0
−0.4
−0.8
−1.2
MDM4
MYCN
GLI2
FGFR3/TACC3
PDGFRA
TERT
MYB
EGFR
CDK6
MET
KIAA1549/BRAF
FGFR1/TACC1
MYBL1
MYC
CDKN2A/B
PTCH1
PTEN
MGMT
CCND1
CCND2
CDK4
MDM2
RB1
TP53
NF1
PPM1D
C19MC
SMARCB1
chr1
chr2
chr3
chr4
chr5
chr6
chr7
chr8
chr9
chr10
chr11
chr12
chr13
chr14
chr15
chr16
chr17
chr18
chr19
chr20
chr21
chr22
chrX
chrY

LG-08

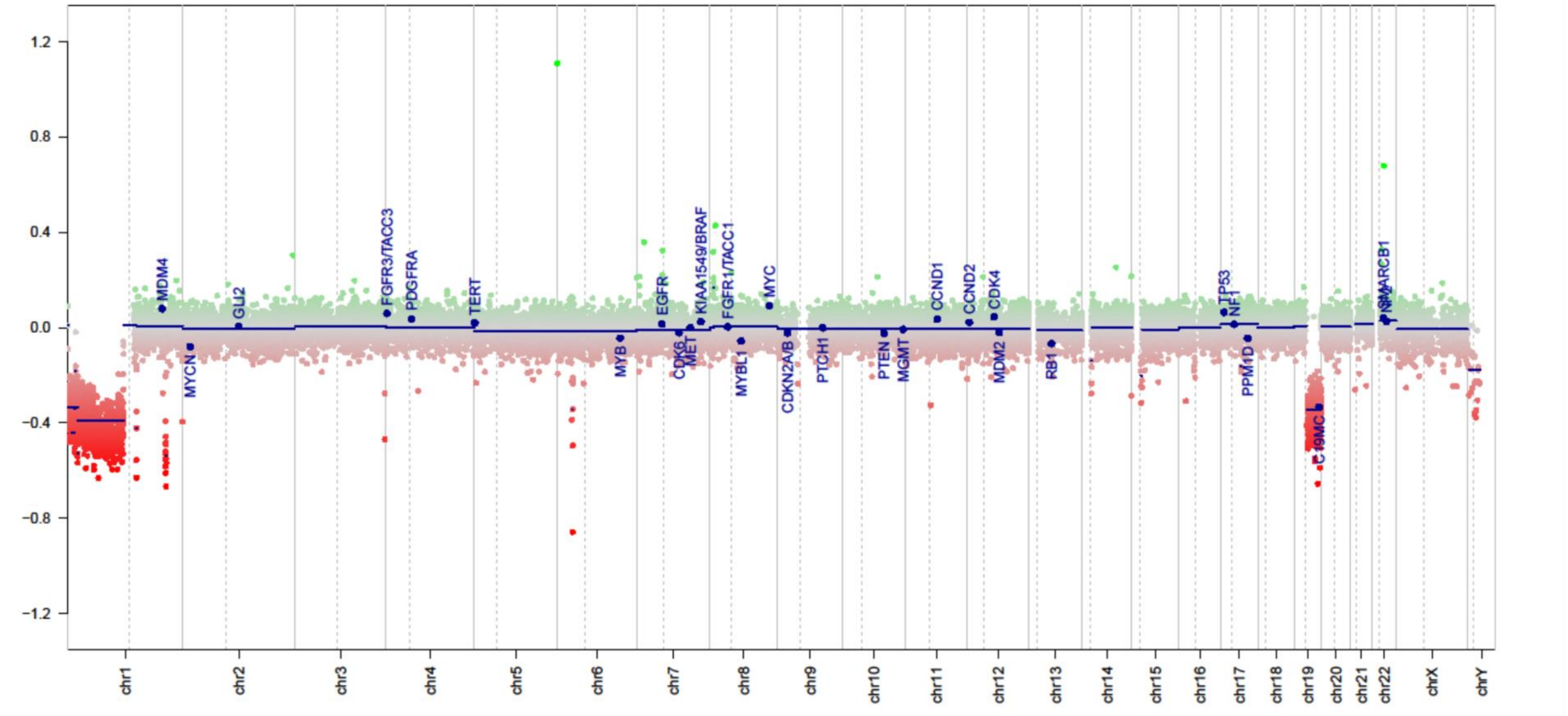

1.2
0.8
0.4
0.0
−0.4
−0.8
−1.2
MDM4
MYCN
GLI2
FGFR3/TACC3
PDGFRA
TERT
MYB
EGFR
CDK6
MET
KIAA1549/BRAF
FGFR1/TACC1
MYBL1
MYC
CDKN2A/B
PTCH1
PTEN
MGMT
CCND1
CCND2
CDK4
MDM2
RB1
TP53
NF1
PPM1D
C19MC
SMARCB1
chr1
chr2
chr3
chr4
chr5
chr6
chr7
chr8
chr9
chr10
chr11
chr12
chr13
chr14
chr15
chr16
chr17
chr18
chr19
chr20
chr21
chr22
chrX
chrY

LG-09

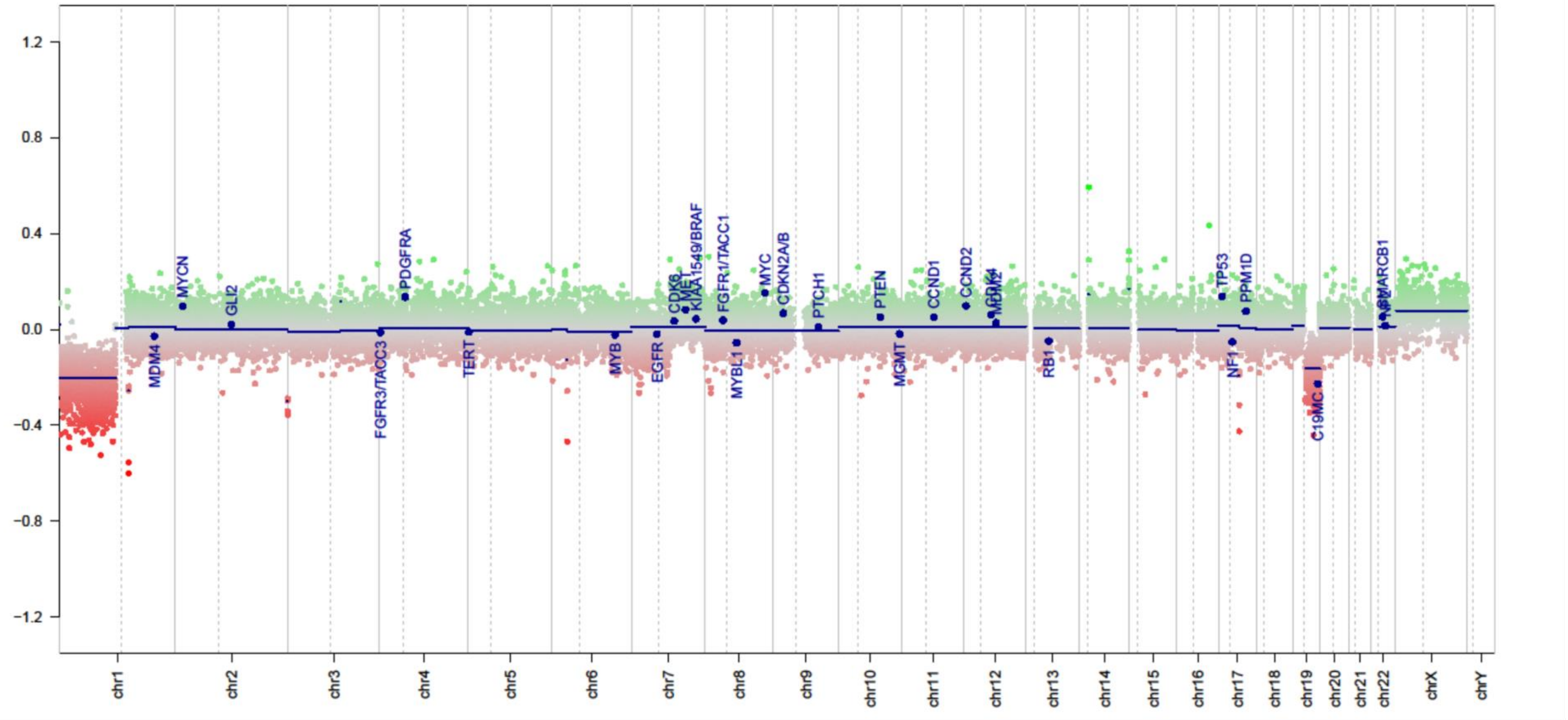

1.2
0.8
0.4
0.0
−0.4
−0.8
−1.2
MYCN
GLI2
PDGFRA
MDM4
FGFR3/TACC3
TERT
MYB
EGFR
CDK6
MET
KIAA1549/BRAF
FGFR1/TACC1
MYBL1
MYC
CDKN2A/B
PTCH1
PTEN
MGMT
CCND1
CCND2
CDK4
MDM2
RB1
TP53
PPM1D
NF1
C19MC
SMARCB1
chr1
chr2
chr3
chr4
chr5
chr6
chr7
chr8
chr9
chr10
chr11
chr12
chr13
chr14
chr15
chr16
chr17
chr18
chr19
chr20
chr21
chr22
chrX
chrY

LG-10

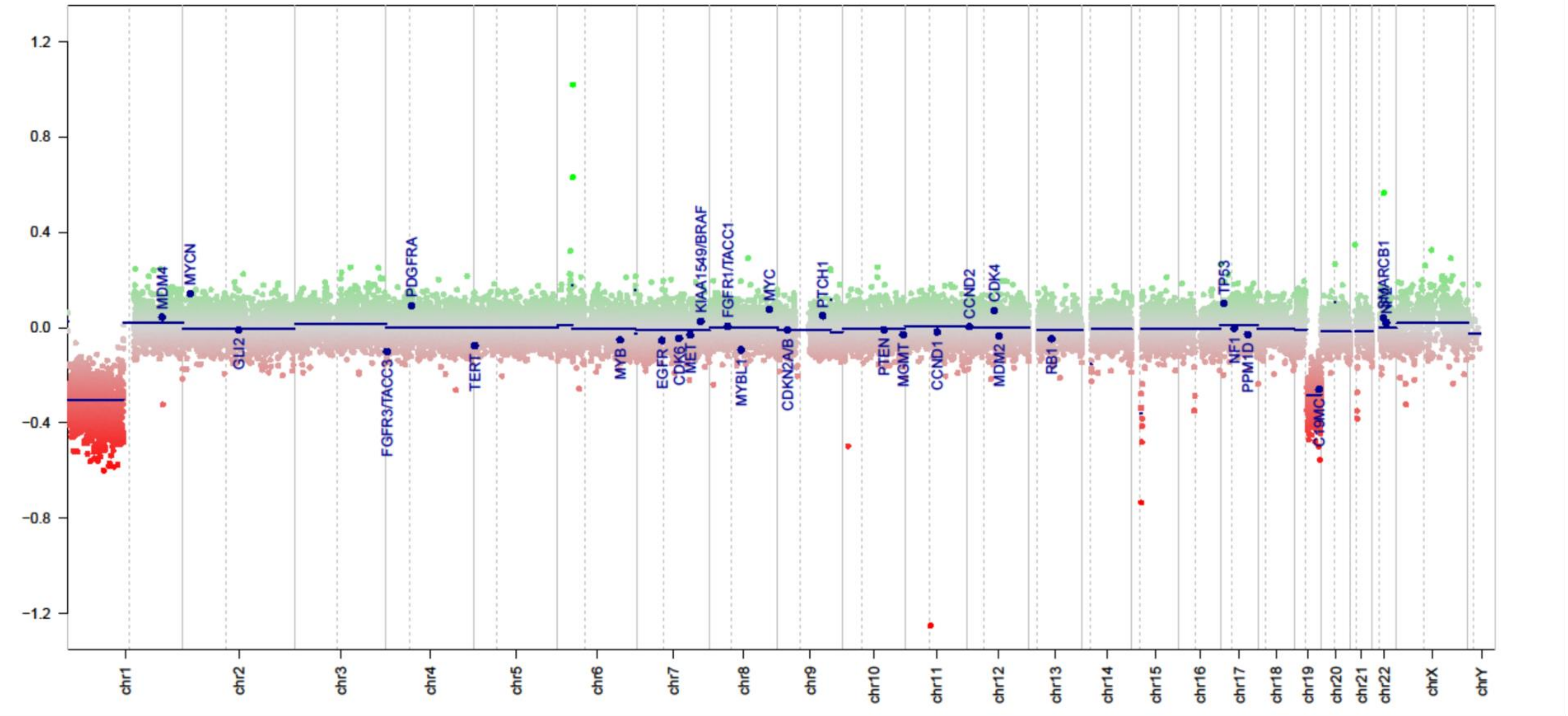

1.2
0.8
0.4
0.0
−0.4
−0.8
−1.2
MDM4
MYCN
GLI2
FGFR3/TACC3
PDGFRA
TERT
MYB
EGFR
CDK6
MET
KIAA1549/BRAF
FGFR1/TACC1
MYBL1
MYC
CDKN2A/B
PTCH1
PTEN
MGMT
CCND1
CCND2
CDK4
MDM2
RB1
TP53
NF1
PPM1D
C19MC
SMARCB1
chr1
chr2
chr3
chr4
chr5
chr6
chr7
chr8
chr9
chr10
chr11
chr12
chr13
chr14
chr15
chr16
chr17
chr18
chr19
chr20
chr21
chr22
chrX
chrY

LG-11

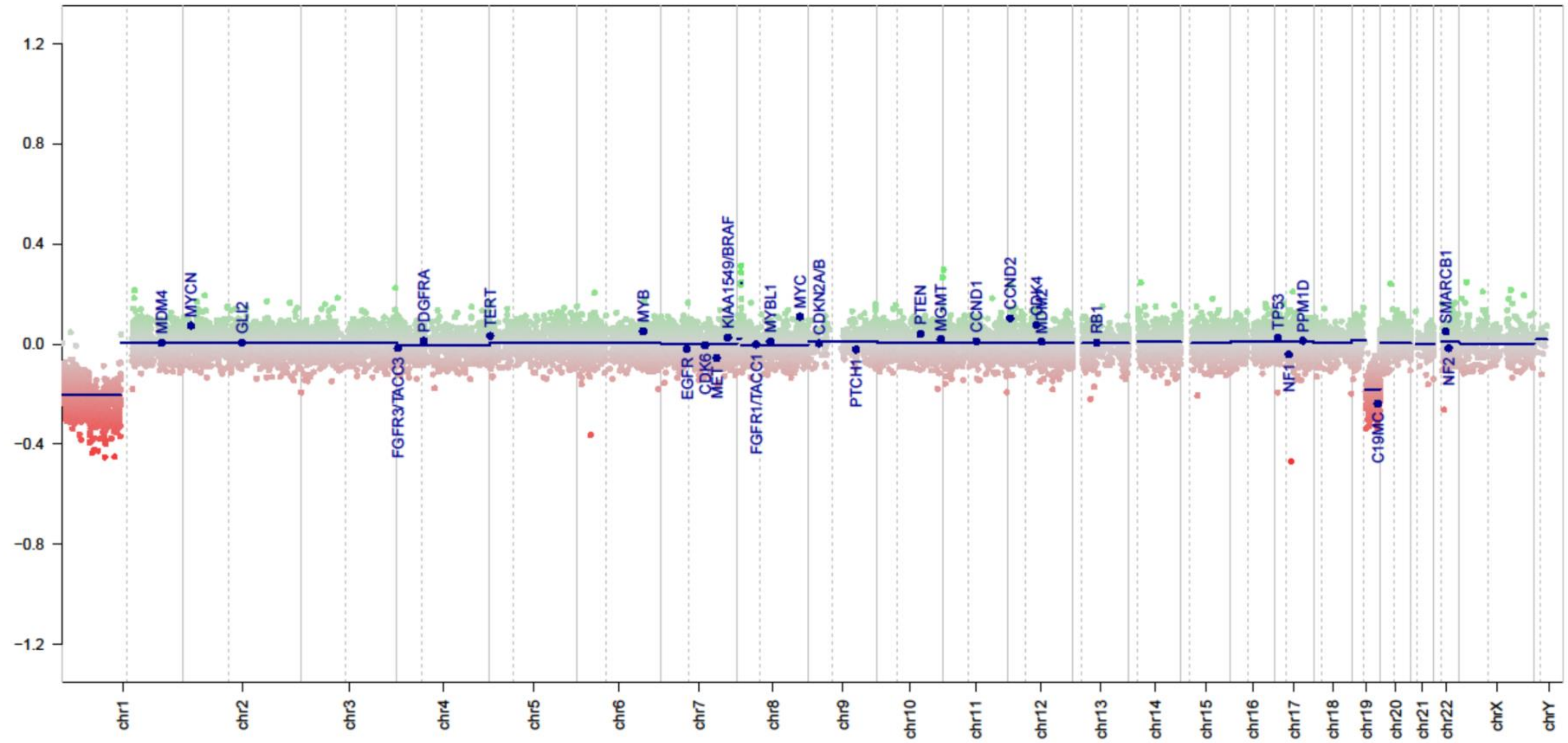

1.2
0.8
0.4
0.0
−0.4
−0.8
−1.2
MDM4
MYCN
GLI2
FGFR3/TACC3
PDGFRA
TERT
MYB
EGFR
CDK6
MET
KIAA1549/BRAF
FGFR1/TACC1
MYBL1
MYC
CDKN2A/B
PTCH1
PTEN
MGMT
CCND1
CCND2
CDK4
MDM2
RB1
TP53
NF1
PPM1D
C19MC
SMARCB1
NF2
chr1
chr2
chr3
chr4
chr5
chr6
chr7
chr8
chr9
chr10
chr11
chr12
chr13
chr14
chr15
chr16
chr17
chr18
chr19
chr20
chr21
chr22
chrX
chrY

LG-12

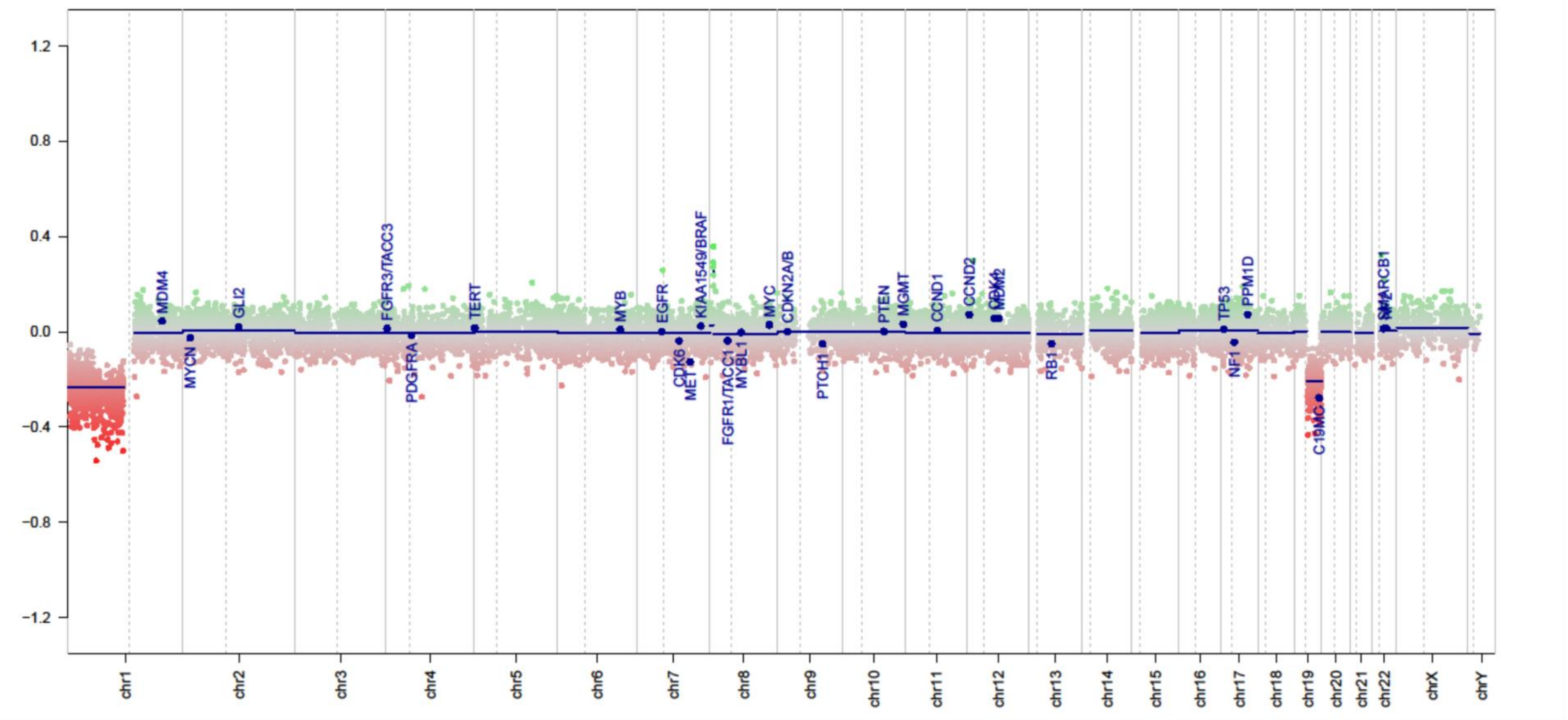

1.2
0.8
0.4
0.0
−0.4
−0.8
−1.2
chr1
chr2
chr3
chr4
chr5
chr6
chr7
chr8
chr9
chr10
chr11
chr12
chr13
chr14
chr15
chr16
chr17
chr18
chr19
chr20
chr21
chr22
chrX
chrY
MDM4
MYCN
GLI2
FGFR3/TACC3
PDGFRA
TERT
MYB
EGFR
CDK6
MET
KIAA1549/BRAF
FGFR1/TACC1
MYBL1
MYC
CDKN2A/B
PTCH1
PTEN
MGMT
CCND1
CCND2
MDM2
RB1
TP53
NF1
PPM1D
C19MC
SMARCB1

LG-13

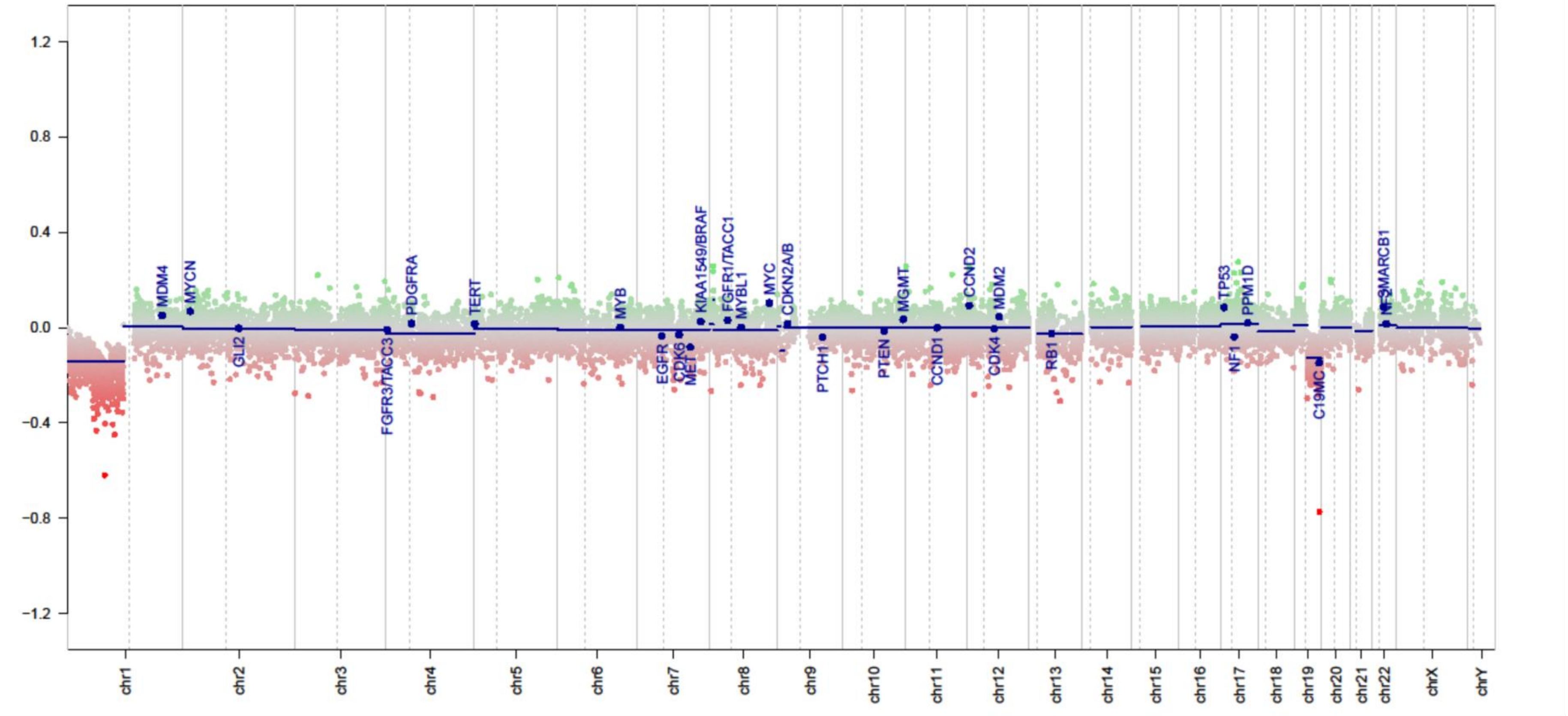

1.2
0.8
0.4
0.0
-0.4
-0.8
-1.2
MDM4
MYCN
GLI2
FGFR3/TACC3
PDGFRA
TERT
MYB
EGFR
CDK6
MET
KIAA1549/BRAF
FGFR1/TACC1
MYBL1
MYC
CDKN2A/B
PTCH1
PTEN
MGMT
CCND1
CCND2
CDK4
MDM2
RB1
TP53
NF1
PPM1D
C19MC
SMARCB1
chr1
chr2
chr3
chr4
chr5
chr6
chr7
chr8
chr9
chr10
chr11
chr12
chr13
chr14
chr15
chr16
chr17
chr18
chr19
chr20
chr21
chr22
chrX
chrY

LG-14

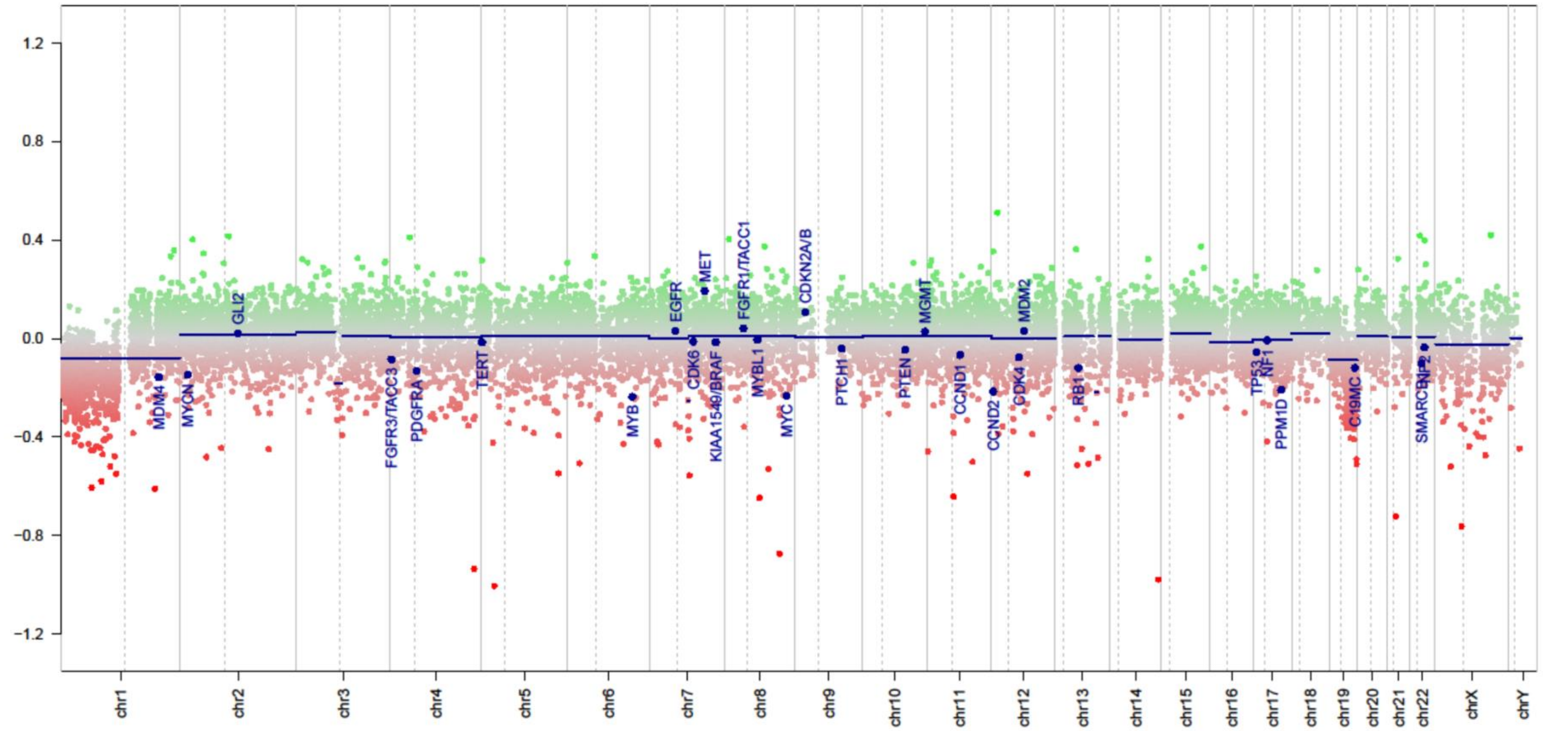
1.2
0.8
0.4
0.0
−0.4
−0.8
−1.2
MDM4
MYCN
GLI2
FGFR3/TACC3
PDGFRA
TERT
MYB
EGFR
CDK6
MET
KIAA1549/BRAF
FGFR1/TACC1
MYBL1
MYC
CDKN2A/B
PTCH1
PTEN
MGMT
CCND1
CCND2
CDK4
MDM2
RB1
TP53
NF1
PPM1D
C19MC
SMARCB1
chr1
chr2
chr3
chr4
chr5
chr6
chr7
chr8
chr9
chr10
chr11
chr12
chr13
chr14
chr15
chr16
chr17
chr18
chr19
chr20
chr21
chr22
chrX
chrY

LG-15

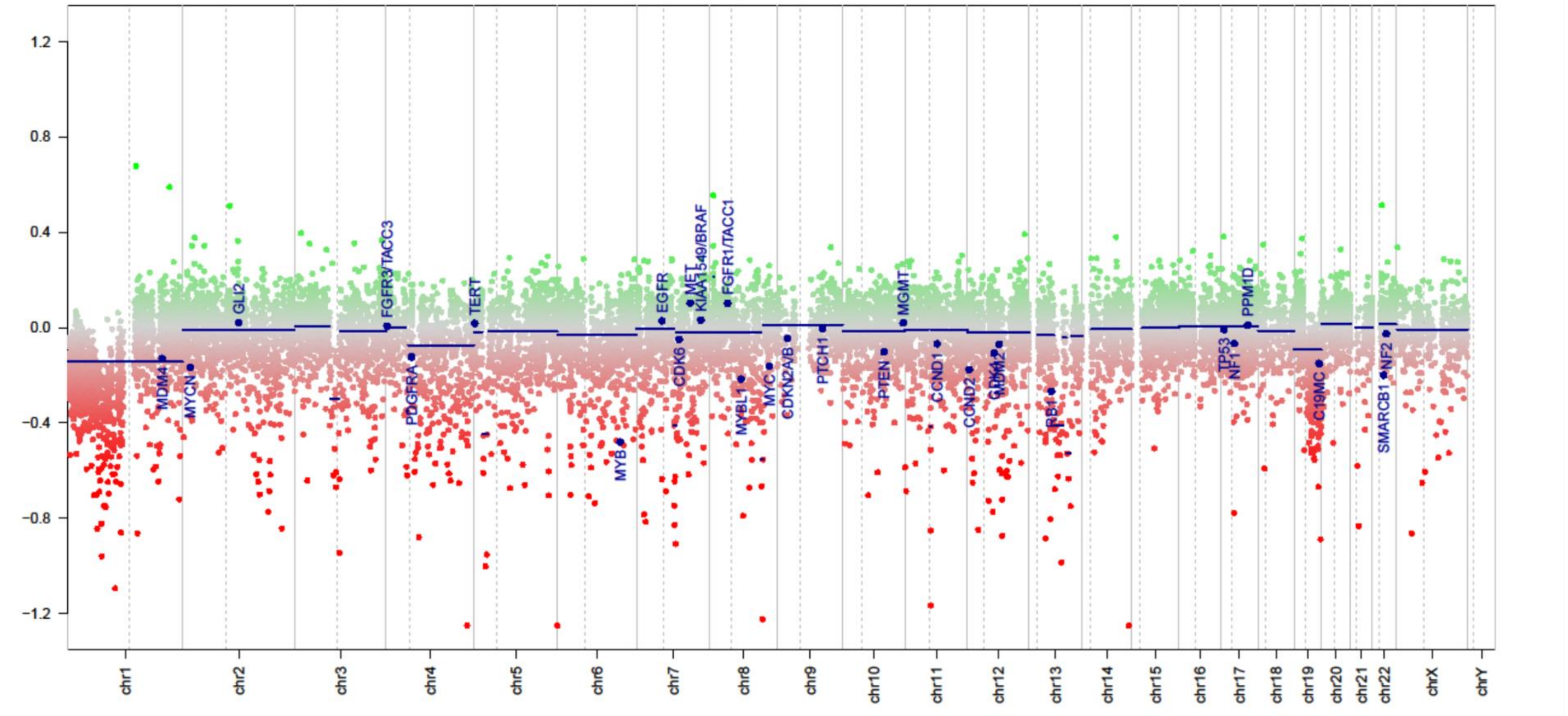
1.2
0.8
0.4
0.0
-0.4
-0.8
-1.2
MDM4
MYCN
GLI2
FGFR3/TACC3
PDGFRA
TERT
MYB
EGFR
CDK6
MET
KIAA1549/BRAF
FGFR1/TACC1
MYBL1
MYC
CDKN2A/B
PTCH1
PTEN
MGMT
CCND1
CCND2
CDK4
MDM2
RB1
TP53
NF1
PPM1D
C19MC
SMARCB1
NF2
chr1
chr2
chr3
chr4
chr5
chr6
chr7
chr8
chr9
chr10
chr11
chr12
chr13
chr14
chr15
chr16
chr17
chr18
chr19
chr20
chr21
chr22
chrX
chrY

LG-16

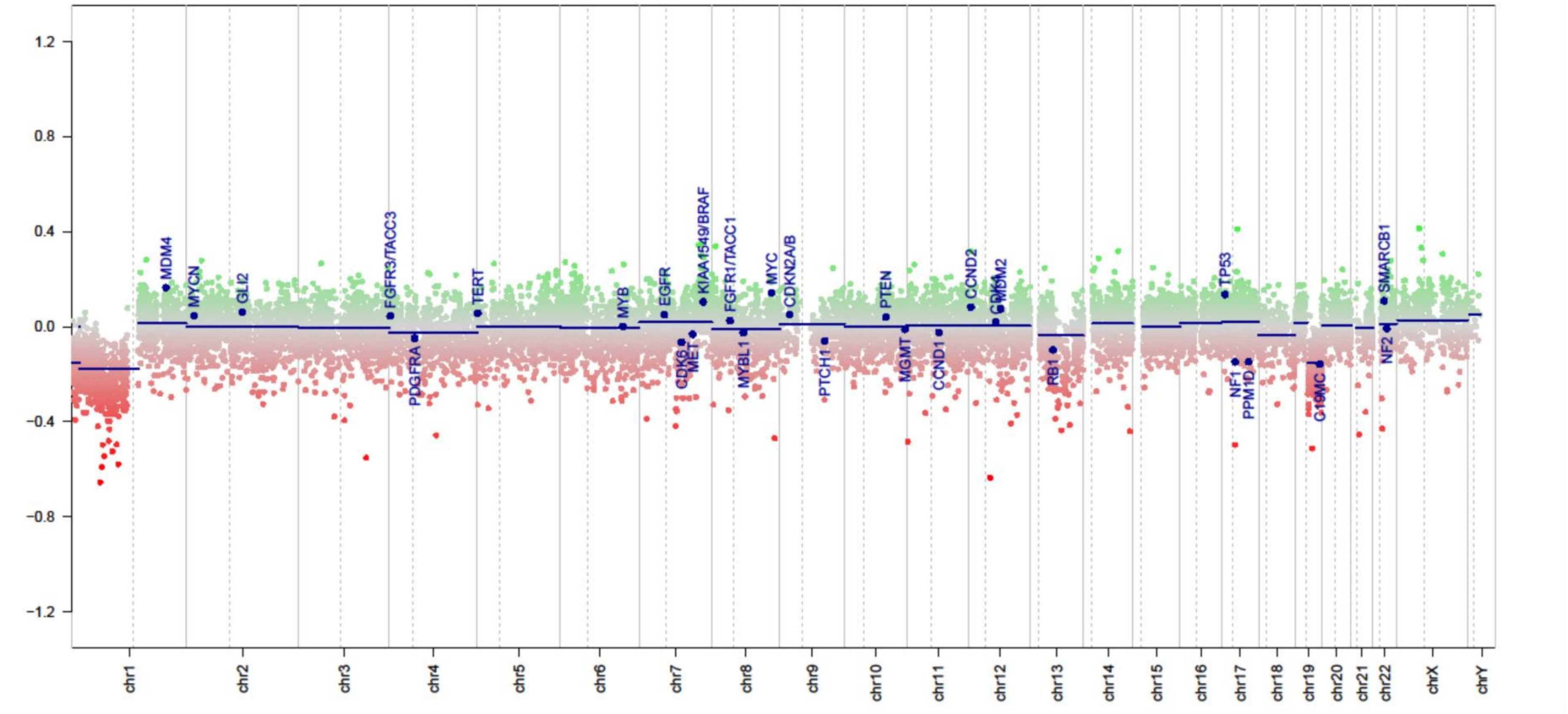

1.2
0.8
0.4
0.0
−0.4
−0.8
−1.2
MDM4
MYCN
GLI2
FGFR3/TACC3
PDGFRA
TERT
MYB
EGFR
CDK6
MET
KIAA1549/BRAF
FGFR1/TACC1
MYBL1
MYC
CDKN2A/B
PTCH1
PTEN
MGMT
CCND1
CCND2
CDK4
MDM2
RB1
TP53
NF1
PPM1D
C19MC
SMARCB1
NF2
chr1
chr2
chr3
chr4
chr5
chr6
chr7
chr8
chr9
chr10
chr11
chr12
chr13
chr14
chr15
chr16
chr17
chr18
chr19
chr20
chr21
chr22
chrX
chrY

LG-17

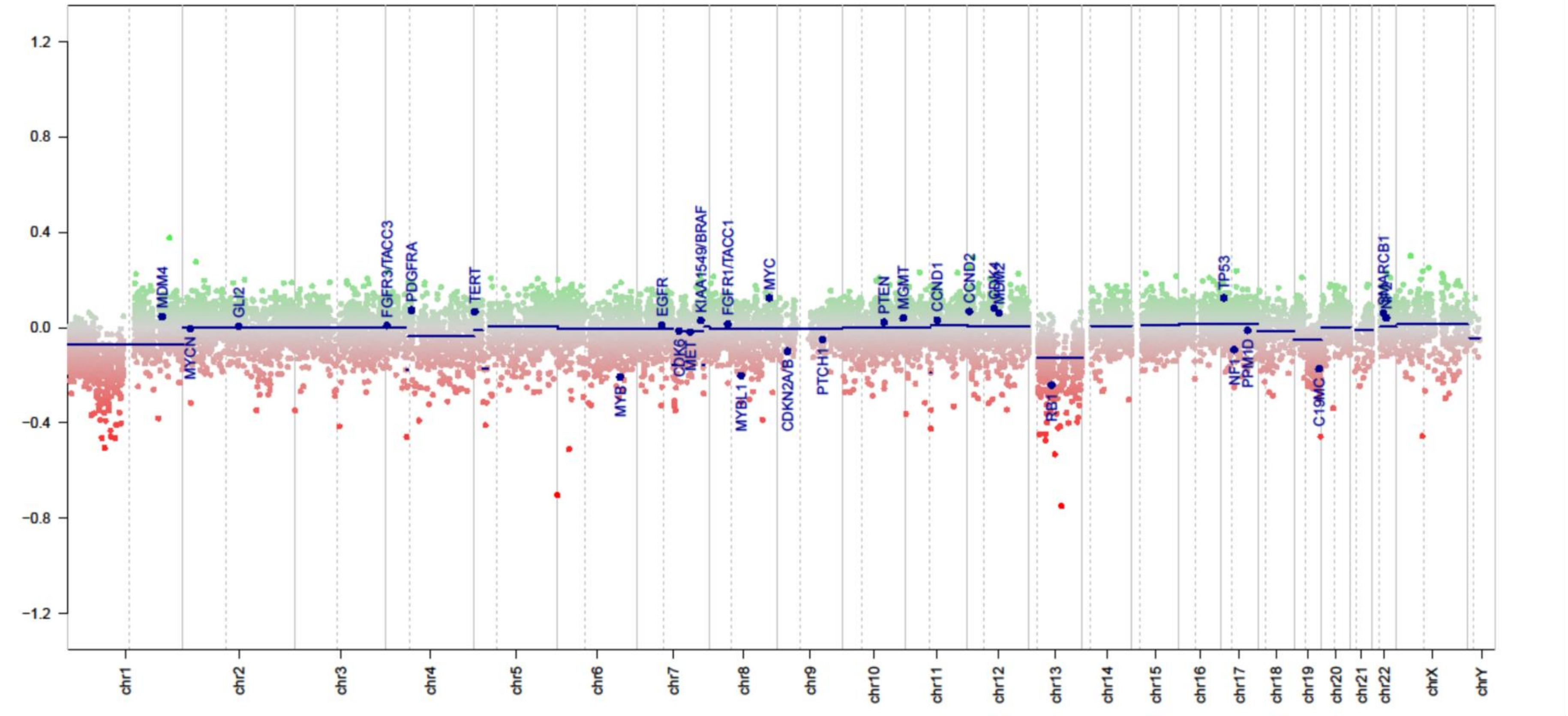
1.2
0.8
0.4
0.0
−0.4
−0.8
−1.2
MDM4
MYCN
GLI2
FGFR3/TACC3
PDGFRA
TERT
MYB
EGFR
CDK6
MET
KIAA1549/BRAF
FGFR1/TACC1
MYBL1
MYC
CDKN2A/B
PTCH1
PTEN
MGMT
CCND1
CCND2
CDK4
MDM2
RB1
TP53
NF1
PPM1D
C19MC
SMARCB1
chr1
chr2
chr3
chr4
chr5
chr6
chr7
chr8
chr9
chr10
chr11
chr12
chr13
chr14
chr15
chr16
chr17
chr18
chr19
chr20
chr21
chr22
chrX
chrY

LG-18

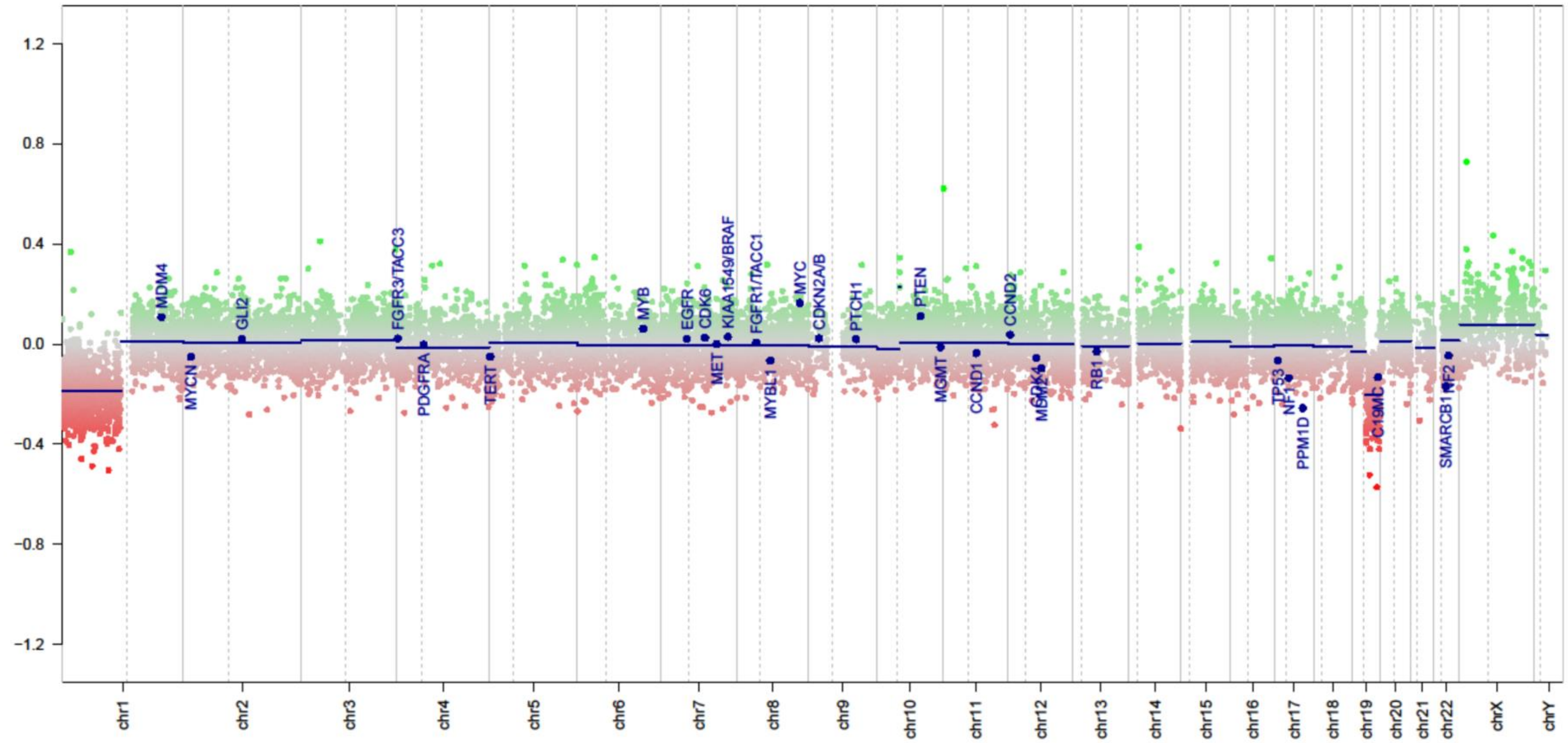

1.2
0.8
0.4
0.0
−0.4
−0.8
−1.2
MDM4
MYCN
GLI2
FGFR3/TACC3
PDGFRA
TERT
MYB
EGFR
CDK6
MET
KIAA1549/BRAF
FGFR1/TACC1
MYBL1
MYC
CDKN2A/B
PTCH1
PTEN
MGMT
CCND1
CCND2
CDK4
MDM2
RB1
TP53
NF1
PPM1D
C19MC
SMARCB1
NF2
chr1
chr2
chr3
chr4
chr5
chr6
chr7
chr8
chr9
chr10
chr11
chr12
chr13
chr14
chr15
chr16
chr17
chr18
chr19
chr20
chr21
chr22
chrX
chrY

LG-19

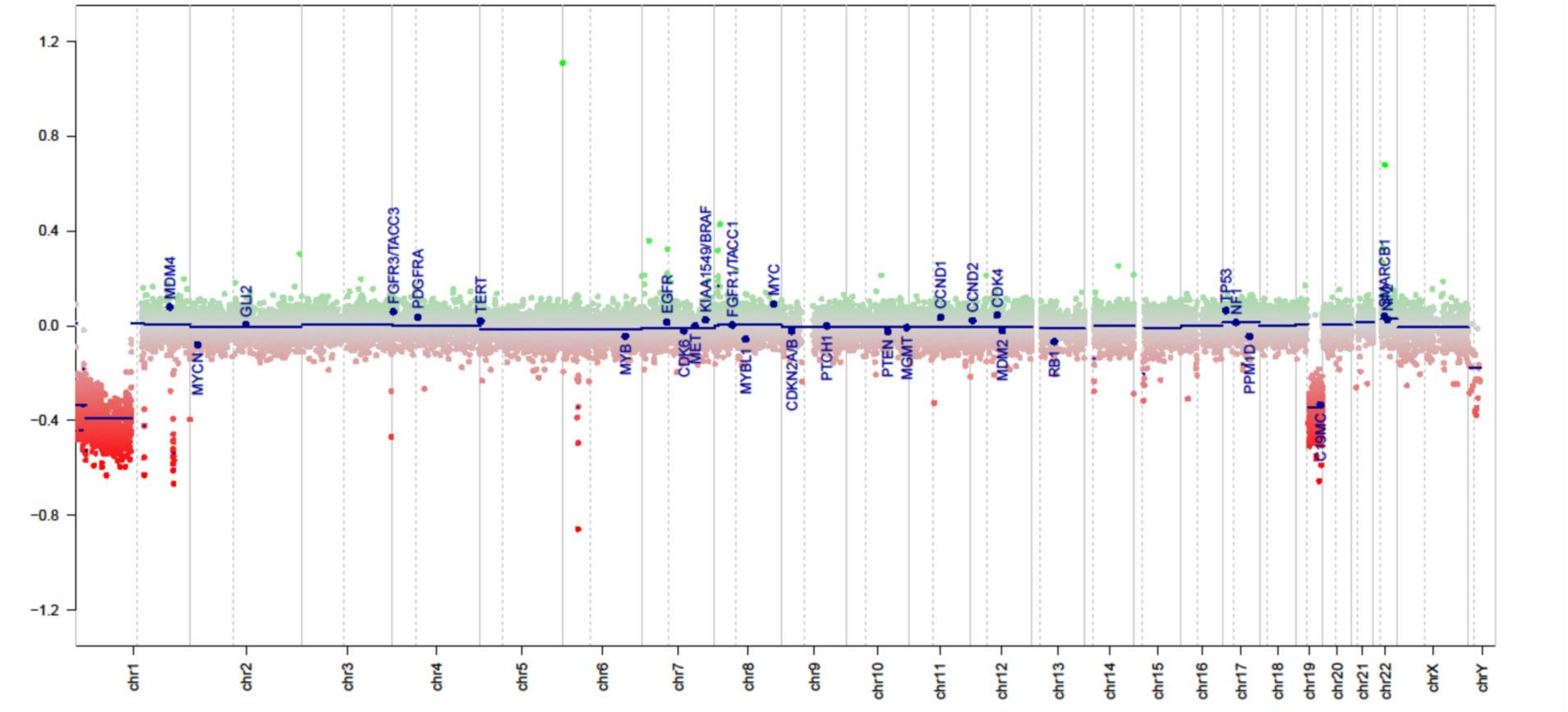

1.2
0.8
0.4
0.0
−0.4
−0.8
−1.2
MDM4
MYCN
GLI2
FGFR3/TACC3
PDGFRA
TERT
MYB
EGFR
CDK6
MET
KIAA1549/BRAF
FGFR1/TACC1
MYBL1
MYC
CDKN2A/B
PTCH1
PTEN
MGMT
CCND1
CCND2
CDK4
MDM2
RB1
TP53
NF1
PPM1D
C19MC
SMARCB1
chr1
chr2
chr3
chr4
chr5
chr6
chr7
chr8
chr9
chr10
chr11
chr12
chr13
chr14
chr15
chr16
chr17
chr18
chr19
chr20
chr21
chr22
chrX
chrY

LG-20

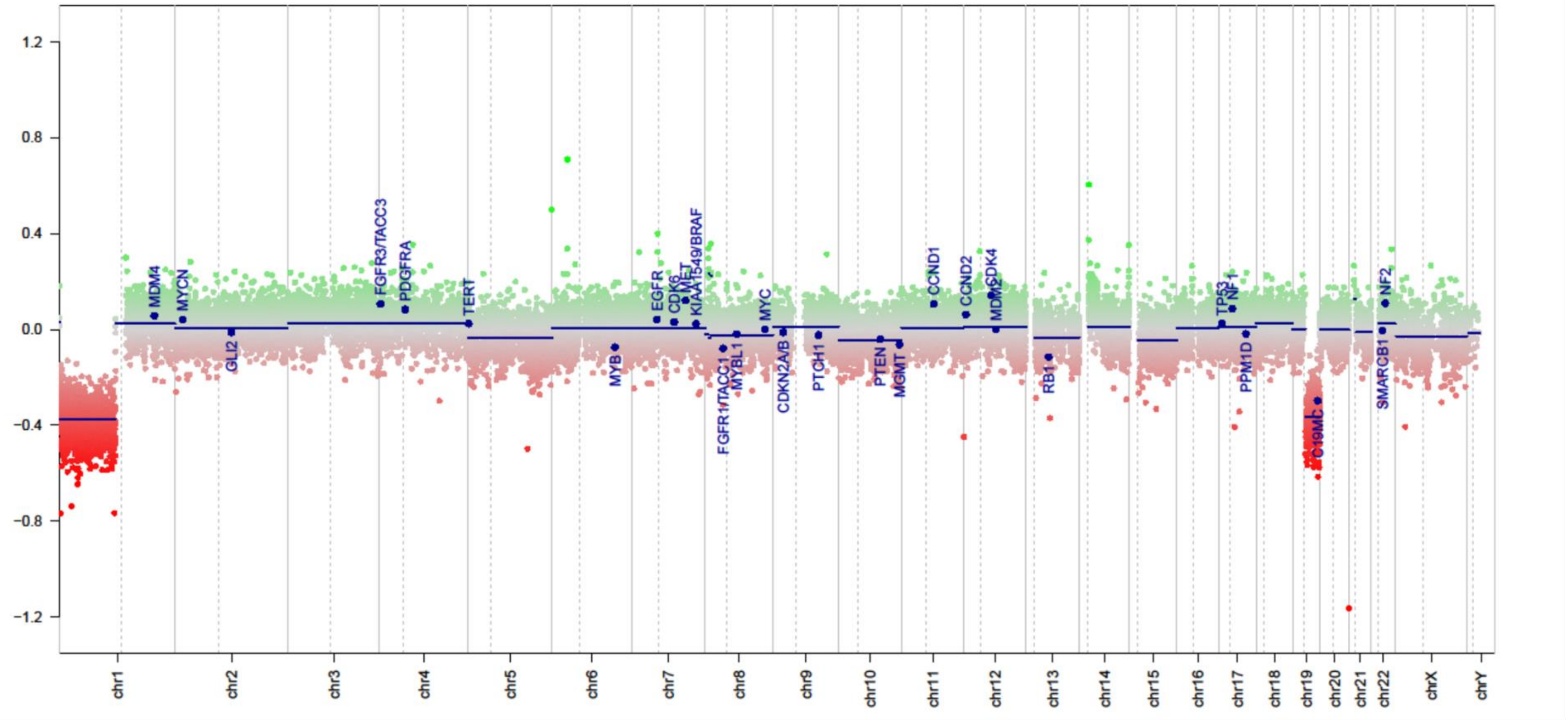
1.2
0.8
0.4
0.0
-0.4
-0.8
-1.2
MDM4
MYCN
GLI2
FGFR3/TACC3
PDGFRA
TERT
MYB
EGFR
CDK6
MET
KIAA1549/BRAF
FGFR1/TACC1
MYBL1
MYC
CDKN2A/B
PTCH1
PTEN
MGMT
CCND1
CCND2
CDK4
MDM2
RB1
TP53
NF1
PPM1D
C19MC
SMARCB1
NF2
chr1
chr2
chr3
chr4
chr5
chr6
chr7
chr8
chr9
chr10
chr11
chr12
chr13
chr14
chr15
chr16
chr17
chr18
chr19
chr20
chr21
chr22
chrX
chrY

LG-21

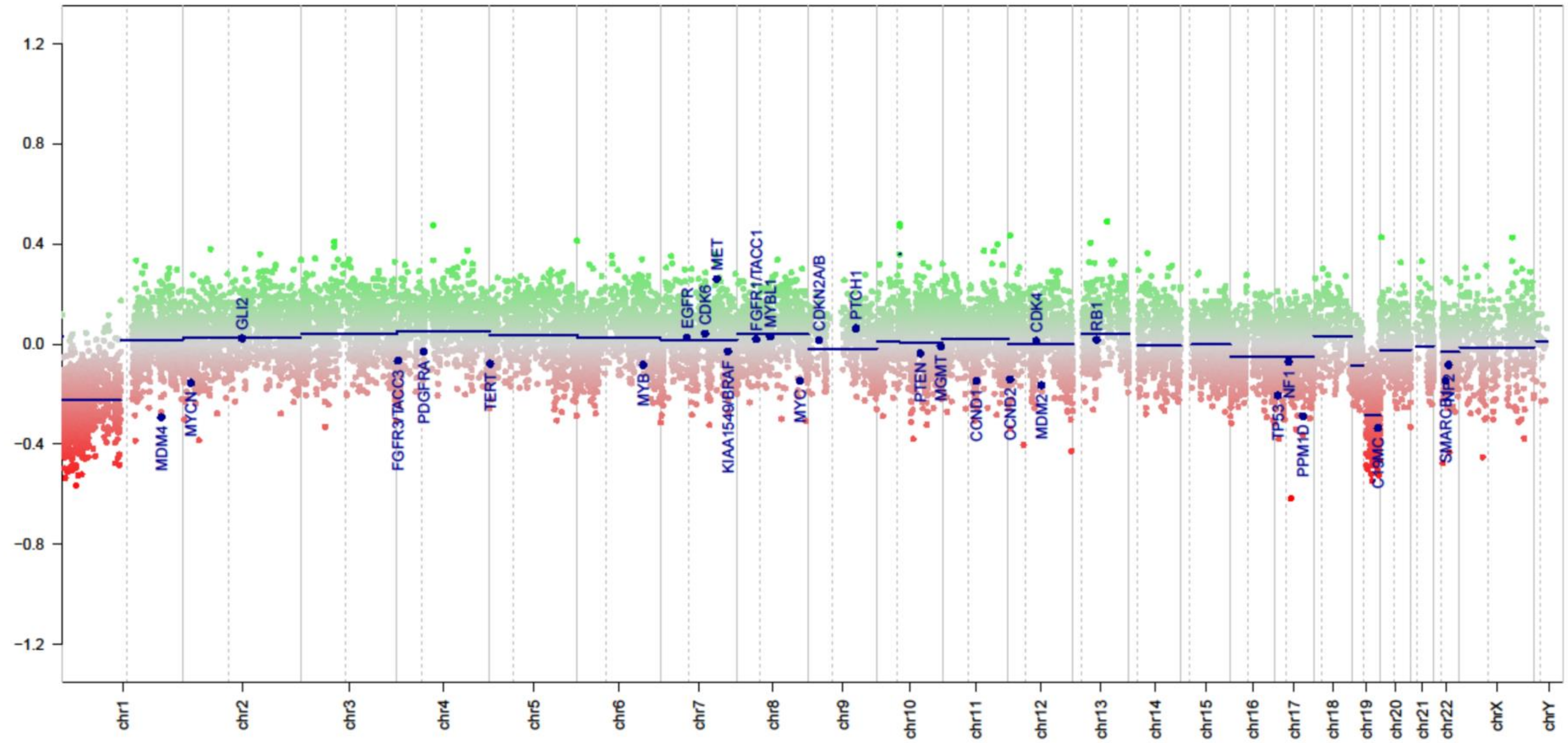

1.2
0.8
0.4
0.0
−0.4
−0.8
−1.2
MDM4
MYCN
GLI2
FGFR3/TACC3
PDGFRA
TERT
MYB
EGFR
CDK6
MET
KIAA1549/BRAF
FGFR1/TACC1
MYBL1
MYC
CDKN2A/B
PTCH1
PTEN
MGMT
CCND1
CCND2
CDK4
MDM2
RB1
TP53
NF1
PPM1D
SMARCB1
chr1
chr2
chr3
chr4
chr5
chr6
chr7
chr8
chr9
chr10
chr11
chr12
chr13
chr14
chr15
chr16
chr17
chr18
chr19
chr20
chr21
chr22
chrX
chrY

LG-22

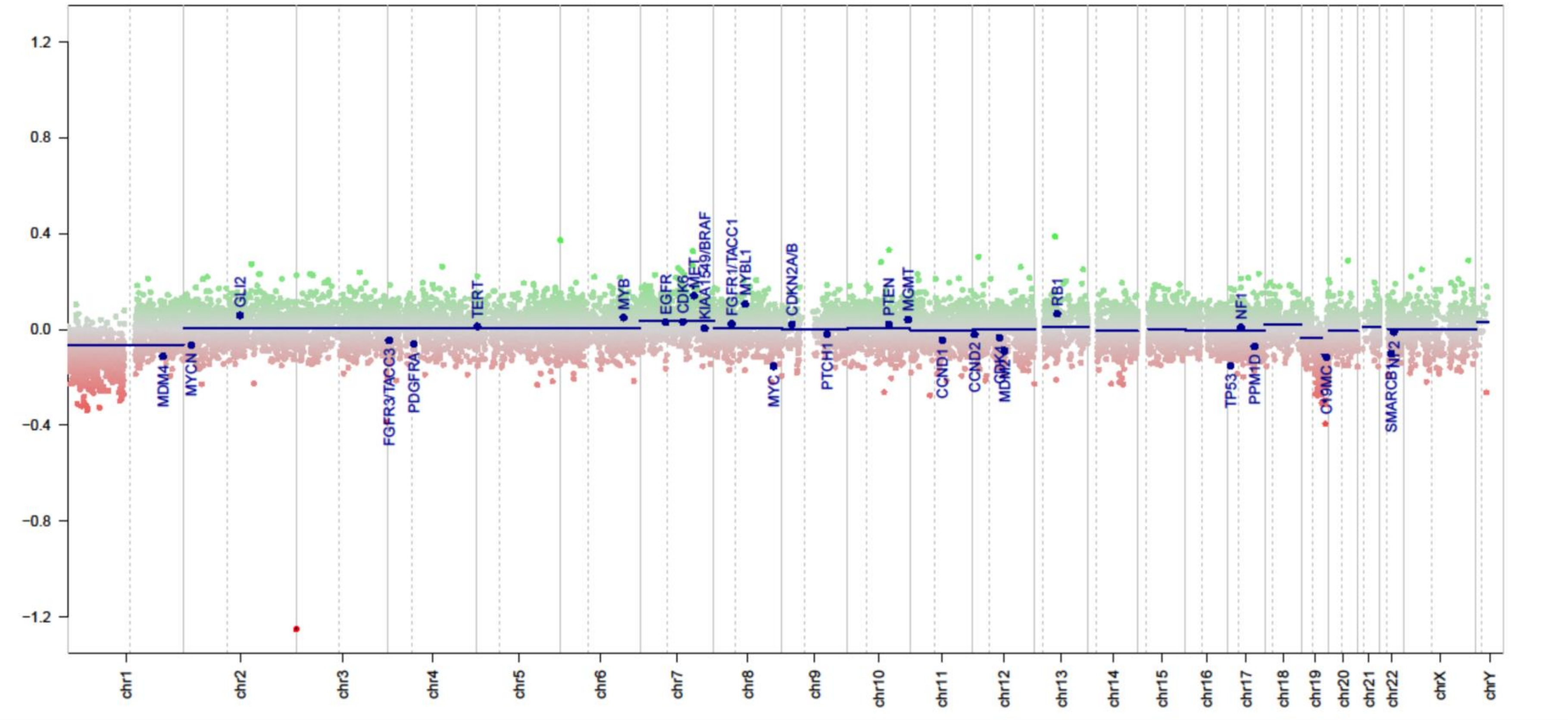

1.2
0.8
0.4
0.0
-0.4
-0.8
-1.2
MDM4
MYCN
GLI2
FGFR3/TACC3
PDGFRA
TERT
MYB
EGFR
CDK6
MET
KIAA1549/BRAF
FGFR1/TACC1
MYBL1
MYC
CDKN2A/B
PTCH1
PTEN
MGMT
CCND1
CCND2
CDK4
MDM2
RB1
TP53
NF1
PPM1D
C19MC
SMARCB1
NF2
chr1
chr2
chr3
chr4
chr5
chr6
chr7
chr8
chr9
chr10
chr11
chr12
chr13
chr14
chr15
chr16
chr17
chr18
chr19
chr20
chr21
chr22
chrX
chrY

LG-23

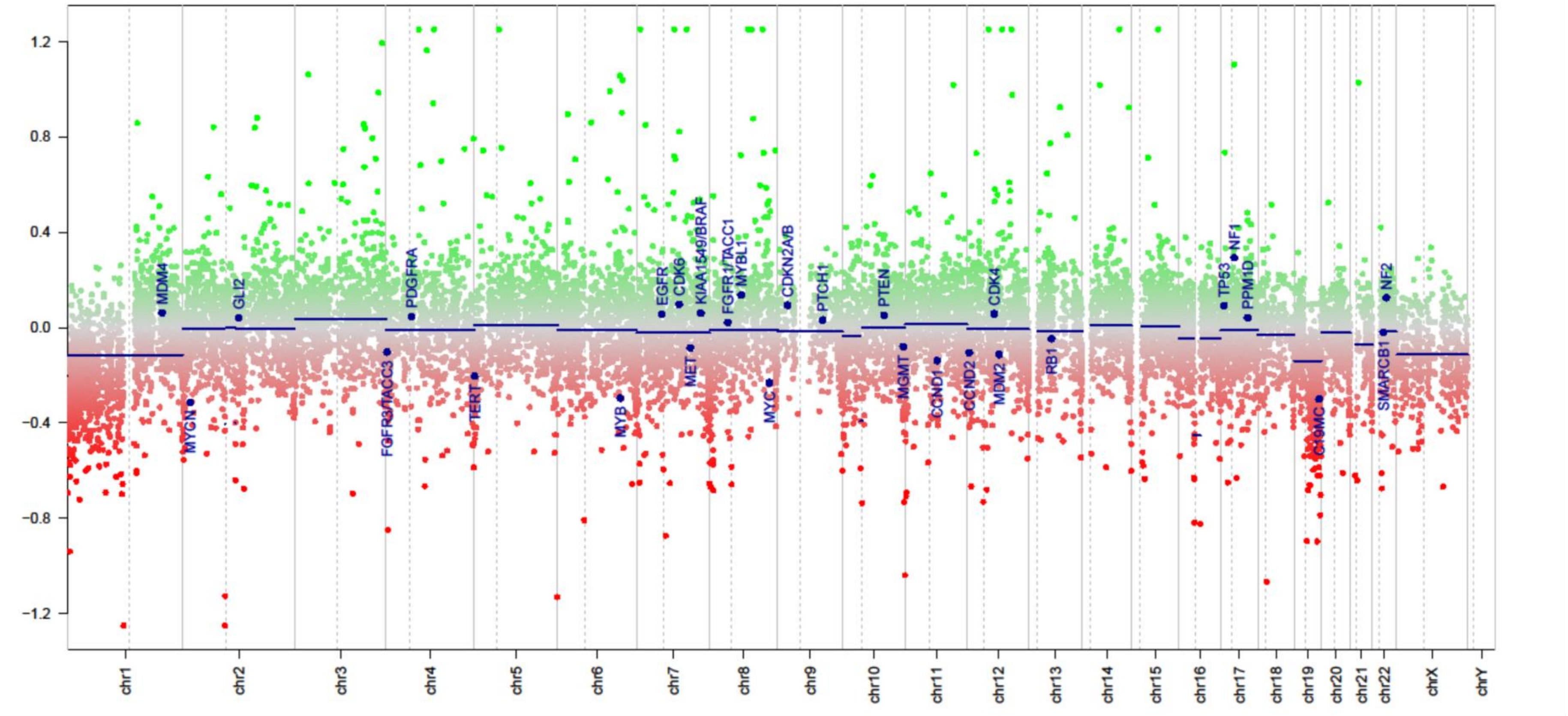

1.2
0.8
0.4
0.0
-0.4
-0.8
-1.2
MDM4
MYCN
GLI2
FGFR3/TACC3
PDGFRA
TERT
MYB
EGFR
CDK6
KIAA1549/BRAF
MET
FGFR1/TACC1
MYBL1
CDKN2A/B
MYC
PTCH1
PTEN
MGMT
CCND1
CCND2
CDK4
MDM2
RB1
TP53
NF1
PPM1D
C19MC
NF2
SMARCB1
chr1
chr2
chr3
chr4
chr5
chr6
chr7
chr8
chr9
chr10
chr11
chr12
chr13
chr14
chr15
chr16
chr17
chr18
chr19
chr20
chr21
chr22
chrX
chrY

LG-24

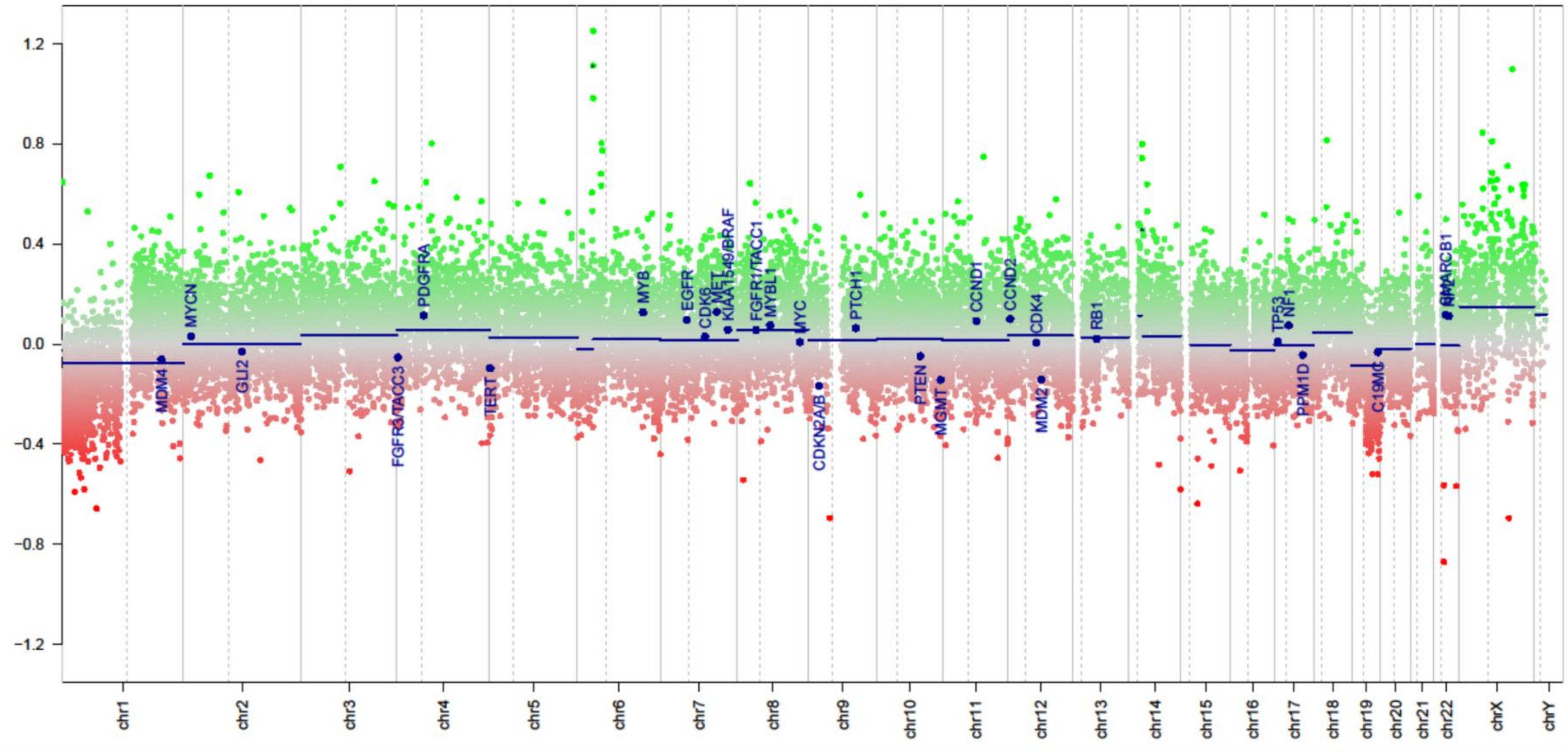
1.2
0.8
0.4
0.0
-0.4
-0.8
-1.2
MYCN
MDM4
GLI2
FGFR3/TACC3
PDGFRA
TERT
MYB
EGFR
CDK6
MET
KIAA1549/BRAF
FGFR1/TACC1
MYBL1
MYC
CDKN2A/B
PTCH1
PTEN
MGMT
CCND1
CCND2
CDK4
MDM2
RB1
TP53
NF1
PPM1D
C19MC
SMARCB1
chr1
chr2
chr3
chr4
chr5
chr6
chr7
chr8
chr9
chr10
chr11
chr12
chr13
chr14
chr15
chr16
chr17
chr18
chr19
chr20
chr21
chr22
chrX
chrY

LG-25

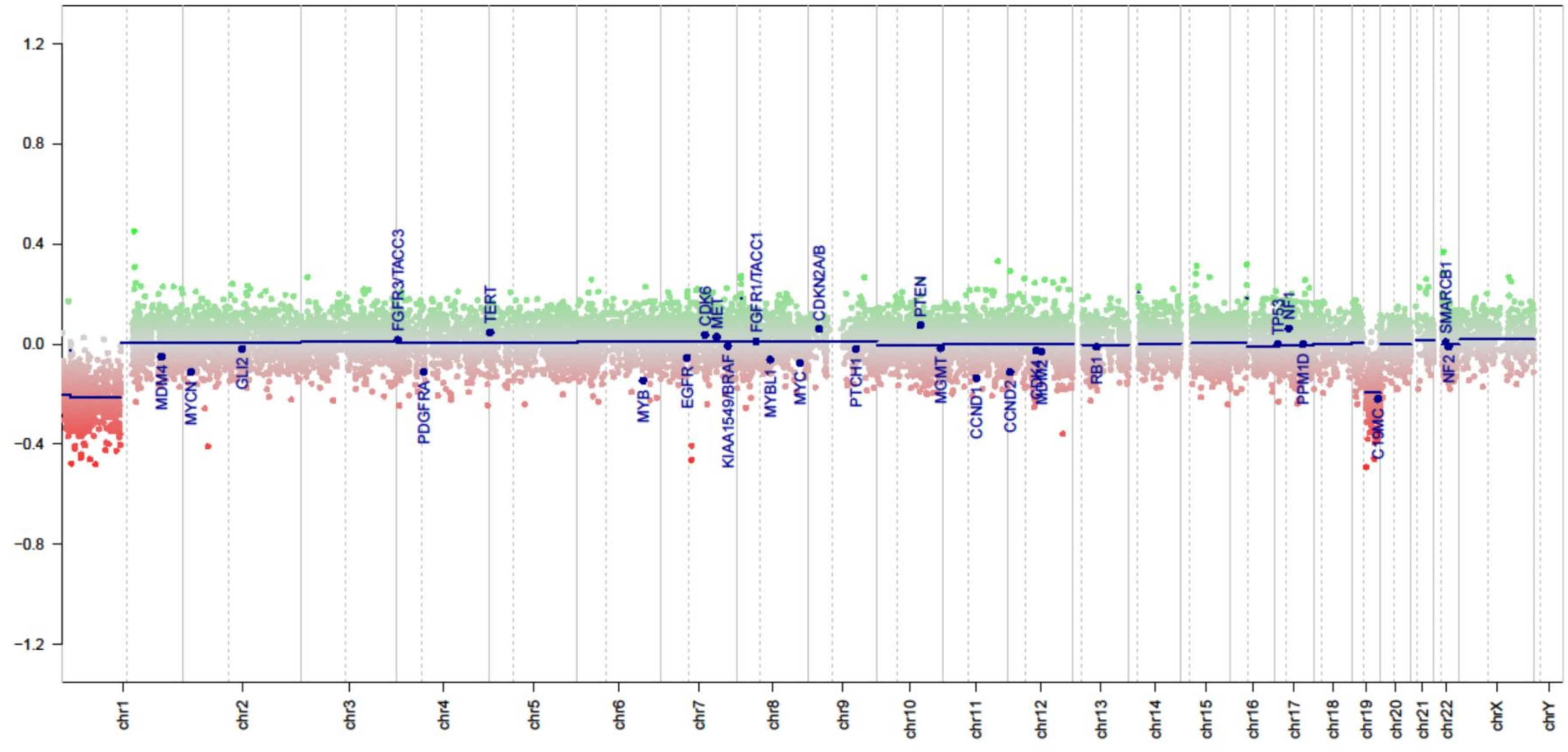
1.2
0.8
0.4
0.0
−0.4
−0.8
−1.2
FGFR3/TACC3
TERT
CDK6
MET
FGFR1/TACC1
CDKN2A/B
PTEN
TP53
NF1
SMARCB1
MDM4
MYCN
GLI2
PDGFRA
MYB
EGFR
KIAA1549/BRAF
MYBL1
MYC
PTCH1
MGMT
CCND1
CCND2
CDK4
MDM2
RB1
PPM1D
C19MC
NF2
chr1
chr2
chr3
chr4
chr5
chr6
chr7
chr8
chr9
chr10
chr11
chr12
chr13
chr14
chr15
chr16
chr17
chr18
chr19
chr20
chr21
chr22
chrX
chrY

LG-26

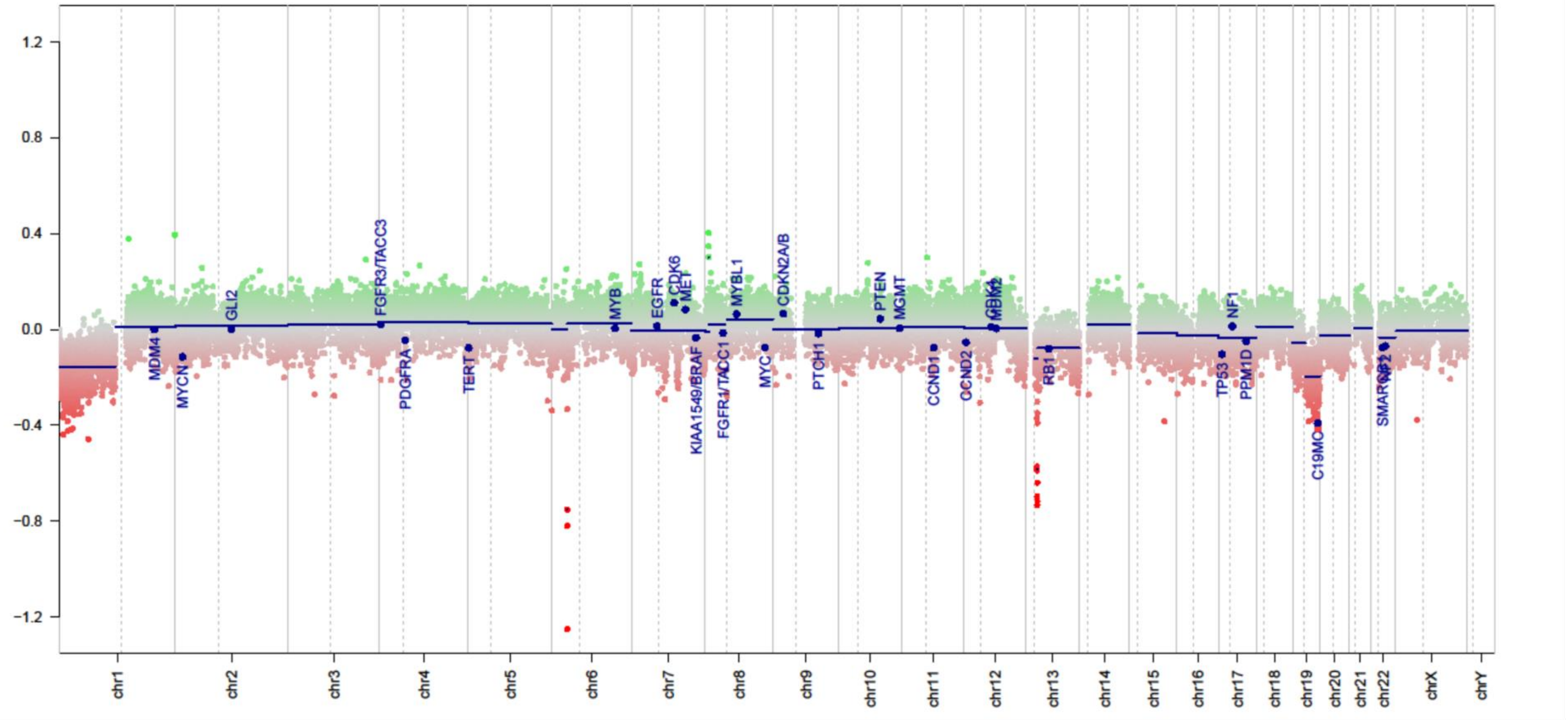
1.2
0.8
0.4
0.0
-0.4
-0.8
-1.2
MDM4
MYCN
GLI2
FGFR3/TACC3
PDGFRA
TERT
MYB
EGFR
CDK6
MET
KIAA1549/BRAF
FGFR1/TACC1
MYBL1
MYC
CDKN2A/B
PTCH1
PTEN
MGMT
CCND1
CCND2
CDK4
MDM2
RB1
TP53
NF1
PPM1D
C19MC
SMARCB1
NF2
chr1
chr2
chr3
chr4
chr5
chr6
chr7
chr8
chr9
chr10
chr11
chr12
chr13
chr14
chr15
chr16
chr17
chr18
chr19
chr20
chr21
chr22
chrX
chrY

LG-27

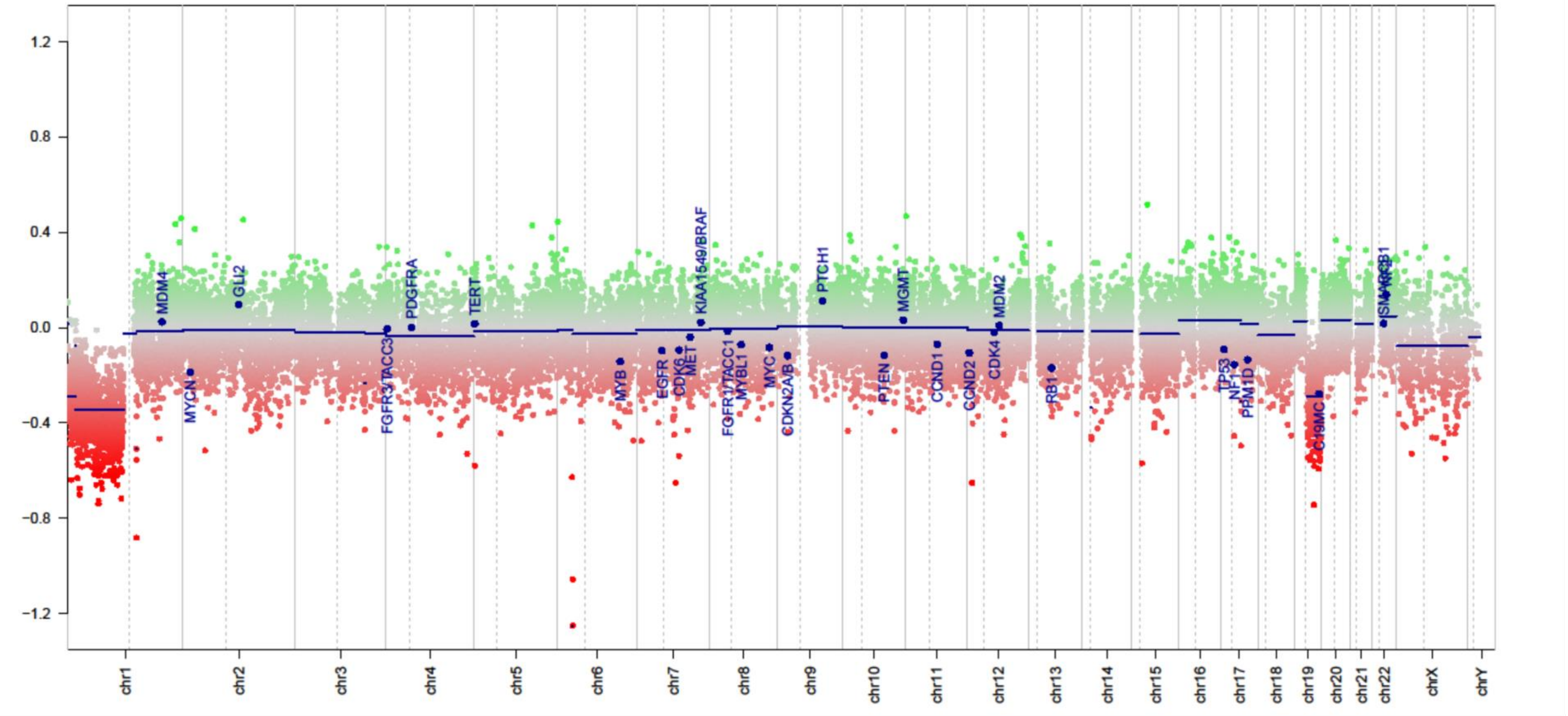

1.2
0.8
0.4
0.0
-0.4
-0.8
-1.2
MDM4
MYCN
GLI2
FGFR3/TACC3
PDGFRA
TERT
MYB
EGFR
CDK6
MET
KIAA1549/BRAF
FGFR1/TACC1
MYBL1
MYC
CDKN2A/B
PTCH1
PTEN
MGMT
CCND1
CCND2
CDK4
MDM2
RB1
TP53
NF1
PPM1D
C19MC
SMARCB1
chr1
chr2
chr3
chr4
chr5
chr6
chr7
chr8
chr9
chr10
chr11
chr12
chr13
chr14
chr15
chr16
chr17
chr18
chr19
chr20
chr21
chr22
chrX
chrY
